# Antiviral prophylaxis or preemptive therapy for cytomegalovirus after liver transplantation?: A systematic review and meta-analysis

**DOI:** 10.3389/fimmu.2022.953210

**Published:** 2022-11-10

**Authors:** Dipesh Kumar Yadav, Vishnu Prasad Adhikari, Rajesh Kumar Yadav, Alina Singh, Xing Huang, Qi Zhang, Prabesh Pandit, Qi Ling, Tingbo Liang

**Affiliations:** ^1^ Department of Hepatobiliary Surgery & Pancreatic Surgery, The First Affiliated Hospital, Zhejiang University, Hangzhou, China; ^2^ Zhejiang Provincial Key Laboratory of Pancreatic Disease, The First Affiliated Hospital, Zhejiang University, Hangzhou, China; ^3^ Zhejiang Provincial Innovation Center for the Study of Pancreatic Diseases, The First Affiliated Hospital, Zhejiang University, Hangzhou, China; ^4^ Zhejiang Provincial Clinical Research Center for the Study of Hepatobiliary & Pancreatic Diseases, The First Affiliated Hospital, Zhejiang University, Hangzhou, China; ^5^ Cancer Center, Zhejiang University, Zhejiang, China; ^6^ Department of Clinical Pharmacology, University of Louisiana at Monroe, Monroe, LA, United States; ^7^ Department of Surgery, Pokhara Medical Clinic, Pokhara, Nepal; ^8^ Department of Medicine, Kathmandu Medical College, Kathmandu, Nepal

**Keywords:** cytomegalovirus, liver transplantation, antiviral prophylaxis, CMV disease, preemptive therapy

## Abstract

**Background:**

To conduct a meta-analysis with the aim of comparing the outcomes of antiviral prophylaxis and preemptive therapy for the prevention of cytomegalovirus (CMV) infection in liver transplant (LT) recipients.

**Methods:**

We searched databases for qualified studies up until March 2022. Finally, a meta-analysis was carried out using a fixed-effect or random-effect model based on the heterogeneity.

**Results:**

With a total of 1834 LT patients, the pooled incidence of CMV infection and CMV disease in the overall LT recipients using antiviral prophylaxis and preemptive therapy were 24.7% vs. 40.4% and 6.4% vs. 9.4%, respectively. Our meta-analysis exhibited a significant reduction in the incidence of CMV infection due to antiviral prophylaxis when compared to preemptive therapy in the high-risk group (OR: 6.67, 95% CI: 1.73, 25.66; p = 0.006). In contrast, there was a significant reduction in the incidence of late-onset of CMV disease in preemptive therapy compared to antiviral prophylaxis in the high-risk group (OR: 0.29, 95% CI: 0.12, 0.74; p = 0.009). However, the incidence of CMV disease, allograft rejection, graft loss, drug related adverse effects, opportunistic infections and mortality did not differ significantly between both the interventions (all p> 0.05).

**Conclusions:**

We found the use of antiviral prophylaxis, compared with preemptive therapy, is superior in controlling CMV infection and prolonging the time to CMV disease in LT recipients without an increased risk of opportunistic infections, allograft rejection, graft loss, drug related adverse effects, development of drug resistance, and mortality.

## Introduction

Cytomegalovirus (CMV) in humans, also known as human CMV or human herpesvirus 5, globally infects about 60% to 100% of humans (1). Similar to herpesvirus, CMV prevails for the rest of life in an infected individual, particularly by setting latency in the tissue endothelial cells and bone marrow haematopoietic progenitor cells (2, 3). Though, most of the healthy individuals infected with CMV are asymptomatic; however, it is important to note that CMV entails significant morbidity and mortality among the immunocompromised individuals like solid organ transplant (SOT) or stem cell transplant recipients and human immunodeficiency virus (HIV) positive patients (4).

In liver transplant (LT) recipients, the natural history and pathogenesis of CMV infection is complicated, and infection might be as a result of a primary infection, superinfection or reactivation of the latent infection despite having innate immunity in the host (5). According to the prevalence of IgG antibodies LT recipients can traditionally be grouped into four subgroups, i.e., donor seropositive/recipient seronegative (D+/R–), donor seropositive/recipient seropositive (D+/R+), donor seronegative/recipient seropositive (D-/R+), and donor seronegative/recipient seronegative (D-/R-). In a study it was perceived that, primary infection invariably occurred in 88% of the D^+^/R^–^ LT recipients (high-risk group), subsequently viremia appeared in 57% of the D^+^/R^+^ LT recipients either by reactivation or reinfection (intermediate-risk group), while viremia developed in 36% of the D^-^/R^+^ LT recipients (low-risk group) typically due to reactivation of the latent CMV, and eventually no viremia was seen in the D^-^/R^-^ LT recipients (5).

Currently, various strategies like preemptive therapy, antiviral prophylaxis, hybrid approaches (continuous surveillance after prophylaxis for CMV viremia with preemptive therapy), and CMV-specific immunity-guided approaches are being used for effective control of CMV infection in the LT alike in other SOT (6, 7). However, antiviral prophylaxis and preemptive therapy are the most commonly used strategies in many centers. In antiviral prophylaxis, antiviral drugs are routinely administered to all transplant recipients at a risk of CMV disease, typically for 3 months or more immediately after transplantation. While in a preemptive therapy strategy, antiviral drugs are merely given to those transplant recipients who ideally possess a sufficient evidence of CMV viremia in an attempt to prevent CMV disease until negative tests (8). Studies on LT recipients using antiviral prophylaxis and preemptive therapy for prevention of CMV have yielded conflicting results on the outcomes of these strategies (9–12). Though antiviral prophylaxis is widely used by many centers and recommended strategy by the American Society of Transplantation, (8) post-prophylaxis CMV disease (late-onset of CMV disease) remains a well-documented and widespread problem in LT recipients receiving antiviral prophylaxis, and is found to be independently associated with mortality (9, 12). Preemptive strategy on the other hand has been shown to decrease the incidence of late-onset CMV disease and increase in CMV-induced immune response (6, 9). However, preemptive strategy essentially faces logistics challenges for medical centers and patient’s noncompliance with monitoring of CMV viremia (7).

To our knowledge, until the time of writing this article, only two earlier meta-analysis comparing antiviral prophylaxis and preemptive therapy for the prevention of CMV infection in LT recipients had been reported. However, both of them lack statistical power due to indirect comparison (13) and a lesser number (14) of included studies. Recently, a significant number of direct comparative studies in this area have been published, and no new meta-analysis has been carried out to summarize the available findings in depth. This meta-analysis includes a larger sample size and compares antiviral prophylaxis and preemptive therapy for CMV in LT recipients stratified according to the CMV serostatus with the aim of properly assessing CMV disease, clinical outcomes, drug related adverse effects, and CMV-specific immune responses.

## Materials and methods

### Search strategy for the identification of the studies

Our systematic review and meta-analysis was conducted according to the Preferred Reporting Items for Systematic Reviews and Meta-Analyses (PRISMA) Statement (15). Three authors (DKY, VPA and RKY) independently searched databases like PubMed, EMBASE, Scopus, Cochrane Library databases and Web of Sciences for the relevant studies with an earlier agreed protocol. The search was performed till March 2022 and was limited to, the studies published in English only. Following Medical Subject Headings (MeSH) and non-MeSH terms, “cytomegalovirus or CMV,” “liver transplant and CMV,” “liver transplant or liver graft,” “universal or prophylaxis,” “prophylactic and preemptive,” “liver transplant,” “solid organ transplant,” “antivirus,” “viral infection” were used to carry out the search for the relevant articles in the database. Additionally, we also searched the reference lists within the reviewed articles to identify more relevant studies.

### Eligibility criteria

Considering the purpose of our study and to secure the quality of this meta-analysis, only fully published direct comparative studies between the antiviral prophylaxis and preemptive treatment for CMV in LT recipients using Ganciclovir or Valganciclovi an antiviral drug was considered. Studies such as randomised controlled trials (RCTs) and observational studies (both retrospective and prospective studies) were evaluated. We excluded publications like reviews, editorials, case studies, conference letters, studies without human subjects, studies with duplicate data from the same institution, studies with multi-organ transplant, studies not comparing antiviral prophylaxis and preemptive therapy for CMV in LT recipients.

The inclusion criteria were as follows: (a) study population: studies with LT recipients of all ages (both adult and paediatric) undergoing deceased donor liver transplantation (DDLT) or living donor liver transplantation (LDLT) (b) comparative studies: studies that compared antiviral prophylaxis (given for 3 months or more) with preemptive therapy using Ganciclovir or Valganciclovi as an antiviral drug in LT recipients for CMV.

### Data extraction and outcomes

EndNote X 8.0 was used to exclude all the duplicate studies. Three investigators (DKY, VPA and RKY) who were involved in the literature search also separately collected the data from the eligible studies. In the case of an insufficient detail, the authors of the included studies were contacted for more relevant data. Disagreements were resolved with the other investigators after discussion. Microsoft Excel was used to record all available information like author, year of study, institution, country, study design and characteristics, sample size, patient demographics, comorbidities, the number of participants in antiviral prophylaxis and preemptive therapy group, antiviral drugs (dose and duration), plasma CMV DNA load after an established infection, outcomes (CMV disease, graft loss, acute and chronic rejection, opportunistic infections, adverse effects of antiviral drugs, drug-resistance, CMV-related mortality, and all mortality), time to develop CMV infection, time to develop CMV disease, CMV-specific immune responses, baseline immunosuppressants, and the follow-up time.

The purpose of this meta-analysis was to compare incidence of CMV infection, CMV disease, mean time to CMV infection and disease, the indirect effects of CMV infection (acute rejection, graft loss, opportunistic infections), adverse drug events, development of CMV-specific immune responses, development of drug-resistance, and mortality (CMV-related mortality and all mortality) between the antiviral prophylaxis and preemptive therapy for CMV in LT stratified according to the CMV serostatus.

### Definitions

For the purpose of this meta-analysis, standard definitions were employed as recommended by the American Society of Transplantation (8). CMV infection was defined as the presence of CMV in any tissue or body fluid by using a CMV assay [polymerase chain reaction (PCR) or phosphoprotein 65 (pp65)] regardless of symptoms. CMV disease was defined as CMV infection together with a clinical sign and symptoms (a. CMV syndrome- fever, malaise, leukopenia or neutropenia, thrombocytopenia and bone marrow suppression; and b. End organ disease). Late-onset of CMV disease was defined as an onset of CMV disease after 100 days of transplantation. Acute antibody-mediated rejection (aAMR) was defined according to the Banff criteria based on liver biopsy, (16) and was considered for up to 12 months. Mortality was defined as a death from any cause during the follow-up after LT. Death within 6 weeks from the diagnosis of CMV disease or CMV detected on autopsy investigation was considered as CMV-associated mortality (17). Opportunistic infections were defined as per the included studies.

Antiviral prophylaxis was defined as the administration of the antiviral drugs to LT recipients to prevent CMV disease for 3 months or more immediately after LT. Likewise, preemptive therapy was defined as the administration of the antiviral drugs in LT recipients for the prevention of CMV disease only after the detection of CMV viremia/antigenemia using CMV assay (PCR or pp65) until negative tests.

### Statistical analysis of data

After double-checking of the data from the selected studies, we carried out the meta-analysis using OpenMeta Analyst for a pooled analysis and RevMan Version 5.3 (Review Manager, Copenhagen: The Nordic Cochrane Centre, The Cochrane Collaboration, 2014) for a pairwise comparison analysis. Depending upon the degree of heterogeneity, the meta-analysis was carried out using a fixed-effect or random-effect models. We used the Z-test to evaluate an overall effect, and Cochran’s χ2 test to assess heterogeneity. The degree of heterogeneity was classified according to the I^2^ statistic (i.e., low heterogeneity: I^2^ > 25%, moderate heterogeneity: I^2^ > 50%, and high heterogeneity: I^2^ > 75%). P< 0.05 was considered statistically significant. Additionally, we also carried out a sensitivity analysis by conducting a leave-one-out study to determine the statistical robustness that might have contributed to heterogeneity and have had a large influence on the final results. The results of meta-analysis for dichotomous outcomes were expressed as odds ratios (ORs), adverse effects were computed as risk differences (RD), and continuous outcomes were calculated as mean differences (MD) with 95% confidence intervals (CIs).

### Assessment of the risk of bias

The Newcastle-Ottawa scale (NOS) was used to assess the quality of included studies in our meta-analysis. (18) The NOS composes 3 evaluation items (1): evaluation of a collection of the study categories (2); comparability between the 2 categories; and (3) outcome evaluation. The scores in the NOS range between 0 to 9 (high quality study: scores ≥ 7 points; moderate quality study: scores between 4 to 6 points; and low quality study: scores ≤ 4). (**Supplementary Table 1**) In addition to NOS, we also ruled out any publication bias using funnel plots. (**Supplementary Figure 1**).

## Results

### Study search and included studies

**Figure 1:**
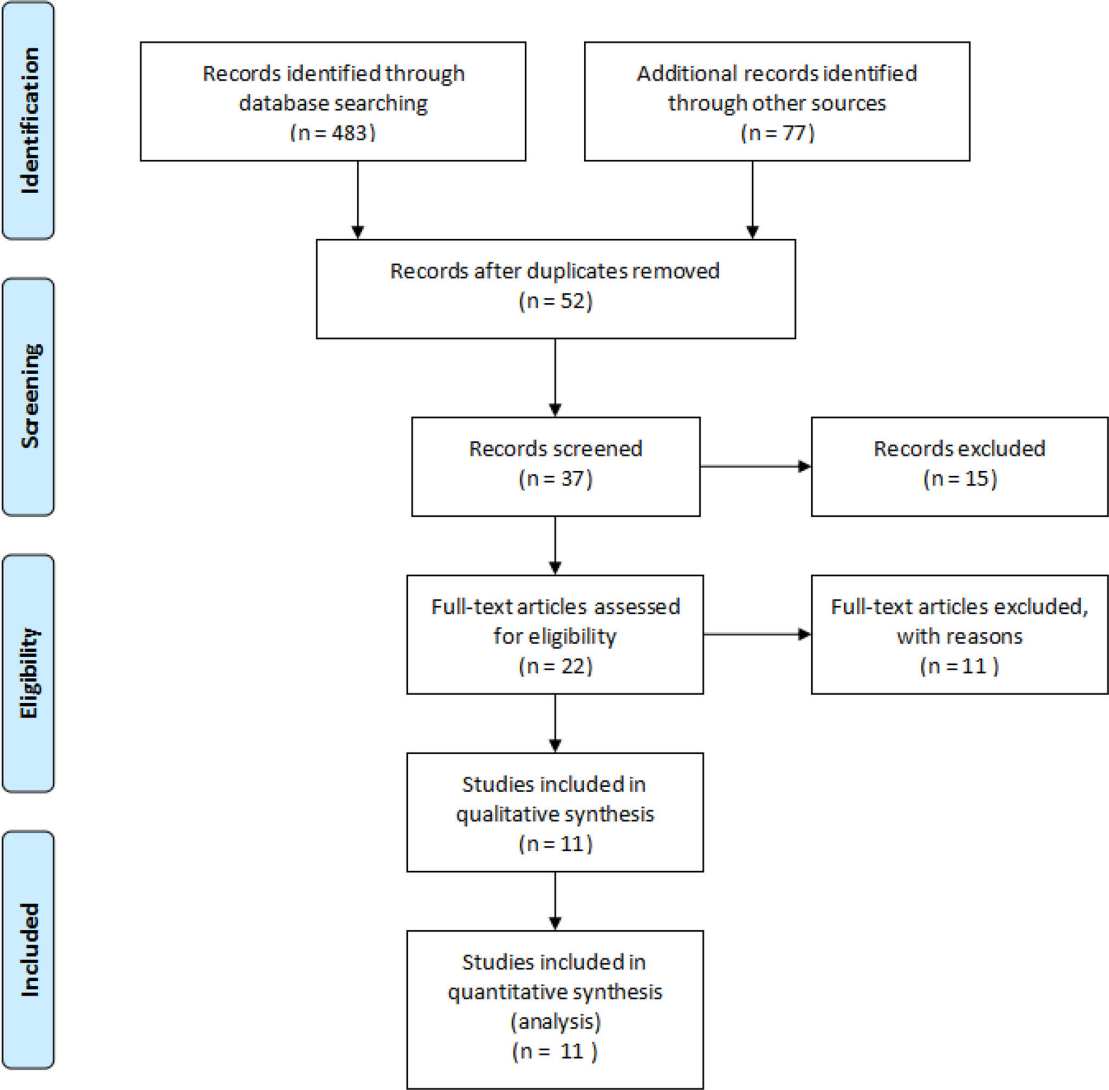
Preferred reporting items for the systematic review and meta-analysis study flow diagram for the literature search.

Afer the data base scans, we recognized 560 references for assessment. Of these, 52 full-text article were identified and were evaluated according to the inclusion criteria. We excluded 41 full-text articles for not meeting our inclusion criteria or those with an insufficient data. The remaining 11 full-text studies (9–12, 19–25), with a total of 1834 LT patients were eligible for this meta-analysis (**Figure 1**). **Table 1** shows the main characteristics of the eligible studies included in our meta-analysis. The eight included studies scored between 7 and 9. According to the NOS assessment, all the included studies were considered to have a low risk of bias in selection. Out of 11 studies, 3 were from USA, (9, 11, 12) 2 were from Germany, (19, 23) 1 was from Spain, (12) 1 was from France, (20) 1 was from Italy, (24) 1 was from Australia, (21) 1 was from Korea, (25) and 1 was from China (10). There was two RCT (9, 21), one prospective study,(11) and the remaining all eight were retrospective studies (10, 12, 19, 20, 22–25) Out of which seven studies reported no funding (11, 19–24), and the remaining four studies were funded from the national grants (9, 10, 12, 25), among which one RCT (9) reported the authors serving as a site investigator for clinical trials were sponsored by pharmaceutical and biotech companies.

**Table 1:** The characteristics of included studies.

Author, country, year	Study type	Total patients (n)		CMV monitoringmethod	Incidence of CMV infection/CMV reactivation (n)		Incidence of CMV disease (n)		Graft rejection (n)		OpportunisticInfections (n)		Graft loss (n)		Mortality (n)		Follow up (months)	NOS
		*Prophylaxis*	*Preemptive*		*Prophylaxis*	*Preemptive*	*Prophylaxis*	*Preemptive*	*Prophylaxis*	*Preemptive*	*Prophylaxis*	*Preemptive*	*Prophylaxis*	*Preemptive*	*Prophylaxis*	*Preemptive*		
Singh et al, USA, 2020 (9)	RCT	105	100	CMV PCR assay	NA	NA	20	9	33	37	33	36	2	4	20	15	>12 months	9
Liu et al, USA, 2018 (11)	Prospective	156	160	CMV PCR assay	NA	114	3	3	25	31	17	17	3	5	12	8	>12 months	8
Bodro et al, Spain, 2012 (12)	Retrospective	35	39	pp65 antigen assay	3	15	5	19	6	6	1	4	NA	NA	2^a^	5^a^	>12 months	9
Simon et al, Germany, 2016 (19)	Retrospective	60	68	CMV PCR assay	1	2	2	14	NA	NA	40	46	10	7	18	27/67^b^	>12 months	7
Mengelle et al, France, 2015 (20)	Retrospective	56	73	CMV PCR assay	18	42	4	7	13	35	NA	NA	NA	NA	11	17	>12 months	7
Scott et al, Australia, 2011 (21)	Retrospective	39	25	CMV PCR assay	12	12	3	0	NA	NA	NA	NA	NA	NA	NA	NA	>12 months	6
Onor et al, USA, 2013 (22)	Retrospective	61	48	CMV PCR assay	17	24	0	48	5	3	35	34	NA	NA	3	1	6-months	6
Lindner et al, Germany, 2016 (23)	Retrospective	21	26	CMV PCR assay	1	3	1	1	2	2	NA	NA	NA	NA	1	7	>12 months	9
Nicastro et al, Italy, 2016 (24)	Retrospective	16	100	CMV PCR assay	10	61	1	3	6	31	NA	NA	3	4	2	3	>60 months	6
Kim et al, Korea, 2012 (25)	Retrospective	281	281	. CMV PCR assay	42	32	NA	NA	NA	NA	NA	NA	NA	NA	37	52	>12 months	7
Lianghui et al, China, 2004 (10)	Retrospective	41	48	pp65 antigen assay	25	26	5	15	16	23	NA	NA	NA	NA	0	1	>12 months	7

CMV, Cytomegalovirus; PCR, Polymerase Chain Reaction; pp65, Phosphoprotein 65; NOS, Newcastle-Ottawa Quality Assessment Scale. , NA, not available.

^a^case fatality.

^b^one censored case.

### Meta-analysis

To perform a meta-analysis, we stratified studies based on the risk subgroups, i.e., high-risk group (D^+^/R^-^) (9, 12), LT recipients, intermediate-risk group (19, 20, 23) (studies that included both the intermediate-risk and low-risk group, i.e., D^+^/R^+^ and D^-^/R^+^ LT recipients in combined), and all-risks group (10, 11, 21, 22, 24, 25) (studies that included all the risk groups, i.e., D^+^/R^-^, D^+^/R^+^, and D^-^/R^+^ LT recipients in combined. One study by Lu et al.,(11) which only combined the high-risk and intermediate-risk group together was also placed in our all-risks group.) and compared antiviral prophylaxis and preemptive therapy for the outcomes. There were no studies that separately analysed the intermediate-risk and low-risk group. Additionally, we also carried out a pooled estimate of the incidence of events due to CMV in the overall LT recipients (combining all included studies together in this meta-analysis) and stratified groups as above. (**Supplementary Tables 2**, **3**).

### Incidence of CMV infection and CMV disease

The pooled results showed the rate of CMV infection in the overall LT recipients and intermediate-risk group receiving antiviral prophylaxis and preemptive therapy were 24.7% vs. 40.4% (9 vs. 10 studies and 610 vs. 868 patients) and 11.9% vs. 23.8% (3 vs. 3 studies and 137 vs. 167 patients), respectively. (**Supplementary Figure 2**) There was only one study reporting the incidence of CMV infection in the high-risk group for LT recipients receiving antiviral prophylaxis and preemptive therapy, i.e., 8.6% vs. 38.5% (12).

Likewise, our pooled analysis revealed the incidence of CMV disease in the overall LT recipients, high-risk group, and intermediate-risk group receiving antiviral prophylaxis and preemptive therapy were 6.4% vs. 9.4% (10 vs. 10 studies and 590 vs. 687 patients), 17.6% vs. 28.2% (2 vs. 2 studies and 140 vs. 139 patients), and 4.6% vs. 10.8% (3 vs. 3 studies and 137 vs. 167 patients), respectively (**Supplementary Figure 3**).

**Figure 2:**
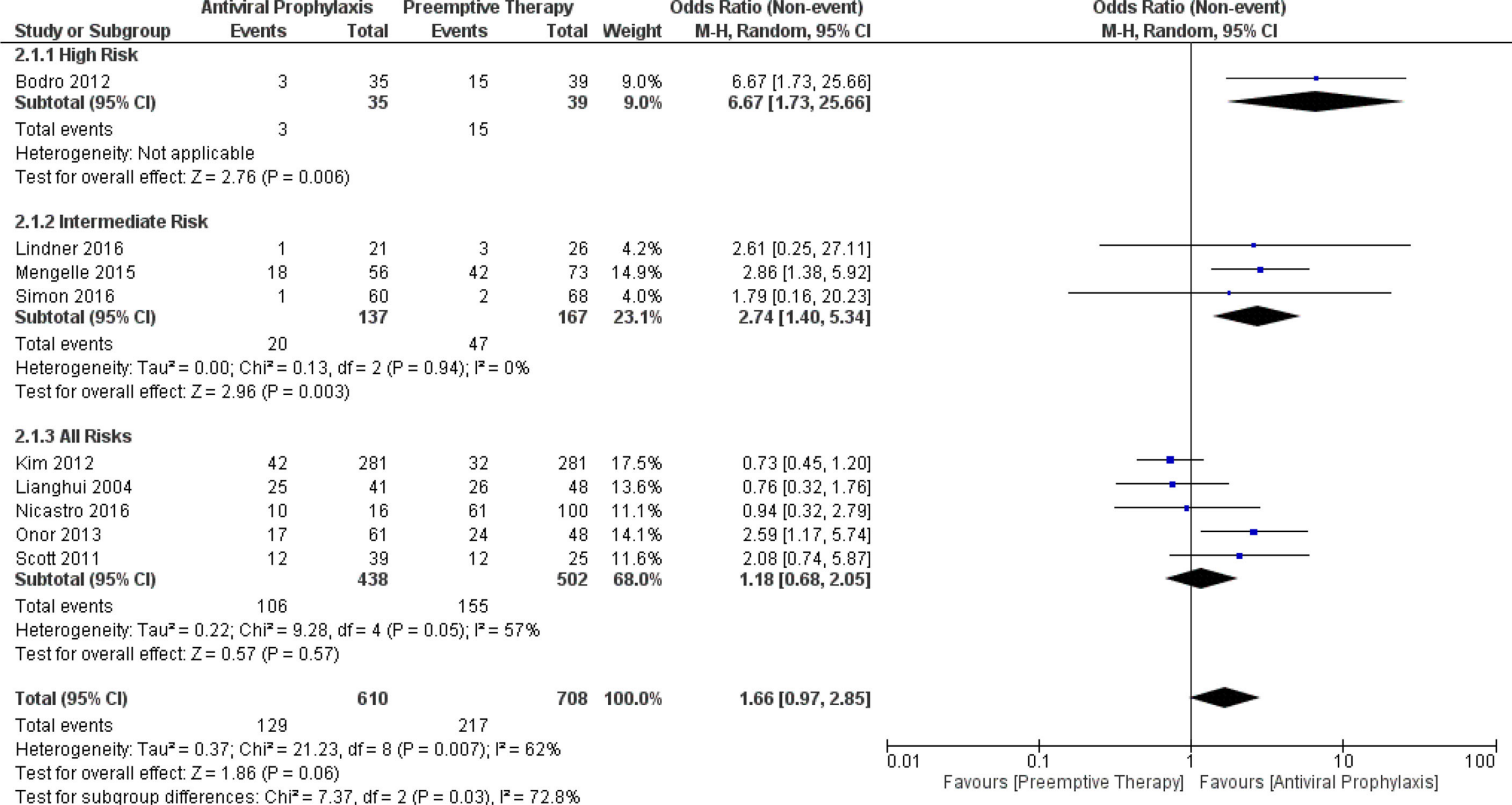
Forest plot depicts a comparison of the incidence of CMV infection among LT recipients undergoing antiviral prophylaxis and preemptive therapy.

Additionally, we also analysed the incidence of CMV infection and CMV disease amidst antiviral prophylaxis and preemptive therapy. Our meta-analysis exhibited a significant reduction in the incidence of CMV infection due to antiviral prophylaxis when compared to preemptive therapy: (OR: 6.67, 95% CI: 1.73, 25.66; p = 0.006) for the high-risk group and (OR: 2.74, 95% CI: 1.40, 5.34; p = 0.003) for the intermediate-risk group; however, our meta-analysis failed to find any significant difference in the incidence of CMV infection between the antiviral prophylaxis and preemptive therapy for the all-risks group (OR: 1.18, 95% CI: 0.68, 2.05; p = 0.57). The test for the overall subgroup difference showed moderate heterogeneity (I ^2^) = 72.8%; p = 0.03). The results of the sensitivity analysis found one study (Onor 2013) in the all-risks group contributed to the moderate heterogeneity (**Figure 2**).

**Figure 3:**
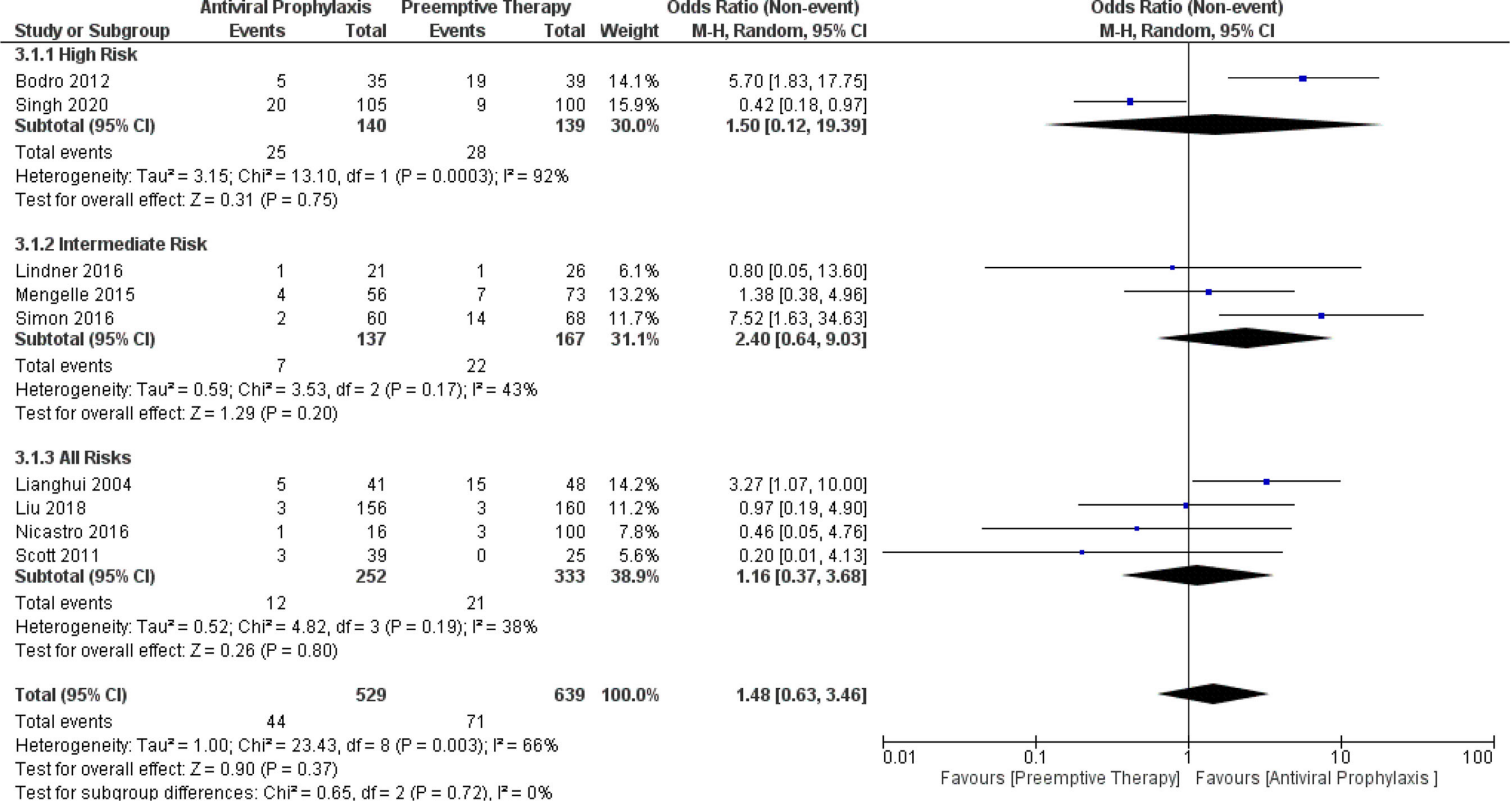
Forest plot depicts a comparison of the incidence of CMV disease among LT recipients undergoing antiviral prophylaxis and preemptive therapy.

In contrast to CMV infection, our meta-analysis demonstrated no significant difference in the incidence of CMV disease between antiviral prophylaxis and preemptive therapy: (OR: 1.50, 95% CI: 0.12, 19.39; p = 0.75) for the high-risk group, (OR: 2.40, 95% CI: 0.64, 9.03; p = 0.20) for the intermediate-risk group, and (OR: 1.16, 95% CI: 0.37, 3.68; p = 0.80) for the all-risks group. The test for the overall subgroup difference displayed no heterogeneity (I ^2^ = 0%; p = 0.72). The result found high heterogeneity between both the studies (Singh 2020 and Bodro 2012) in the high-risk group (**Figure 3**).

### The time to CMV infection and CMV disease

**Figure 4:**
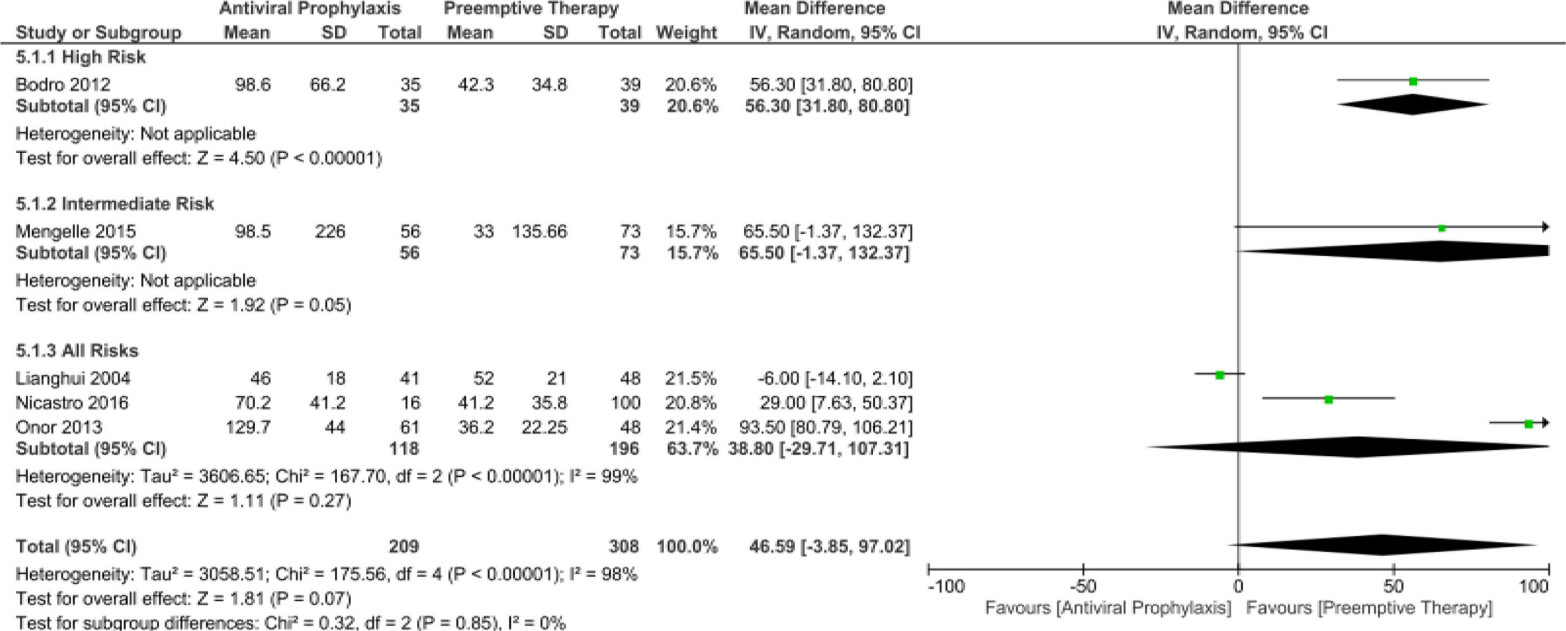
Forest plot depicts a comparison of the time to CMV infection among LT recipients undergoing antiviral prophylaxis and preemptive therapy.

When we compared the time to CMV infection and CMV disease between the antiviral prophylaxis and preemptive therapy for LT recipients, we found the time to CMV infection was significantly longer for antiviral prophylaxis than that of the preemptive therapy in the high-risk group (MD: 56.30, 95% CI: 31.80, 80.80; p < 0.00001). However, there was no significant difference in the time to CMV infection in the intermediate-risk group (MD: 65.50, 95% CI: -1.37, 132.37; p = 0.05) and all-risks group (MD: 38.80, 95% CI: -29.71, 107.31; p = 0.27) between antiviral prophylaxis and preemptive therapy. Yet, according to the trend of the forest plot, the time to CMV infection seems to be longer for antiviral prophylaxis than that of the preemptive therapy. Additionally, we found all the studies in the all-risks group (Lianghui 2004, Nicastro 2016, and Onor 2013) contributed to the significant heterogeneity. The test for the overall subgroup difference showed no heterogeneity (I ^2^ = 0%; p = 0.85) (**Figure 4**).

**Figure 5:**
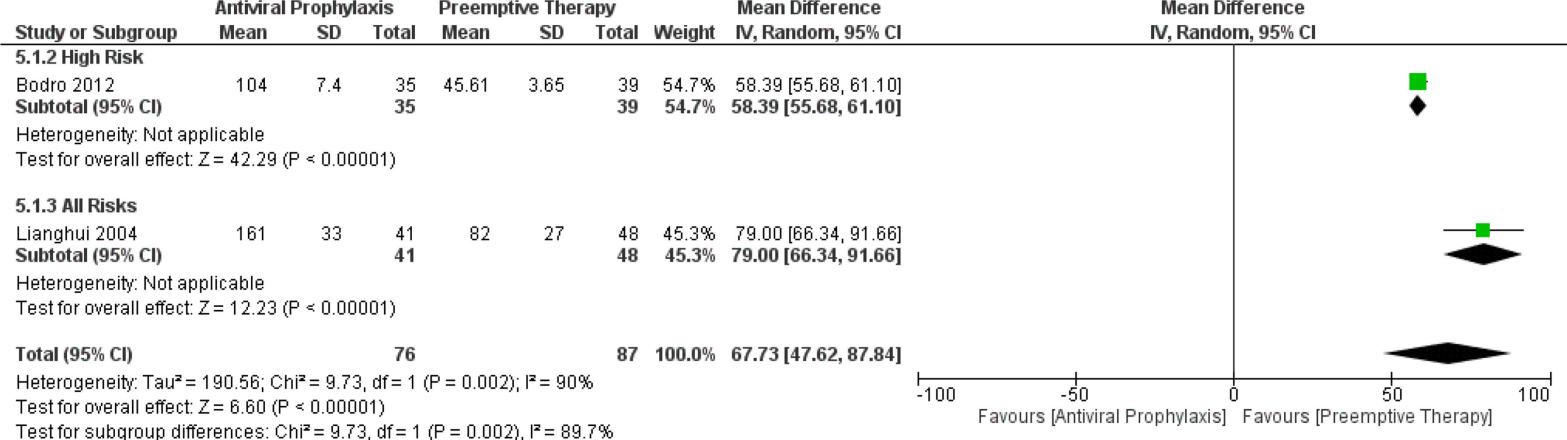
Forest plot depicts a comparison of the time to CMV disease among LT recipients undergoing antiviral prophylaxis and preemptive therapy.

Essentially, our meta-analysis amply confirmed the time to CMV disease was significantly longer for antiviral prophylaxis than that of the preemptive therapy in the high-risk group (MD: 58.39, 95% CI: 55.68, 61.10; p < 0.00001) and for the all-risks group (MD: 79.00, 95% CI: 66.34, 91.66; p < 0.00001). There were no studies comparing the time to CMV disease in the intermediate-risk group between the antiviral prophylaxis and preemptive therapy. The test for the overall subgroup difference displayed high heterogeneity (I ^2^ = 89.7%; p = 0.002) (**Figure 5**).

### Incidence of opportunistic infections

**Figure 6:**
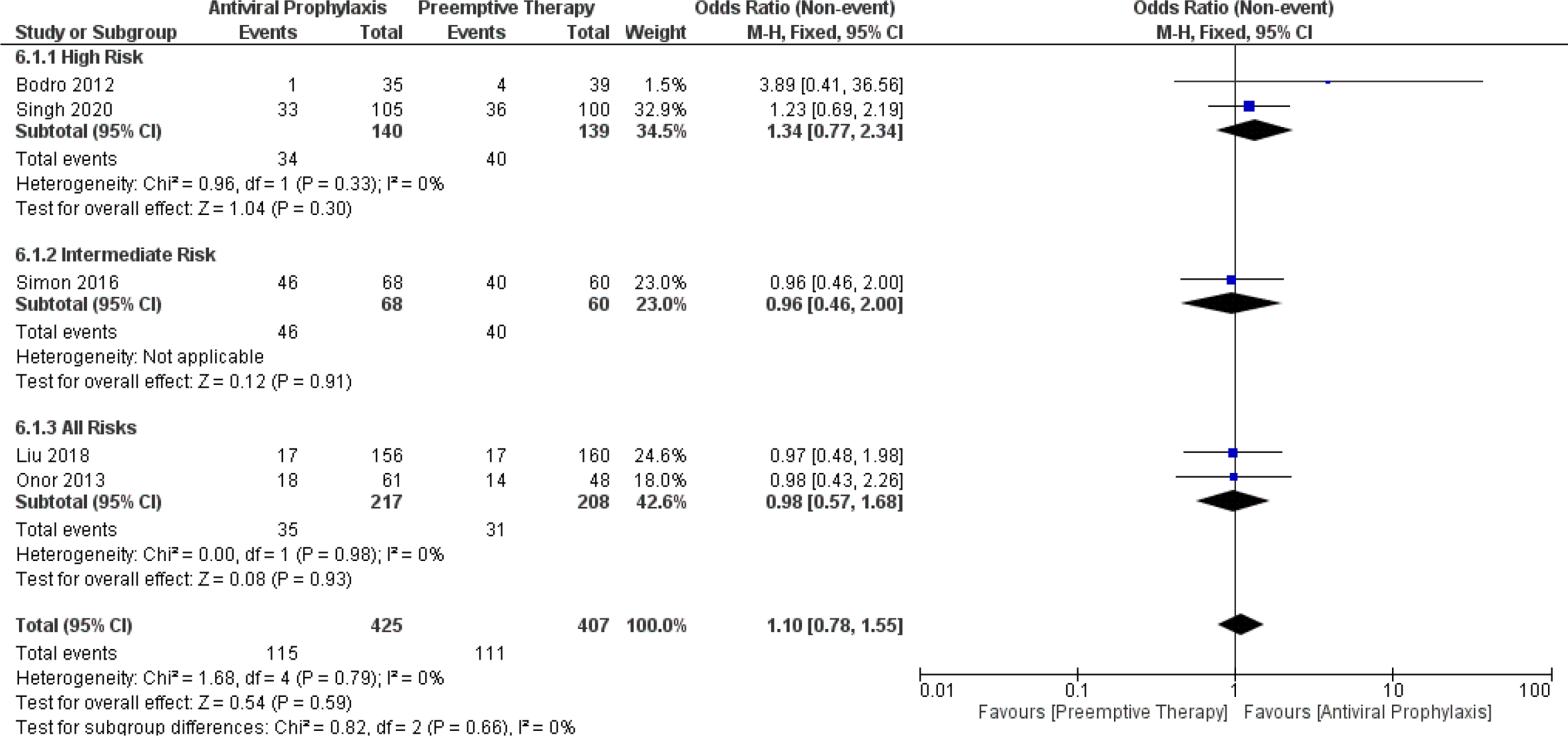
Forest plot depicts a comparison of the incidence of opportunistic infection among LT recipients undergoing antiviral prophylaxis and preemptive therapy.

The pooled estimates of the incidence of opportunistic infections in the overall LT recipients and high-risk group receiving antiviral prophylaxis and preemptive therapy were 27.7% vs. 30.5% (5 vs. 5 studies and 417 vs. 415 patients) and 16.9% vs. 23.1% (2 vs. 2 studies and 140 vs. 139 patients), respectively. (**Supplementary Figure 4**) There was only one study reporting the incidence of opportunistic infections in the intermediate-risk group receiving antiviral prophylaxis (72%) and preemptive therapy (69%) (19). Additionally, we also compared the incidence of opportunistic infections amidst the antiviral prophylaxis and preemptive therapy. Our meta-analysis did not find any significant difference in the incidence of opportunistic infections between either of these strategies: (OR: 1.34, 95% CI: 0.77, 2.34; p = 0.30) for the high-risk group, (OR: 0.96, 95% CI: 0.46, 2.00; p = 0.91) for the intermediate-risk group, and (OR: 0.98, 95% CI: 0.57, 1.68; p = 0.93) for the all-risks group. The test for the overall subgroup difference showed no heterogeneity (I^2^ = 0%; p = 0.66) (**Figure 6**).

### Incidence of acute antibody-mediated rejection

**Figure 7:**
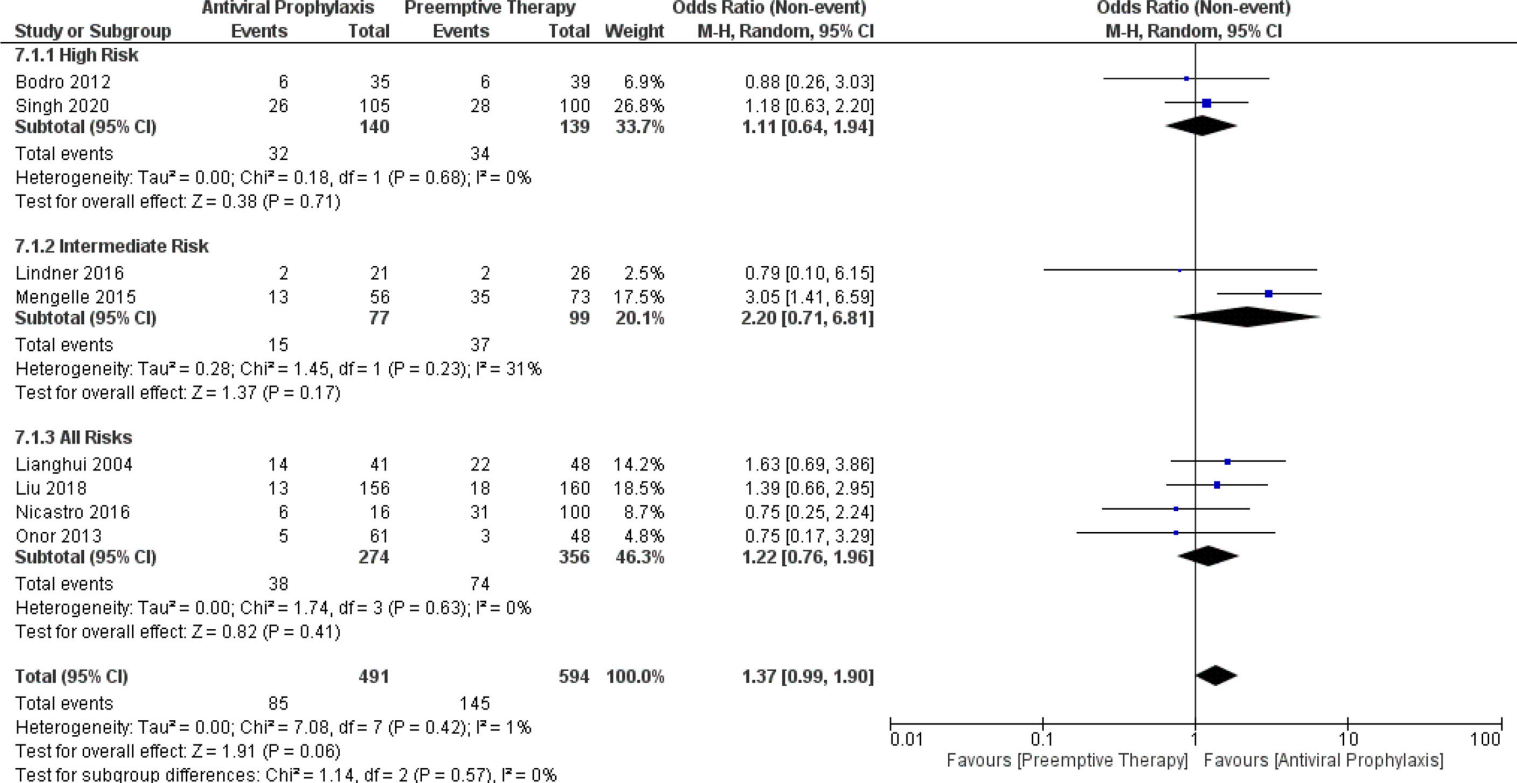
Forest plot depicts a comparison of the incidence of acute antibody-mediated rejection (AMR) among LT recipients undergoing antiviral prophylaxis and preemptive therapy.

The pooled results showed the incidence of aAMR in the overall LT recipients, high-risk group, and intermediate-risk group receiving antiviral prophylaxis and preemptive therapy were 18.2% vs. 23.5% (8 vs. 8 studies and 491 vs. 594 patients), 22.4% vs. 22.2% (2 vs. 2 studies and 140 vs. 139 patients), and 16.7% vs. 27.7% (2 vs. 2 studies and 77 vs. 99 patients), respectively. (**Supplementary Figure 5**) Then, we further compared the incidence of aAMR between antiviral prophylaxis and preemptive therapy. Our meta-analysis found no significant difference in the incidence of aAMR between antiviral prophylaxis and preemptive therapy: (OR: 1.11, 95% CI: 0.64, 1.94; p = 0.71) for the high-risk group, (OR: 2.20, 95% CI: 0.71, 6.81; p = 0.17) for the intermediate-risk group, and (OR: 1.22, 95% CI: 0.76, 1.96; p = 0.41) for the all-risks group. The test for the overall subgroup difference showed no heterogeneity (I^2^ = 0%; p = 0.57) (**Figure 7**).

### Incidence of graft loss

**Figure 8:**
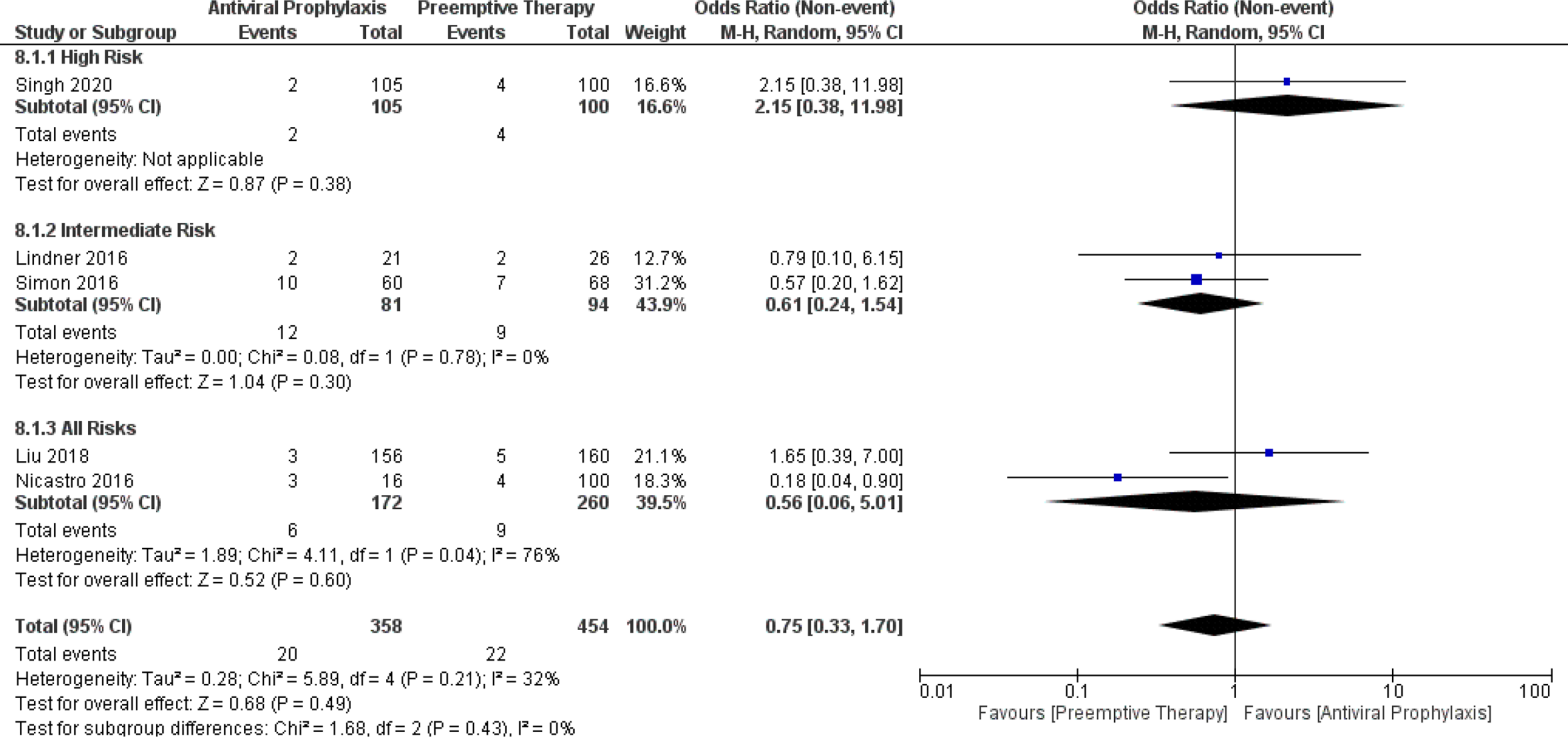
Forest plot depicts a comparison of the incidence of graft loss among LT recipients undergoing antiviral prophylaxis and preemptive therapy.

Similarly, the incidence of graft loss in the overall LT recipients and intermediate-risk group receiving antiviral prophylaxis and preemptive therapy were 5.4% vs. 4.1% (5 vs. 5 studies and 358 vs. 454 patients) and 14.1% vs. 9.4% (2 vs. 2 studies and 81 vs. 94 patients), respectively. (**Supplementary Figure 6**) There was only one study reporting the incidence of graft loss in the high-risk group receiving antiviral prophylaxis (1.9%) and preemptive therapy (4%).9 Besides, the incidence of graft loss between antiviral prophylaxis and preemptive therapy was not significantly different: (OR: 2.15, 95% CI: 0.38, 11.98; p = 0.38) for the high-risk group, (OR: 0.61, 95% CI: 0.24, 1.54; p = 0.30) for the intermediate-risk group, and (OR: 0.56, 95% CI: 0.06, 5.01; p = 0.60) for the all-risks group. We found high heterogeneity among both the studies (Liu 2018 and Nicastro 2016) in the all-risks group. The test for the overall subgroup difference showed no heterogeneity (I^2^ = 0%; p = 0.43) (**Figure 8**).

### Incidence of leukopenia and neutropenia

The pooled results revealed the incidence of leukopenia in the overall LT recipients and intermediate-risk group receiving antiviral prophylaxis and preemptive therapy were 30.5% vs. 25.5% (4 vs. 4 studies and 158 vs. 242 patients) and 20.9% vs. 18.5% (2 vs. 2 studies and 81 vs. 94 patients), respectively. (**Supplementary Figure 7**) Simultaneously, the incidence of neutropenia in the overall LT recipients and intermediate-risk group receiving antiviral prophylaxis and preemptive therapy were 5.8% vs. 6.8% (5 vs. 5 studies and 403 vs. 402 patients and 4% vs. 3.6% (2 vs. 2 studies and 81 vs. 94 patients), respectively (**Supplementary Figure 8**).

**Figure 9:**
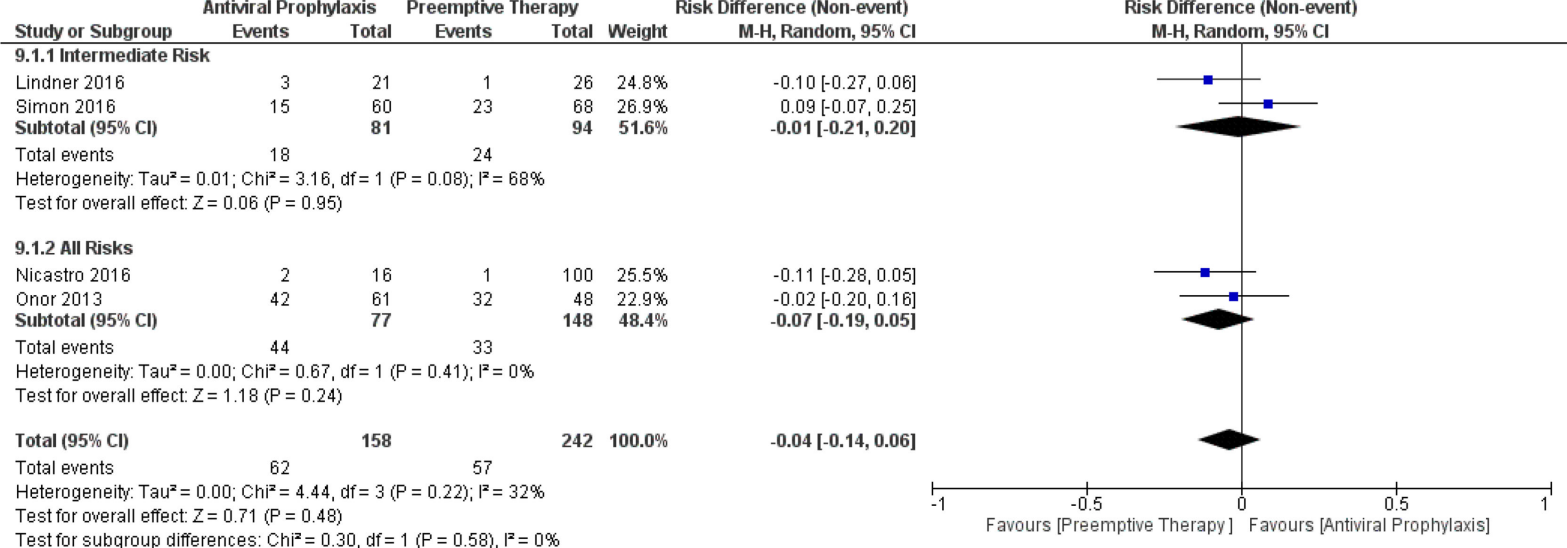
Forest plot depicts a comparison of the incidence of leukopenia among LT recipients undergoing antiviral prophylaxis and preemptive therapy.

**Figure 10:**
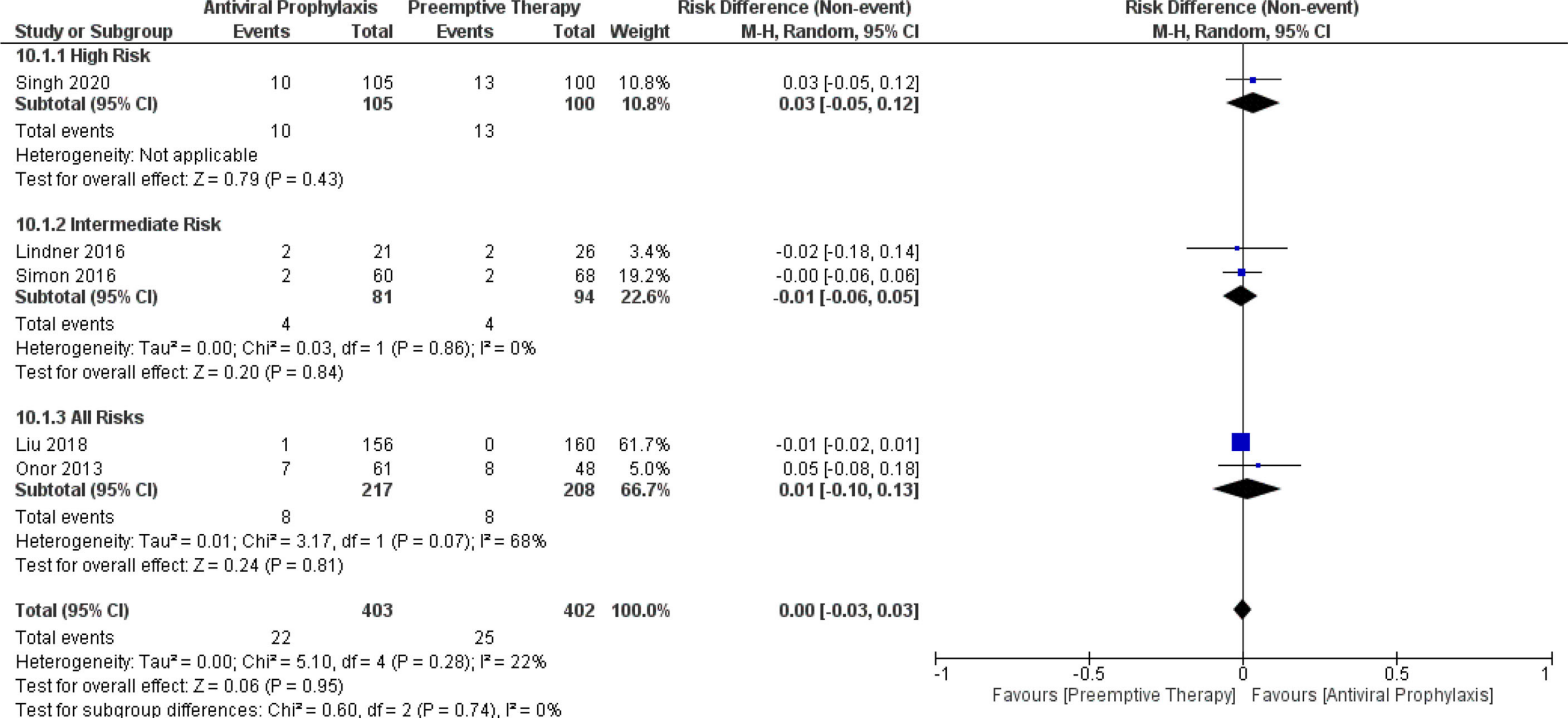
Forest plot depicts a comparison of the incidence of neutropenia among LT recipients undergoing antiviral prophylaxis and preemptive therapy.

When comparing the incidence of leukopenia between antiviral prophylaxis and preemptive therapy, our meta-analysis failed to find any significant difference in the incidence of leukopenia between antiviral prophylaxis and preemptive therapy: (RD: -0.01, 95% CI: -0.21, 0.20; p = 0.95) for the intermediate-risk group and (RD: -0.07, 95% CI: -0.19, 0.05; p = 0.24) for the all-risks group. We found moderate heterogeneity between both the studies (Lindner 2016 and Simon 2016) in the intermediate-risk group. The test for the overall subgroup difference showed no heterogeneity (I^2^ = 0%; p = 0.58). (**Figure 9**) Similarly, our meta-analysis did not find any significant difference in the incidence of neutropenia between antiviral prophylaxis and preemptive therapy: (RD: 0.03, 95% CI: -0.05, 0.12; p = 0.43) for the high-risk group, (RD: -0.01, 95% CI: -0.06, 0.05; p = 0.84) for the intermediate-risk group, and (RD: 0.01, 95% CI: -0.10, 0.13; p = 0.81) for the all-risks group. The result revealed moderate heterogeneity amidst both the studies (Liu 2018 and Onor 2013) in the intermediate-risk group. The test for the overall subgroup difference showed no heterogeneity (I^2^ = 0%; p = 0.74). There were no studies reporting the incidence of leukopenia in the high-risk group receiving antiviral prophylaxis and preemptive therapy (**Figure 10**).

### Incidence of late-onset CMV disease

**Figure 11:**
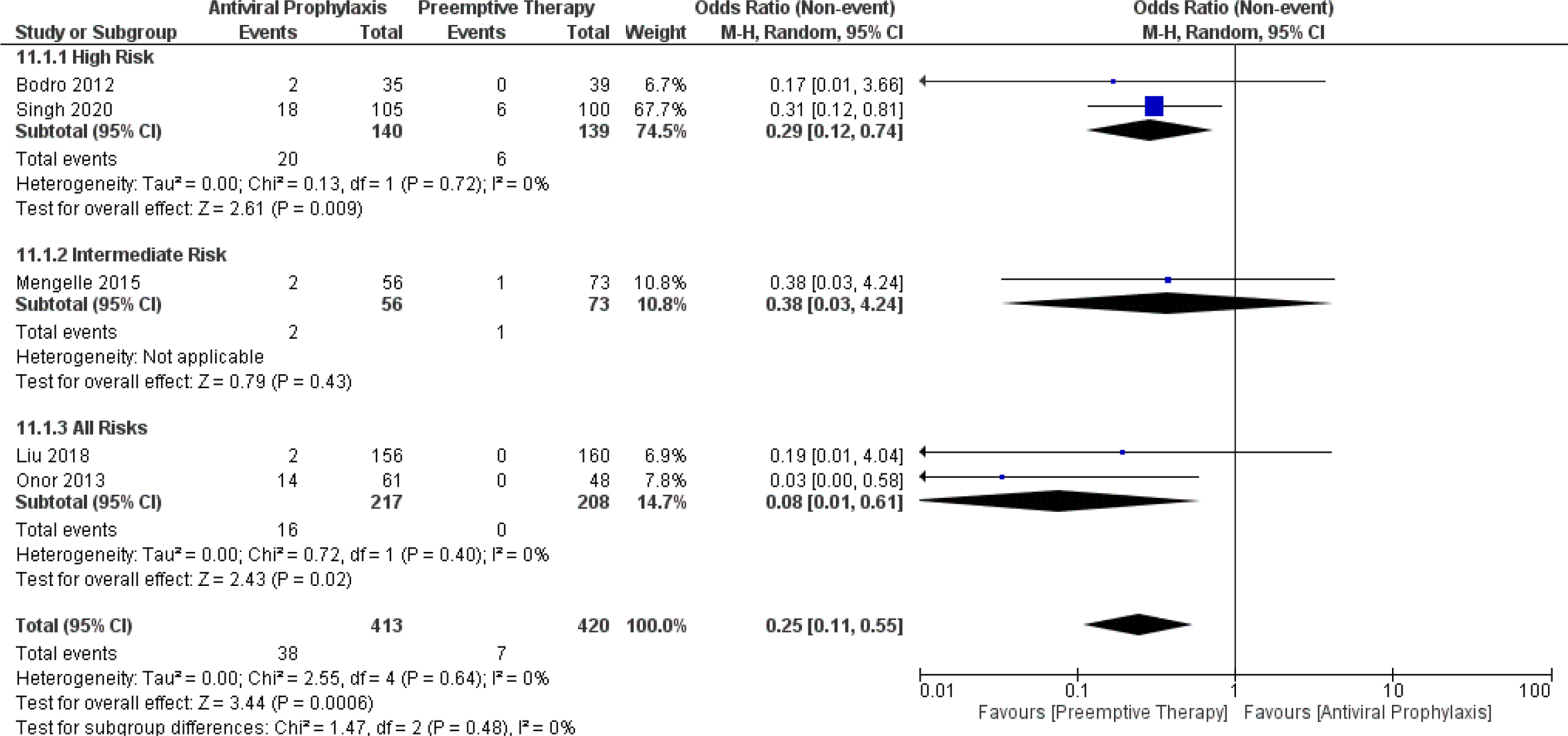
Forest plot depicts a comparison of the incidence of late onset CMV disease among LT recipients undergoing antiviral prophylaxis and preemptive therapy.

From the pooled results, the incidence of late-onset CMV disease in the overall LT recipients, high-risk group, and intermediate-risk group receiving antiviral prophylaxis and preemptive therapy were 7.7% vs. 1% (6 vs. 6 studies and 434 vs. 446 patients), 11.5% vs. 3.4% (2 vs. 2 studies and 140 vs. 139 patients), and 3.1% vs. 1.5% (2 vs. 2 studies and 77 vs. 99 patients), respectively. (**Supplementary Figure 9**) Additionally, we compared the incidence of late-onset CMV disease between the antiviral prophylaxis and preemptive therapy. Our meta-analysis found antiviral prophylaxis was significantly associated with an increased incidence of late-onset CMV disease than that of the preemptive therapy: (OR: 0.29, 95% CI: 0.12, 0.74; p = 0.009) for the high-risk group and (OR: 0.08, 95% CI: 0.01, 0.61; p = 0.02) for the all-risks group. In contrast to the high-risk group and all-risks group, we found no significant difference in the incidence of late-onset CMV disease between the antiviral prophylaxis and preemptive therapy for the intermediate-risk group (OR: 0.38, 95% CI: 0.03, 4.24; p = 0.43). However, from the trend of the forest plot there seems to be a low incidence of late-onset CMV disease in preemptive therapy group compared to that of the antiviral prophylaxis group. The test for the overall subgroup difference showed heterogeneity (I^2^ = 0%; p = 0.48) (**Figure 11**).

### Incidence of all mortality

**Figure 12:**
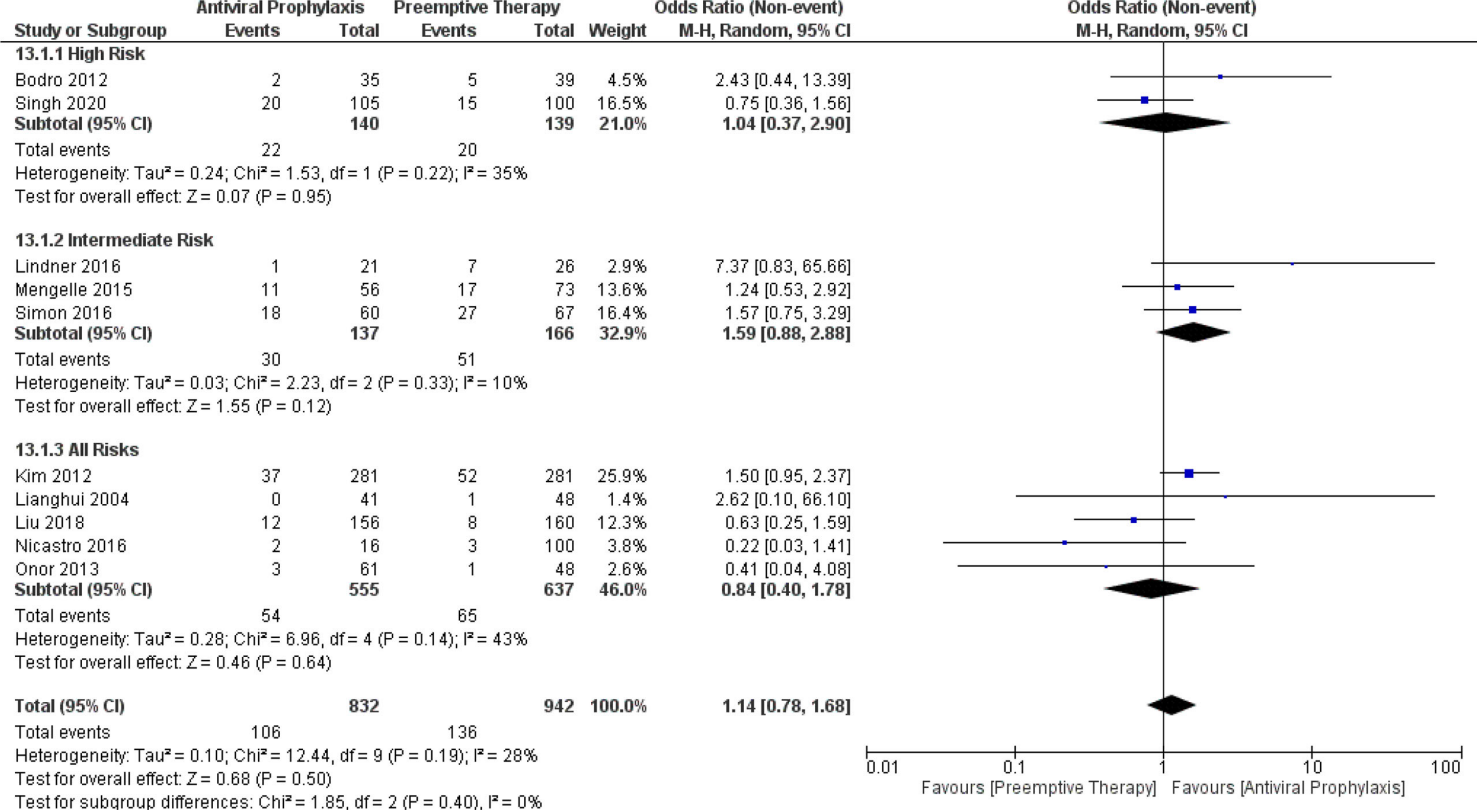
Forest plot depicts a comparison of the incidence of mortality among LT recipients undergoing antiviral prophylaxis and preemptive therapy.

The overall pooled incidence of mortality in the overall LT recipients, high-risk group, and intermediate-risk group receiving antiviral prophylaxis and preemptive therapy was estimated as 10.9% vs. 13.1% (10 vs. 10 studies and 832 vs. 942 patients), 12.4% vs. 14.3% (2 vs. 2 studies and 140 vs. 139 patients), and 17.8% vs. 30.2% (3 vs. 3 studies and 137 vs. 166 patients), respectively. (**Supplementary Figure 10**) Additionally, our meta-analysis failed to find any significant difference in the incidence of mortality between antiviral prophylaxis and preemptive therapy: (OR: 1.04, 95% CI: 0.37, 2.90; p = 0.95) for the high-risk group, (OR: 1.59, 95% CI: 0.88, 2.88; p = 0.12) for the intermediate-risk group, and (OR: 0.84, 95% CI: 0.40, 1.78; p = 0.64) for the all-risks group. The test for the overall subgroup difference showed no heterogeneity (I^2^ = 0%; p = 0.40) (**Figure 12**).

### Incidence of CMV-related mortality

From the pooled results, the incidence of CMV-related mortality in the overall LT recipients receiving antiviral prophylaxis and preemptive therapy was 1% vs. 0.07% (4 vs. 4 studies and 511 vs. 505 patients). (**Supplementary Figure 11**). There was only one study reporting the incidence of CMV-related mortality in the high-risk group receiving antiviral prophylaxis (0%) and preemptive therapy (2.6%) (12). However, there were no studies reporting the incidence of CMV-related mortality in the intermediate-risk group.

**Figure 13:**
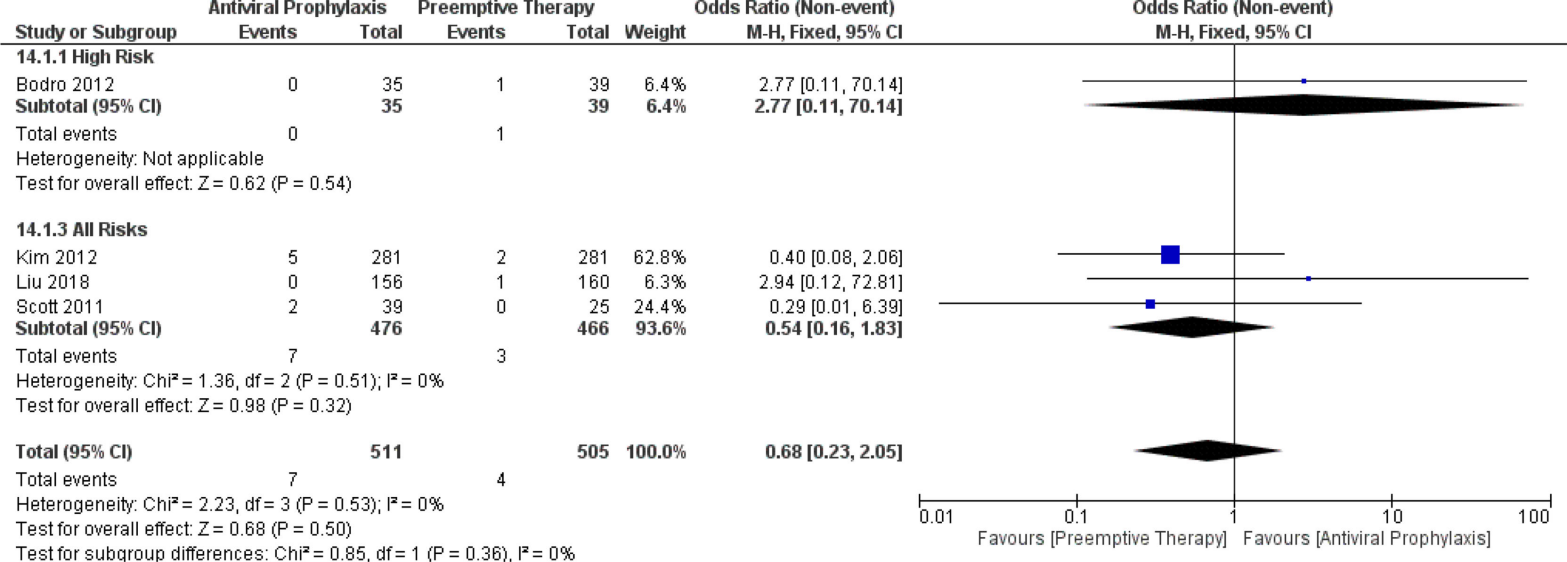
Forest plot depicts a comparison of the incidence of CMV-related mortality among LT recipients undergoing antiviral prophylaxis and preemptive therapy.

Additionally, we further compared the incidence of CMV-related mortality between antiviral prophylaxis and preemptive therapy. Our meta-analysis showed no significant difference in the incidence of CMV-related mortality between antiviral prophylaxis and preemptive therapy: (OR: 2.77, 95% CI: 0.11, 70.14; p = 0.54) for the high-risk group and (OR: 0.54, 95% CI: 0.16, 1.83; p = 0.32) for the all-risks group. The test for the overall subgroup difference showed no heterogeneity (I^2^ = 0%; p = 0.36) (**Figure 13**).

### Development of drug-resistance

The pooled incidence of the incidence of the development of drug-resistance among the overall LT recipients receiving antiviral prophylaxis and preemptive therapy was 3.1% vs. 1.8% (3 vs. 3 studies and 134 vs. 131 patients). (**Supplementary Figure 12**).

Our meta-analysis failed to find any significant difference in the incidence of the development of drug-resistance between antiviral prophylaxis and preemptive therapy: (RD: -0.00, 95% CI: -0.08, 0.07; p = 0.94) for the high-risk group, (RD: -0.00, 95% CI: -0.05, 0.04; p = 0.93) for the intermediate-risk group, and (RD: -0.10, 95% CI: -0.21, 0.01; p = 0.07) for the all-risks group. The test for the overall subgroup difference showed low heterogeneity (I^2^ = 28.8%; p = 0.25) (**Supplementary Figure 12**).

### Development of CMV-specific neutralizing antibodies

There was only one study comparing the proportion of the development of CMV-specific neutralizing antibodies between antiviral prophylaxis and preemptive therapy in the high-risk group of LT recipients, we found the proportion of patients who developed CMV-specific neutralizing antibodies were significantly more in preemptive therapy compared to antiviral prophylaxis (OR: 2.04, 95% CI: 1.03, 4.03.68; p = 0.04) (9). There were no studies reporting the development of CMV-specific neutralizing antibodies in the intermediate-risk group and all-risks group receiving antiviral prophylaxis and preemptive therapy (**Supplementary Figure 13**).

Further, we were also interested in comparing CMV-specific T-cell responses between antiviral prophylaxis and preemptive therapy for LT recipients; however, there were no enough data for this analysis.

## Discussion

This meta-analysis comprehensively compared the efficacy of antiviral prophylaxis and preemptive therapy for the prevention of CMV in LT recipients stratified according to the CMV serostatus. Our study found both the strategies were similarly effective in preventing CMV disease, aAMR, graft loss, mortality (CMV-related mortality and all causes of mortality), and opportunistic infections in LT recipients. Additionally, we found no significant difference in the incidence of leukopenia, neutropenia and the development of drug-resistance between both the strategies. However, antiviral prophylaxis was more effective in controlling CMV infection (in the high-risk and intermediate-risk group), and the mean time to CMV infection (in the high-risk group) and CMV disease was significantly longer in antiviral prophylaxis than that of the preemptive therapy. Whereas, the incidence of late-onset of CMV disease was lower (in the high-risk group and all-risks group) and the proportion of patients developing CMV-specific neutralizing antibodies were significantly higher in preemptive therapy compared to antiviral prophylaxis.

Given that LT recipients routinely receive a high dose of immunosuppressants that traditionally includes steroids in the first 3 months of LT to prevent graft rejection. Technically, immunosuppressants used in LT act as a double-edged sword. Regardless, it typically prevents graft rejection; it also increases the incidence of hypogammaglobulinemia in LT recipients, which is a known risk factor for various opportunistic infection along with CMV in an early post-transplant period (26, 27). In regard to steroids, steroids on one hand can increase CMV load in the blood, on the other hand it also increases the risk of end organ disease due to CMV by reducing the viral load needed to cause the disease (28, 29). From our pooled analysis, the incidence of CMV infection in the overall LT recipients for antiviral prophylaxis and preemptive therapy were 24.7% and 40.4%, respectively. Additionally, our meta-analysis revealed a significant reduction in the incidence of CMV infection in antiviral prophylaxis group when compared to preemptive therapy. Mengelle et al., found that the risk of CMV infection was 4.26 times higher in the patients not receiving antiviral prophylaxis after LT (20). Furthermore, similar results were also obtained by the previous meta-analysis carried out for SOT (30). Therefore, it seems consensus that the use of antiviral prophylaxis, rather than preemptive therapy in the earlier post-transplant period, appears to be justified. Besides, the use of antiviral prophylaxis just after LT also reduces the incidences of other viral infections like Epstein–Barr virus, herpes simplex virus, and respiratory syncytial virus (31). However, antiviral prophylaxis should be avoided in a mannose-binding lectin deficient liver from a donor. Worthley et al., found that the use of antiviral prophylaxis in a mannose-binding lectin deficient liver (hazard ratio, 2.6; p = 0.005) was independently associated with the clinically significant CMV infection, that might have been associated with antiviral prophylaxis-associated neutropenia or enhanced immunosuppression by antiviral prophylaxis (32). Theoretically, these patients might benefit with preemptive therapy, where LT recipients are regularly monitored for any evidence of CMV viremia in an attempt to prevent CMV disease. As yet, this hypothesis must be investigated carefully with a meticulously designed RCT in the future.

Singh et al., in their RCT found that antiviral prophylaxis had a significantly higher incidence of CMV disease compared to preemptive therapy (19% vs 9%) in the high-risk LT recipients (5). In contrast, in a small RCT by Gerna et al., did not find any cases of CMV disease in the either arms (33). Contradicting to these studies, our pooled results showed the incidence of CMV disease in the overall LT recipients for antiviral prophylaxis and preemptive therapy were 6.4% and 9.4%, respectively. However, we observed no significant difference in the incidence of CMV disease between between both the interventions. Not surprisingly, our results are consistent with the other meta-analysis for SOT (30, 34).

In addition, while most of the earlier studies have demonstrated that the mean time to occur CMV infection and CMV disease are longer with the antiviral prophylaxis compared to preemptive therapy for CMV in the LT recipients (10, 12). Consistent with these studies, our study clearly showed the mean time to occur CMV infection (for the high-risk group) and CMV disease was significantly longer in antiviral prophylaxis compared to that of the preemptive therapy. Consequently, it implies that antiviral prophylaxis delays the onset of CMV infection and CMV disease in comparison to preemptive therapy, and should be considered as a beneficial effect of antiviral therapy. However, late-onset of CMV disease after antiviral prophylaxis is considered to be a weakness of antiviral prophylaxis due to its associated mortality (35) Consistent with the earlier studies (9, 11, 22, 30, 36) our study also indicated an association of antiviral prophylaxis with the late-onset of CMV disease. Interestingly, with a careful observation in some studies, preemptive strategy seems to be associated with a higher rate of early-onset of CMV viremic episodes and CMV disease than that of the antiviral prophylaxis (9, 11, 37). Indeed, our meta-analysis found no significant difference in the incidence of mortality (CMV-related mortality and all causes of mortality) for each stratified risk group of the patients between antiviral prophylaxis and preemptive therapy. Earlier there was an interesting observation by Kaminski et al., the authors found that late-onset of CMV disease in the high risk kidney transplant patients was significantly associated with a fewer recurrences, shorter duration of CMV treatment and a faster immune response compared to early-onset of CMV disease (38).

While studies have found that antiviral prophylaxis causes impairment in CD8+ T-cell responses to CMV, because of its complete viral suppression effects. Additionally, they showed that preemptive therapy was associated with the development of a greater CMV-specific immune responses (T-cell responses and neutralizing antibodies) compared to the antiviral prophylaxis (9, 36). Consequently, these studies assumed that antiviral prophylaxis may leads to suboptimal immune response to CMV in LT recipients compared to preemptive therapy.9 From our meta-analysis, the proportion of patients who developed CMV-specific neutralizing antibodies were significantly more in preemptive therapy compared to antiviral prophylaxis. What is less clear is about the quality rather than quantity of CMV-specific immune responses generated by CMV in either of these CMV preventive strategies for LT recipients, and its ability to translate into the clinical benefits. CMV presents various viral proteins that are expressed on the infected cells. Studies have demonstrated that the T-cell response against these proteins is dynamic and polyfunctional, that target against a wide range of CMV proteins (39, 40). However, most of these targets are not an important structural glycoproteins of CMV, but the proteins that help CMV in immune evasion (41). Further, more numbers of studies are definitely needed to understand these complex issues of CMV-specific immune mechanism.

In addition, it is noteworthy that cell-mediated immunity from the CMV seropositive donor or natural immunity itself in the CMV seropositive LT recipient are not effective in preventing a low-level replication of CMV in the absence of antiviral drugs (5, 42). Earlier, a study showed that CMV-specific CD8+ T-cells can be accumulated in SOT recipients without any CMV viremia or disease (39). What become clear that, even at a low viral load, CMV can trigger immune response and systemic inflammation leading to indirect effects like graft rejection, opportunistic infections, graft loss, and atherosclerosis (43, 44). Thus, apparently, the preemptive approach seems to possess some risks in this context. Previously, studies have shown that accelerated atherosclerosis and biopsy-confirmed acute rejection was significantly reduced in the high-risk group of SOT recipients by antiviral prophylaxis (45, 46). Moreover, according to the recent guidelines of the American Society of Transplantation, antiviral prophylaxis is recommended over preemptive therapy for the high-risk SOT recipients (8). Coherent with an earlier meta-analysis of the RCTs for SOT, (22) our study too failed to find any significant differences in graft loss, aAMR, chronic rejection (data not shown), and opportunistic infections between antiviral prophylaxis and preemptive therapy.

Although neither preventive strategy is fully sufficient, taking late-onset of CMV disease and indirect effects of CMV into considerations, other CMV preventive strategies like the hybrid approach (47) long term prophylaxis (48) surveillance of γδ T-cells (49) and timely measurement of plasma IL-10 levels (50) have also been suggested in SOT.

As antiviral drug exposure is higher in the patients receiving antiviral prophylaxis, reasonable concern arises related to the drug related adverse effects (9, 24). However, our study showed no notable differences in the incidence of drug related adverse events (leukopenia, neutopenia and the development of drug-resistance) between antiviral prophylaxis and preemptive therapy. In line with our results, earlier studies also found no significant difference in blood count abnormalities between both strategies (19, 22). Though we couldn’t carry out a meta-analysis of the related costs between antiviral prophylaxis and preemptive therapy, earlier studies have shown conflicting results on the costs for these strategies (51, 52).

## Limitations

Although our meta-analysis includes comparatively high-quality studies, this meta-analysis has numerous limitations. Firstly, a potential publication bias cannot be ruled out as only English language publications were included. Secondly, most of the included articles were retrospective studies which may contribute to some bias in the results. Thirdly, due to short follow-up time and unavailable data in the included studies, we were unable to conduct a meta-analysis of long term indirect effects of CMV in LT recipients (e.g., atherosclerosis, cardiovascular disease, and new-onset diabetes) between both strategies. Lastly, due to a limited available data, we could not execute a cost-effectiveness analysis between these two strategies, as the costs significantly influence the choice of intervention. Nonetheless, our study is still of a great importance and is particularly timely. It directly compared the outcomes of antiviral prophylaxis and preemptive therapy, so that clinicians can choose an appropriate strategy for the prevention of CMV disease in LT recipients.

## Conclusion

In conclusion, we found the use of antiviral prophylaxis, compared with preemptive therapy, is superior in controlling CMV infection and prolonging the time to CMV disease in LT recipients without an increased risk of opportunistic infections, aAMR, graft loss, drug related adverse effects, development of drug resistance, and mortality. We suggest preemptive therapy should be kept as an alternative to antiviral prophylaxis for LT recipients, as recommended by the recent guidelines of the American Society of Transplantation following SOT.

## Data availability statement

The original contributions presented in the study are included in the article/**Supplementary Material**. Further inquiries can be directed to the corresponding authors.

## Author contributions

Concept and design: DY, QL and TL; acquisition and interpretation of data: DY, VA, RY, AS, XH, QZ, PP; drafting of the manuscript: DY, VA, RY; critical revision of the manuscript: DY, QL and TL; final approval: all authors.

## Funding

This study was supported by- National Natural Science Foundation of China (No. 81830089, U20A20378 to TL and 81771713 to QL), National Key Research and Development Program (2019YFC1316000 to TL), Zhejiang Provincial Traditional Chinese Medicine Key Discipline Project (2017-XK-A38 to TL), Zhejiang Provincial Natural Science Foundation of China (2019C03019 to TL and LR18H030001 to QL).

## Conflict of interest

The authors declare that the research was conducted in the absence of any commercial or financial relationships that could be construed as a potential conflict of interest.

## Publisher’s note

All claims expressed in this article are solely those of the authors and do not necessarily represent those of their affiliated organizations, or those of the publisher, the editors and the reviewers. Any product that may be evaluated in this article, or claim that may be made by its manufacturer, is not guaranteed or endorsed by the publisher.
