# Supplementary material for: Antiviral prophylaxis or preemptive therapy for cytomegalovirus after liver transplantation?: A systematic review and meta-analysis
Source: Front Immunol. 2022 Nov 10;13:953210. doi: 10.3389/fimmu.2022.953210 (PMC9685424; doi:10.3389/fimmu.2022.953210)
Supplement: Supplementary file 1 [file DataSheet_1.docx]

**Supplementary table 1: Newcastle-Ottawa quality assessment scale.**

**Supplementary table 2: Pooled estimate of the incidence of events due to CMV in LT recipients receiving antiviral prophylaxis.**

**Supplementary table 3: Pooled estimate of the incidence of events due to CMV in LT recipients undergoing preemptive therapy.**

**Supplementary figure 1: Funnel plots ruling out publication bias.**

**Figure 1.1** Funnel plot of studies depicts a comparison of the incidence of CMV infection among LT recipients undergoing antiviral prophylaxis and preemptive therapy.

**Figure 1.2** Funnel plot of studies depicts a comparison of the incidence of CMV disease among LT recipients undergoing antiviral prophylaxis and preemptive therapy.

**Figure 1.3** Funnel plot of studies depicts a comparison of the time to CMV infection among LT recipients undergoing antiviral prophylaxis and preemptive therapy.

**Figure 1.4** Funnel plot of studies depicts a comparison of the time to CMV disease among LT recipients undergoing antiviral prophylaxis and preemptive therapy.

**Figure 1.5** Funnel plot of studies depicts a comparison of the incidence of opportunistic infection among LT recipients undergoing antiviral prophylaxis and preemptive therapy.

**Figure 1.6** Funnel plot of studies depicts a comparison of the incidence of acute antibody-mediated rejection (AMR) among LT recipients undergoing antiviral prophylaxis and preemptive therapy.

**Figure 1.7** Funnel plot of studies depicts a comparison of the incidence of graft loss among LT recipients undergoing antiviral prophylaxis and preemptive therapy.

**Figure 1.8** Funnel plot of studies depicts a comparison of the incidence of leukopenia among LT recipients undergoing antiviral prophylaxis and preemptive therapy.

**Figure 1.9** Funnel plot of studies depicts a comparison of the incidence of neutropenia among LT recipients undergoing antiviral prophylaxis and preemptive therapy.

**Figure 1.10** Funnel plot of studies depicts a comparison of the incidence of late onset CMV disease among LT recipients undergoing antiviral prophylaxis and preemptive therapy.

**Figure 1.11** Funnel plot of studies depicts a comparison of the incidence of CMV specific neutralizing antibodies among LT recipients undergoing antiviral prophylaxis and preemptive therapy.

**Figure 1.12** Funnel plot of studies depicts a comparison of the incidence of mortality among LT recipients undergoing antiviral prophylaxis and preemptive therapy.

**Figure 1.13** Funnel plot of studies depicts a comparison of the incidence of CMV-related mortality among LT recipients undergoing antiviral prophylaxis and preemptive therapy.

**Figure 1.14** Funnel plot of studies depicts a comparison of the incidence of development of drug-resistance among LT recipients undergoing antiviral prophylaxis and preemptive therapy.
**Figure 1.15** Funnel plot of studies depicts a comparison of the incidence of chronic rejection among LT recipients undergoing antiviral prophylaxis and preemptive therapy.

**Supplemental figure 2: Incidence of CMV infection**

**Supplemental figure 2.1** Incidence of CMV infection in the overall LT recipients undergoing antiviral prophylaxis.

**Supplemental figure 2.2** Incidence of CMV infection in the overall LT recipients undergoing preemptive therapy.

**Supplemental figure 2.3** Incidence of CMV infection in the intermediate-risk group LT recipients undergoing antiviral prophylaxis.

**Supplemental figure 2.4** Incidence of CMV infection in the intermediate-risk group LT recipients undergoing preemptive therapy.

**Supplemental figure 3** Incidence of CMV disease

**Supplemental figure 3.1** Incidence of CMV disease in the overall LT recipients undergoing antiviral prophylaxis.

**Supplemental figure 3.2** Incidence of CMV disease in the overall LT recipients undergoing preemptive therapy.

**Supplemental figure 3.3** Incidence of CMV disease in the high-risk group LT recipients undergoing antiviral prophylaxis.

**Supplemental figure 3.4** Incidence of CMV disease in the high-risk group LT recipients undergoing preemptive therapy.

**Supplemental figure 3.5** Incidence of CMV disease in the intermediate-risk group LT recipients undergoing antiviral prophylaxis.

**Supplemental figure 3.6** Incidence of CMV disease in the intermediate-risk group LT recipients undergoing preemptive therapy.

**Supplemental figure 4: Incidence of opportunistic infection**

**Supplemental figure 4.1** Incidence of opportunistic infection in the overall LT recipients undergoing antiviral prophylaxis.

**Supplemental figure 4.2** Incidence of opportunistic infection in the overall LT recipients undergoing preemptive therapy.

**Supplemental figure 4.3** Incidence of opportunistic infection in the high-risk group LT recipients undergoing antiviral prophylaxis.

**Supplemental figure 4.4** Incidence of opportunistic infection in the high-risk group LT recipients undergoing preemptive therapy.

**Supplemental figure 5:** **Incidence of acute antibody-mediated rejection (AMR)**

**Supplemental figure 5.1** Incidence of acute antibody-mediated rejection (AMR) in the overall LT recipients undergoing antiviral prophylaxis.

**Supplemental figure 5.2** Incidence of acute antibody-mediated rejection (AMR) in the overall LT recipients undergoing preemptive therapy.

**Supplemental figure 5.3** Incidence of acute antibody-mediated rejection (AMR) in the high-risk group LT recipients undergoing antiviral prophylaxis.

**Supplemental figure 5.4** Incidence of acute antibody-mediated rejection (AMR) in the high-risk group LT recipients undergoing preemptive therapy.

**Supplemental figure 5.5** Incidence of acute antibody-mediated rejection (AMR) in the intermediate-risk group LT recipients undergoing antiviral prophylaxis.

**Supplemental figure 5.6** Incidence of acute antibody-mediated rejection (AMR) in the intermediate-risk group LT recipients undergoing preemptive therapy.

**Supplemental figure 6: Incidence of graft loss**

**Supplemental figure 6.1** Incidence of graft loss in the overall LT recipients undergoing antiviral prophylaxis.

**Supplemental figure 6.2** Incidence of graft loss in the overall LT recipients undergoing preemptive therapy.

**Supplemental figure 6.3** Incidence of graft loss in the intermediate-risk group LT recipients undergoing antiviral prophylaxis.

**Supplemental figure 6.4** Incidence of graft loss in the intermediate-risk group LT recipients undergoing preemptive therapy.

**Supplemental figure 7: Incidence of leukopenia**

**Supplemental figure 7.1** Incidence of leukopenia in the overall LT recipients undergoing antiviral prophylaxis.

**Supplemental figure 7.2** Incidence of leukopenia in the overall LT recipients undergoing preemptive therapy.

**Supplemental figure 7.3** Incidence of leukopenia in the intermediate-risk group LT recipients undergoing antiviral prophylaxis.

**Supplemental figure 7.4** Incidence of leukopenia in the intermediate-risk group LT recipients undergoing preemptive therapy.

**Supplemental figure 8:** **Incidence of neutropenia**

**Supplemental figure 8.1** Incidence of neutropenia in the overall LT recipients undergoing antiviral prophylaxis.

**Supplemental figure 8.2** Incidence of neutropenia in the overall LT recipients undergoing preemptive therapy.

**Supplemental figure 8.3** Incidence of neutropenia in the intermediate-risk group LT recipients undergoing antiviral prophylaxis.

**Supplemental figure 8.4** Incidence of neutropenia in the intermediate-risk group LT recipients undergoing preemptive therapy.

**Supplemental figure 9:** **Incidence of late-onset CMV disease**

**Supplemental figure 9.1** Incidence of late-onset CMV disease in the overall LT recipients undergoing antiviral prophylaxis.

**Supplemental figure 9.2** Incidence of late-onset CMV disease in the overall LT recipients undergoing preemptive therapy.

**Supplemental figure 9.3** Incidence of late-onset CMV disease in the high-risk group LT recipients undergoing antiviral prophylaxis.

**Supplemental figure 9.4** Incidence of late-onset CMV disease in the high-risk group LT recipients undergoing preemptive therapy.

**Supplemental figure 9.5** Incidence of late-onset CMV disease in the intermediate-risk group LT recipients undergoing antiviral prophylaxis.

**Supplemental figure 9.6** Incidence of late-onset CMV disease in the intermediate-risk group LT recipients undergoing preemptive therapy.

**Supplemental figure 10:** **Incidence of mortality**

**Supplemental figure 10.1** Incidence of mortality in the overall LT recipients undergoing antiviral prophylaxis.

**Supplemental figure 10.2** Incidence of mortality in the overall LT recipients undergoing preemptive therapy.

**Supplemental figure 10.3** Incidence of mortality in the high-risk group LT recipients undergoing antiviral prophylaxis.

**Supplemental figure 10.4** Incidence of mortality in the high-risk group LT recipients undergoing preemptive therapy.

**Supplemental figure 10.5** Incidence of mortality in the intermediate-risk group LT recipients undergoing antiviral prophylaxis.

**Supplemental figure 10.6** Incidence of mortality in the intermediate-risk group LT recipients undergoing preemptive therapy.

**Supplemental figure 11: Incidence of CMV related mortality**

**Supplemental figure 11.1** Incidence of CMV related mortality in the overall LT recipients undergoing antiviral prophylaxis.

**Supplemental figure 11.2** Incidence of CMV related mortality in the overall LT recipients undergoing preemptive therapy.

**Supplemental figure 12:** **Incidence of the development of drug resistance**

**Supplemental figure 12.1** Incidence of the development of drug resistance in the overall LT recipients undergoing antiviral prophylaxis.

**Supplemental figure 12.2** Incidence of the development of drug resistance in the overall LT recipients undergoing preemptive therapy.

**Supplemental figure 13**

**Supplemental figure 13.1** Forest plot depicts a comparison of the incidence of CMV specific neutralizing antibodies among LT recipients undergoing antiviral prophylaxis and preemptive therapy.

**Supplemental figure 13.2** Forest plot depicts a comparison of the incidence of the development of drug resistance among LT recipients undergoing antiviral prophylaxis and preemptive therapy.

**Supplemental figure 13.3** Forest plot depicts a comparison of the incidence of chronic rejection among LT recipients undergoing antiviral prophylaxis and preemptive therapy.

**Supplementary table 1: Newcastle-Ottawa quality assessment scale.**

|  | *Selection* | | | | *Comparability* | *Outcome* | | |  |
| --- | --- | --- | --- | --- | --- | --- | --- | --- | --- |
| **Author, country, year** | *Representativeness of Exposed Cohort* | *Selection of Non exposed* | *Ascertainment of Exposure* | *Outcome Not Present at Start*  *Of study* | *Comparability of cohorts* | *Assessment of Outcome* | *Adequate Follow-Up Length* | *Adequacy of Follow-Up* | *the overall* |
| Singh *et al*, USA, 2020 | 1 | 1 | 1 | 1 | 2 | 1 | 1 | 1 | 9 |
| Liu *et al*, USA, 2018 | 1 | 1 | 1 | 1 | 2 | 1 | 1 | 1 | 9 |
| Bodro *et al*, Spain, 2012 | 1 | 1 | 1 | 1 | 2 | 1 | 1 | 1 | 9 |
| Simon *et al*, Germany, 2016 | 1 | 1 | 1 | 1 | 2 | 1 | 1 | 1 | 9 |
| Mengelle *et al*, France, 2015 | 1 | 1 | 1 | 1 | 1 | 1 | 1 | 1 | 8 |
| Scott *et al*, Australia, 2011 | 1 | 1 | 1 | 1 | 1 | 0 | 1 | 1 | 7 |
| Onor *et al*, USA, 2013 | 1 | 1 | 1 | 1 | 1 | 1 | 0 | 1 | 7 |
| Lindner *et al*, Germany, 2016 | 1 | 1 | 1 | 1 | 2 | 1 | 1 | 1 | 9 |
| Nicastro *et al*, Italy, 2016 | 1 | 1 | 1 | 1 | 1 | 1 | 1 | 1 | 8 |
| Kim *et al*, Korea, 2012 | 1 | 1 | 1 | 1 | 2 | 1 | 1 | 1 | 9 |
| Lianghui *et al*, China, 2004 | 1 | 1 | 1 | 1 | 2 | 1 | 1 | 1 | 9 |

**Supplementary table 2: Pooled estimate of the incidence of events due to CMV in LT recipients receiving antiviral prophylaxis.**

| Outcome | Groups | No. of studies | No. of events  (n) | Total Patients  (n) | Incidence/Prevalence | I^2^ | OR (CI) | p |
| --- | --- | --- | --- | --- | --- | --- | --- | --- |
| CMV Infection | Overall  LT recipients | 9 | 129 | 610 | 24.7% | 93.63% | 0.247(0.142-0.352) | <0.001 |
|  | Intermediate-risk | 3 | 20 | 137 | 11.9% | 91.05% | 0.119 (-0.034-0.271) | <0.001 |
| CMV Disease | Overall  LT recipients | 10 | 44 | 590 | 6.4% | 72.06% | 0.064 (0.031-0.096) | <0.001 |
|  | High-risk | 2 | 25 | 140 | 17.6% | 0% | 0.176 (0.113-0.239) | 0.499 |
|  | Intermediate-risk | 3 | 7 | 137 | 4.6% | 0% | 0.046 (0.011-0.080) | 0.655 |
| Opportunistic infections | Overall  LT recipients | 5 | 109 | 417 | 27.7% | 96.44% | 0.277 (0.099-0.455) | <0.001 |
|  | High-risk | 2 | 34 | 140 | 16.9% | 96.51% | 0.169 (-0.111-0.449) | <0.001 |
| Acute antibody-mediated rejection (AMR) | Overall  LT recipients | 8 | 85 | 491 | 18.2% | 76.87% | 0.182 (0.111-0.253) | <0.001 |
|  | High-risk | 2 | 32 | 140 | 22.4% | 0% | 0.224 (0.156-0.293) | 0.318 |
|  | Intermediate-risk | 2 | 15 | 77 | 16.7% | 61.12% | 0.167 (0.033-0.301) | 0.109 |
| Graft loss | Overall  LT recipients | 5 | 20 | 358 | 5.4% | 69.48% | 0.054 (0.011-0.097) | 0.011 |
|  | Intermediate-risk | 2 | 12 | 81 | 14.1% | 0% | 0.141 (0.066-0.216) | 0.373 |
| Leukopenia | Overall  LT recipients | 4 | 62 | 158 | 30.5% | 94.06% | 0.305 (0.035-0.575) | <0.001 |
|  | Intermediate-risk | 2 | 18 | 81 | 20.9% | 21.98% | 0.209 (0.107-0.311) | 0.258 |
| Neutropenia | the overall  LT recipients | 5 | 22 | 403 | 5.8% | 77.56% | 0.058 (0.010-0.105) | 0.001 |
|  | Intermediate-risk | 2 | 4 | 81 | 4% | 0% | 0.040 (-0.002-0.083) | 0.363 |
| Late-onset CMV disease | Overall  LT recipients | 6 | 38 | 434 | 7.7% | 84.72% | 0.077 (0.021-0.133) | <0.001 |
|  | High-risk | 2 | 20 | 140 | 11.5% | 77.86% | 0.115 (0.003-0.227) | 0.034 |
|  | Intermediate-risk | 2 | 2 | 77 | 3.1% | 0% | 0.031 (-0.008-0.069) | 0.747 |
| Mortality | Overall  LT recipients | 10 | 106 | 832 | 10.9% | 82.84% | 0.109 (0.061-0.156) | <0.001 |
|  | High-risk | 2 | 22 | 140 | 12.4% | 83.08% | 0.124 (-0.007-0.255) | 0.015 |
|  | Intermediate-risk | 3 | 30 | 137 | 17.8% | 83.22% | 0.178 (0.032-0.324) | 0.003 |
| CMV-related mortality | Overall  LT recipients | 4 | 7 | 511 | 1% | 29.71% | 0.010 (-0.001-0.002) | 0.234 |
| Development of drug resistance | Overall LT recipients | 3 | 6 | 134 | 3.1% | 84% | 0.031 (-0.005-0.067) | 0.245 |

**Supplementary table 3: Pooled estimate of the incidence of events due to CMV in LT recipients undergoing preemptive therapy.**

| Outcome | Groups | No. of studies | No. of events  (n) | Total Patients  (n) | Incidence/Prevalence | I^2^ | OR (CI) | p |
| --- | --- | --- | --- | --- | --- | --- | --- | --- |
| CMV Infection | Overall  LT recipients | 10 | 331 | 868 | 40.4% | 98.03% | 0.404 (0.225-0.583) | <0.001 |
|  | Intermediate-risk | 3 | 47 | 167 | 23.8% | 97.48% | 0.238 (-0.090-0.566) | <0.001 |
| CMV Disease | Overall  LT recipients | 10 | 71 | 687 | 9.4% | 87.8% | 0.094 (0.050-0.139) | <0.001 |
|  | High-risk | 2 | 28 | 139 | 28.2% | 95.42% | 0.282 (-0.107-0.671) | <0.001 |
|  | Intermediate-risk | 3 | 22 | 167 | 10.8% | 72.74% | 0.108 (0.022-0.195) | 0.026 |
| Opportunistic infections | Overall LT recipients | 5 | 117 | 415 | 30.5% | 96.08% | 0.305 (0.106-0.504) | <0.001 |
|  | High-risk | 2 | 40 | 139 | 23.1% | 92.96% | 0.231 (-0.021-0.484) | <0.001 |
| Acute antibody-mediated rejection (AMR) | Overall LT recipients | 8 | 145 | 594 | 23.5% | 90.88% | 0.235 (0.133-0.337) | <0.001 |
|  | High risk | 2 | 34 | 139 | 22.2% | 66.36% | 0.222 (0.099-0.345) | 0.085 |
|  | Intermediate-risk | 2 | 37 | 99 | 27.7% | 96.2% | 0.277 (-0.117-0.672) | <0.001 |
| Graft loss | Overall LT recipients | 5 | 22 | 454 | 4.1% | 0% | 0.041 (0.023-0.059) | 0.433 |
|  | Intermediate-risk | 2 | 9 | 94 | 9.4% | 0% | 0.094 (0.035-0.153) | 0.684 |
| Leukopenia | Overall LT recipients | 4 | 57 | 242 | 25.5% | 97.51% | 0.255 (0.027-0.484) | <0.001 |
|  | Intermediate-risk | 2 | 24 | 94 | 18.5% | 94.75% | 0.185 (-0.108-0.479) | <0.001 |
| Neutropenia | Overall LT recipients | 5 | 25 | 402 | 6.8% | 84.58% | 0.068 (0.012-0.124) | <0.001 |
|  | Intermediate-risk | 2 | 4 | 94 | 3.6% | 0% | 0.036 (-0.002-0.073) | 0.397 |
| Late-onset CMV disease | Overall LT recipients | 6 | 7 | 446 | 1% | 21.25% | 0.010 (-0.001-0.022) | 0.274 |
|  | High-risk | 2 | 6 | 139 | 3.4% | 61.33% | 0.034 (-0.013-0.080) | 0.108 |
|  | Intermediate-risk | 2 | 1 | 99 | 1.5% | 0% | 0.015 (-0.009-0.038) | 0.869 |
| Mortality | Overall LT recipients | 10 | 136 | 942 | 13.1% | 90.62% | 0.131 (0.074-0.188) | <0.001 |
|  | High-risk | 2 | 20 | 139 | 14.3% | 14.3% | 0.143 (0.085-0.202) | 0.735 |
|  | Intermediate-risk | 3 | 51 | 166 | 30.2% | 59.27% | 0.302 (0.189-0.415) | 0.086 |
| CMV-related mortality | Overall LT recipients | 4 | 4 | 505 | 0.7% | 0% | 0.007 (-0.000-0.015) | 0.862 |
| Development of drug resistance | Overall LT recipients | 3 | 2 | 131 | 1.8% | 0% | 0.018 (-0.005-0.041) | 0.934 |

**Supplementary figure 1: Funnel plots ruling out publication bias.**

**Figure 1.1** Funnel plot of studies depicts a comparison of the incidence of CMV infection among LT recipients undergoing antiviral prophylaxis and preemptive therapy.

| 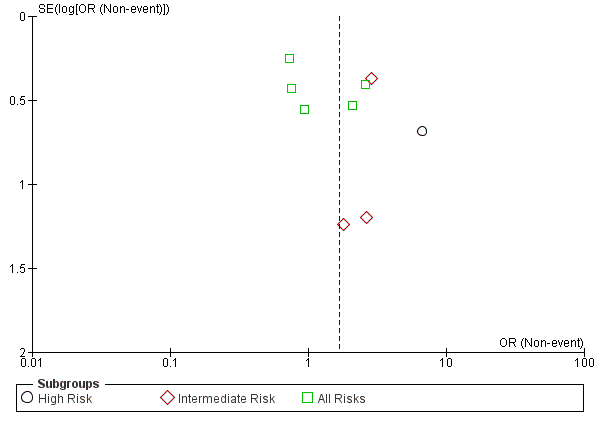 |
| --- |

**Figure 1.2** Funnel plot of studies depicts a comparison of the incidence of CMV disease among LT recipients undergoing antiviral prophylaxis and preemptive therapy.

| 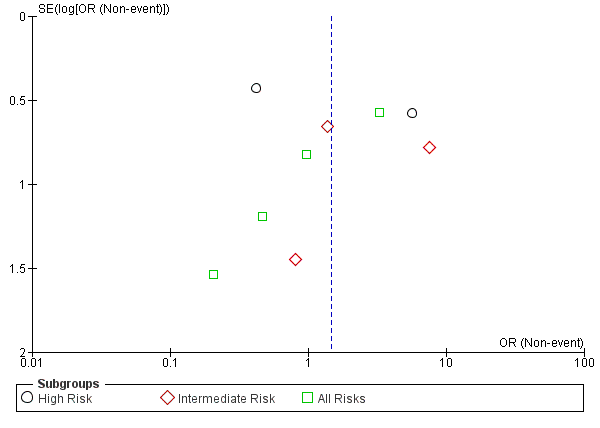 |
| --- |

**Figure 1.3** Funnel plot of studies depicts a comparison of the time to CMV infection among LT recipients undergoing antiviral prophylaxis and preemptive therapy.

| 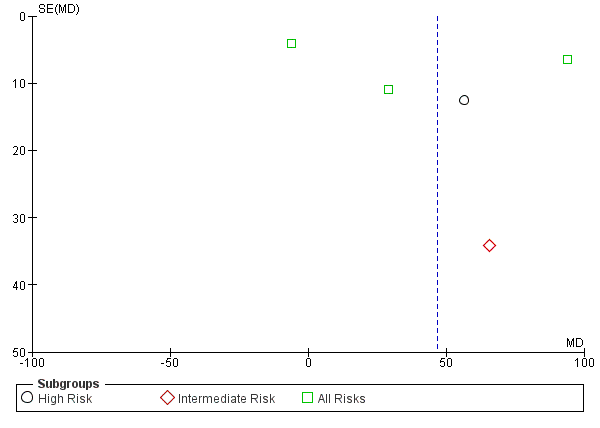 |
| --- |

**Figure 1.4** Funnel plot of studies depicts a comparison of the time to CMV disease among LT recipients undergoing antiviral prophylaxis and preemptive therapy.

| **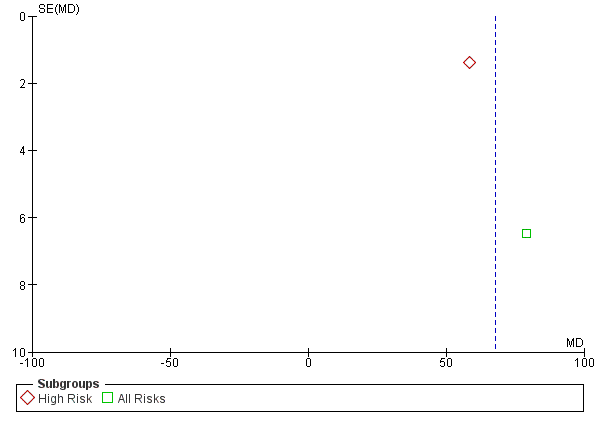** |
| --- |

**Figure 1.5** Funnel plot of studies depicts a comparison of the incidence of opportunistic infection among LT recipients undergoing antiviral prophylaxis and preemptive therapy.

| 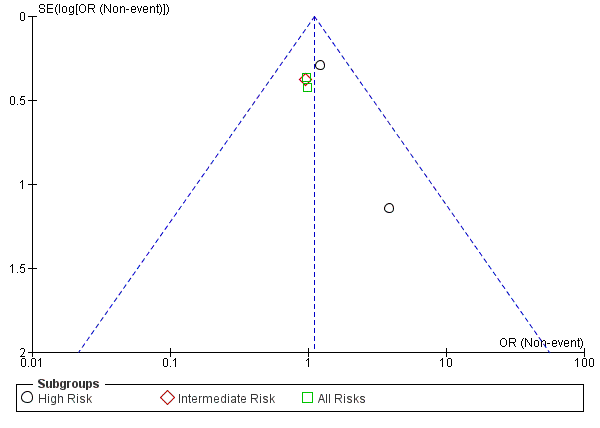 |
| --- |

**Figure 1.6** Funnel plot of studies depicts a comparison of the incidence of acute antibody-mediated rejection (AMR) among LT recipients undergoing antiviral prophylaxis and preemptive therapy.

| 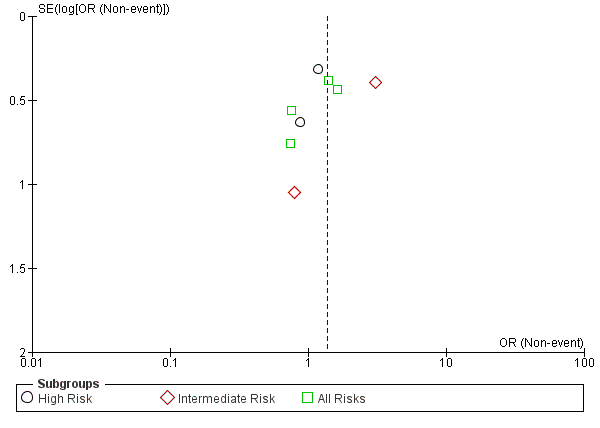 |
| --- |

**Figure 1.7** Funnel plot of studies depicts a comparison of the incidence of graft loss among LT recipients undergoing antiviral prophylaxis and preemptive therapy.

| 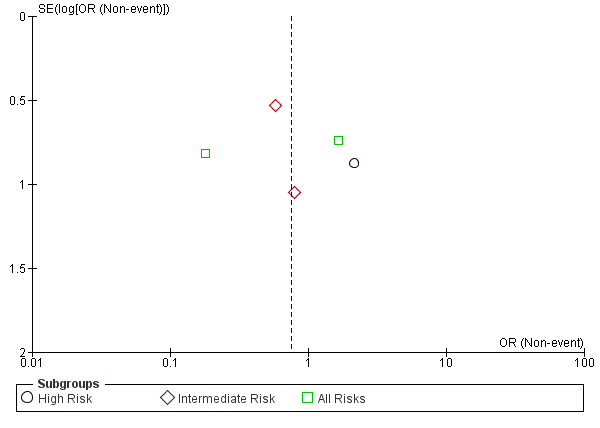 |
| --- |

**Figure 1.8** Funnel plot of studies depicts a comparison of the incidence of leukopenia among LT recipients undergoing antiviral prophylaxis and preemptive therapy.

| 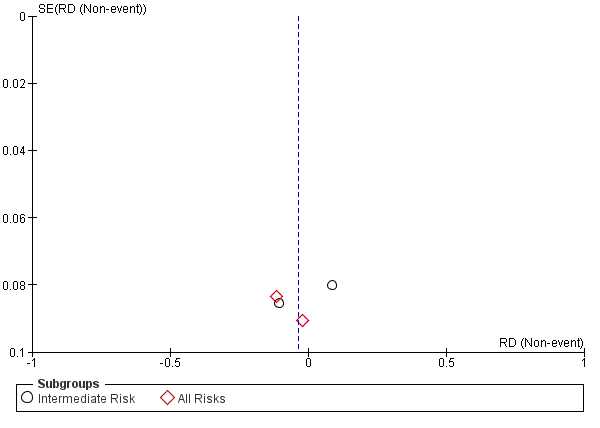 |
| --- |

**Figure 1.9** Funnel plot of studies depicts a comparison of the incidence of neutropenia among LT recipients undergoing antiviral prophylaxis and preemptive therapy.

| 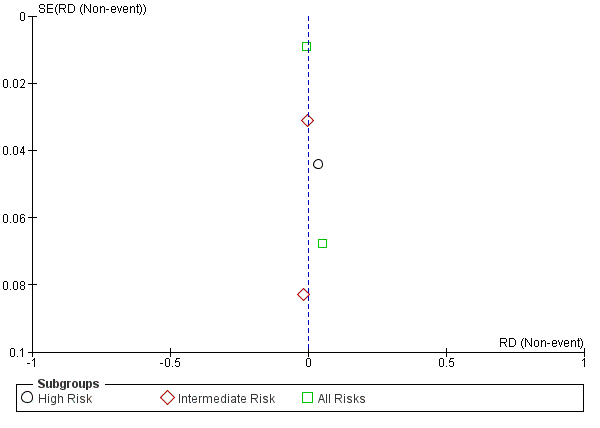 |
| --- |

**Figure 1.10** Funnel plot of studies depicts a comparison of the incidence of late onset CMV disease among LT recipients undergoing antiviral prophylaxis and preemptive therapy.

| 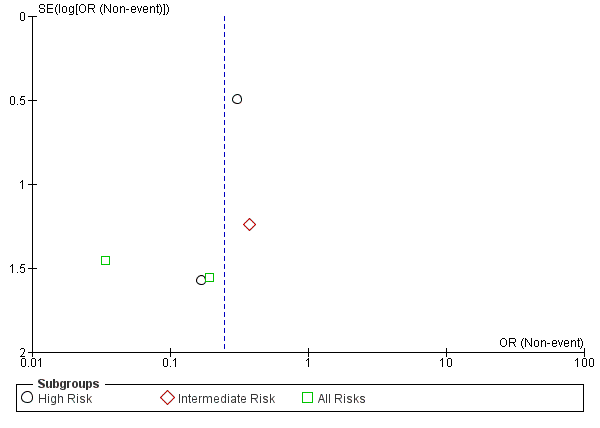 |
| --- |

**Figure 1.11** Funnel plot of studies depicts a comparison of the incidence of CMV specific neutralizing antibodies among LT recipients undergoing antiviral prophylaxis and preemptive therapy.

| 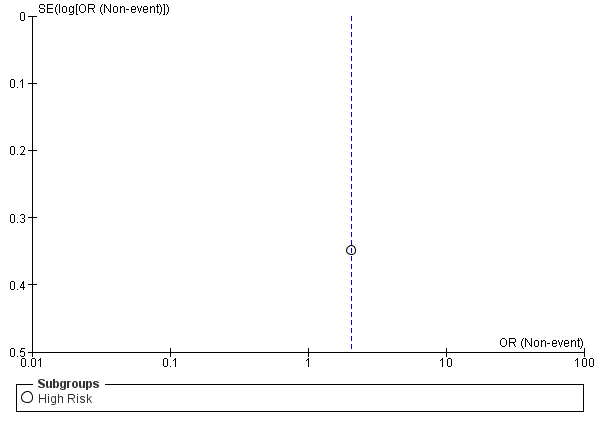 |
| --- |

**Figure 1.12** Funnel plot of studies depicts a comparison of the incidence of mortality among LT recipients undergoing antiviral prophylaxis and preemptive therapy.

| 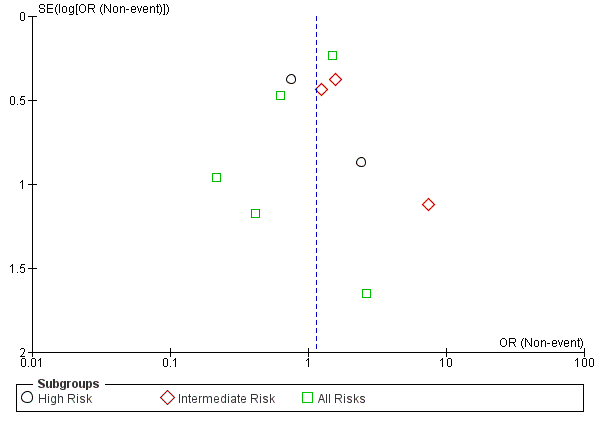 |
| --- |

**Figure 1.13** Funnel plot of studies depicts a comparison of the incidence of CMV-related mortality among LT recipients undergoing antiviral prophylaxis and preemptive therapy.

| 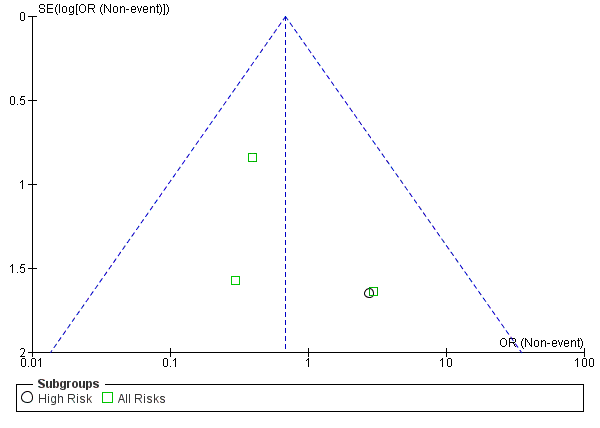 |
| --- |

**Figure 1.14** Funnel plot of studies depicts a comparison of the incidence of development of drug-resistance among LT recipients undergoing antiviral prophylaxis and preemptive therapy.

| 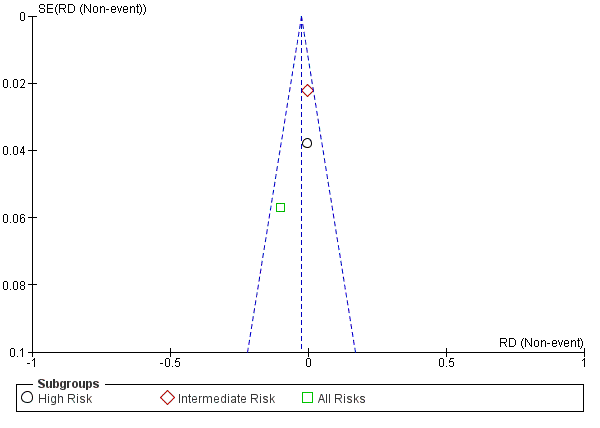 |
| --- |

**Figure 1.15** Funnel plot of studies depicts a comparison of the incidence of chronic rejection among LT recipients undergoing antiviral prophylaxis and preemptive therapy.

| **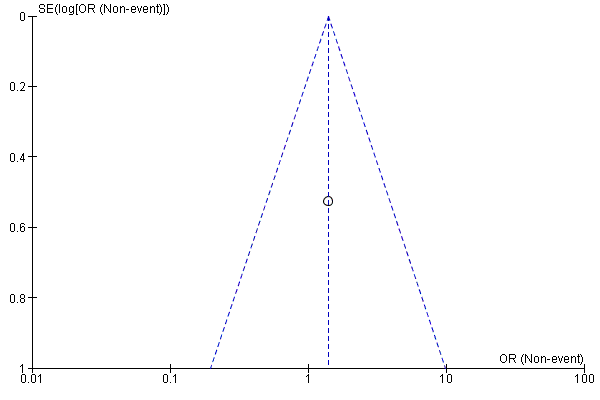** |
| --- |

**Supplemental figure 2: Incidence of CMV infection**

**Supplemental figure 2.1** Incidence of CMV infection in the overall LT recipients undergoing antiviral prophylaxis.

| 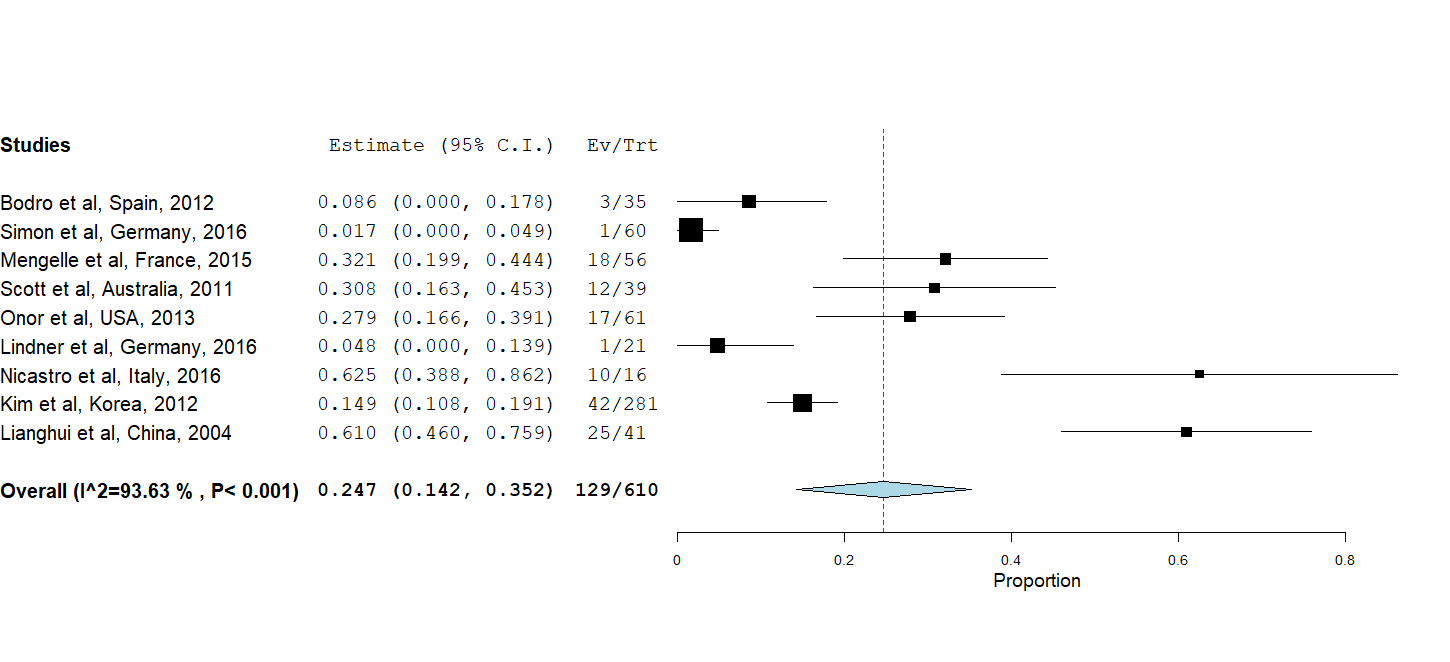 |
| --- |

**Supplemental figure 2.2** Incidence of CMV infection in the overall LT recipients undergoing preemptive therapy.

| 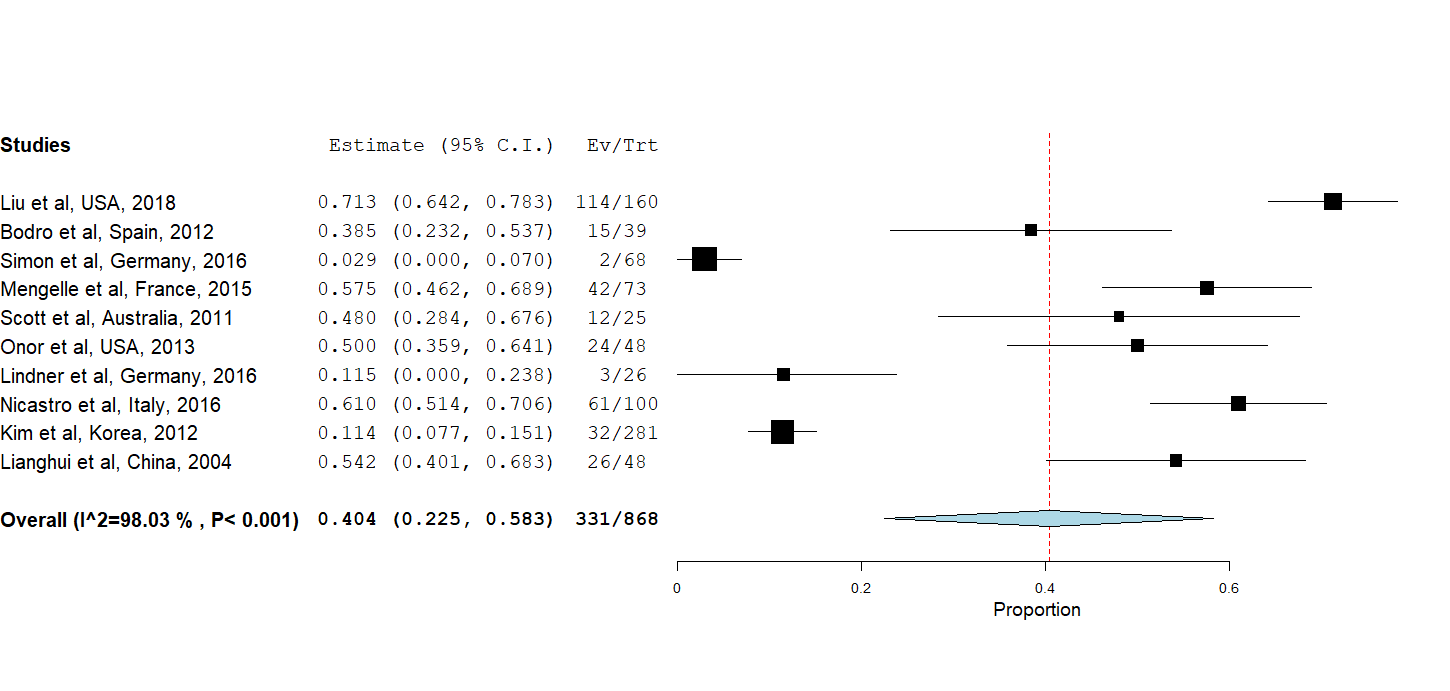 |
| --- |

**Supplemental figure 2.3** Incidence of CMV infection in the intermediate-risk group LT recipients undergoing antiviral prophylaxis.

| 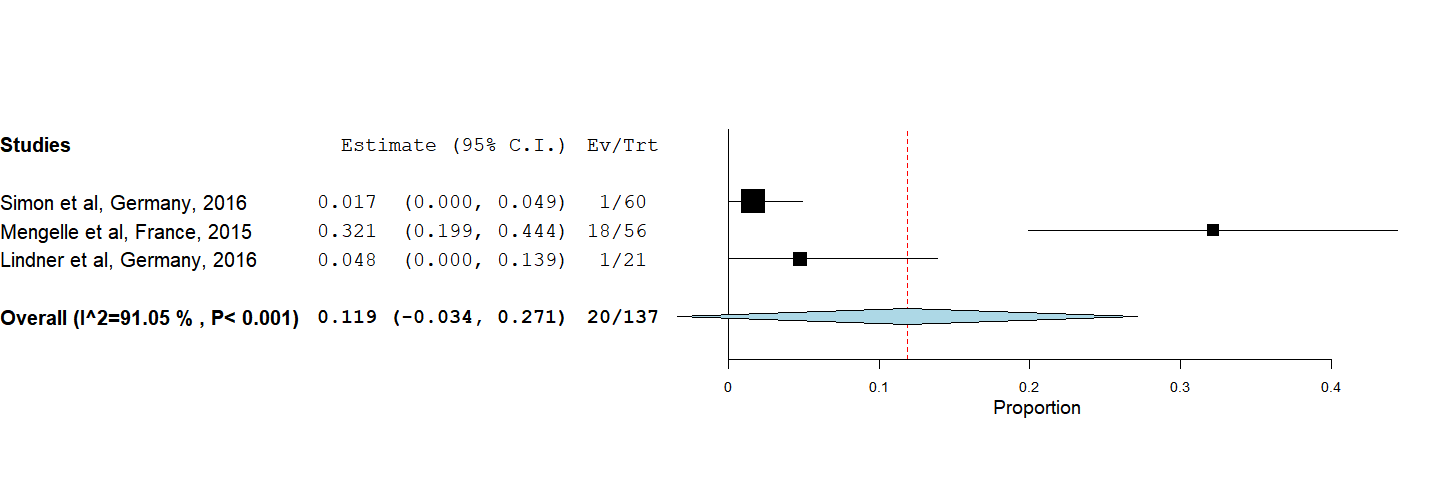 |
| --- |

**Supplemental figure 2.4** Incidence of CMV infection in the intermediate-risk group LT recipients undergoing preemptive therapy.

| 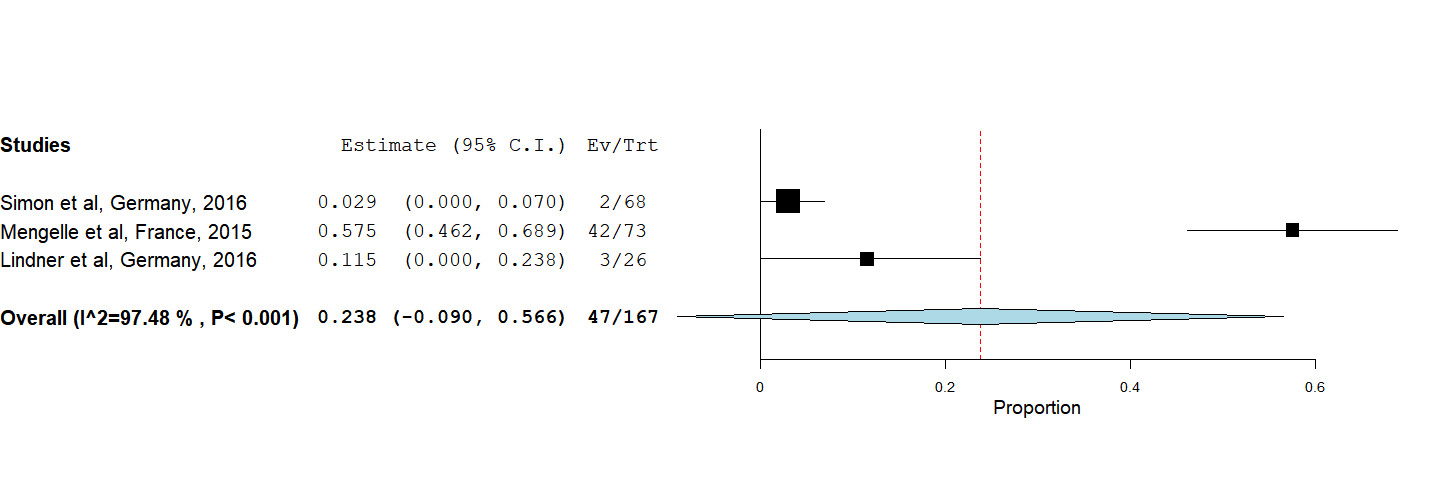 |
| --- |

**Supplemental figure 3** Incidence of CMV disease

**Supplemental figure 3.1** Incidence of CMV disease in the overall LT recipients undergoing antiviral prophylaxis.

| 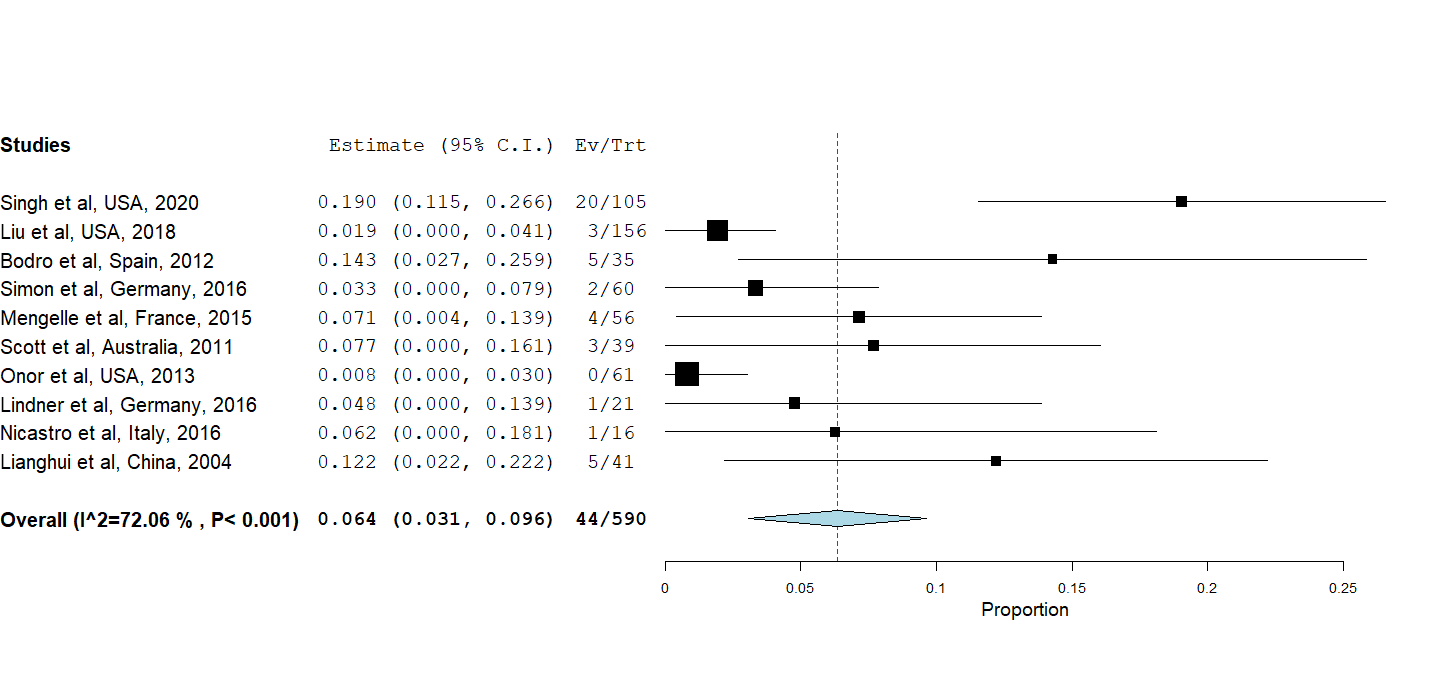 |
| --- |

**Supplemental figure 3.2** Incidence of CMV disease in the overall LT recipients undergoing preemptive therapy.

| 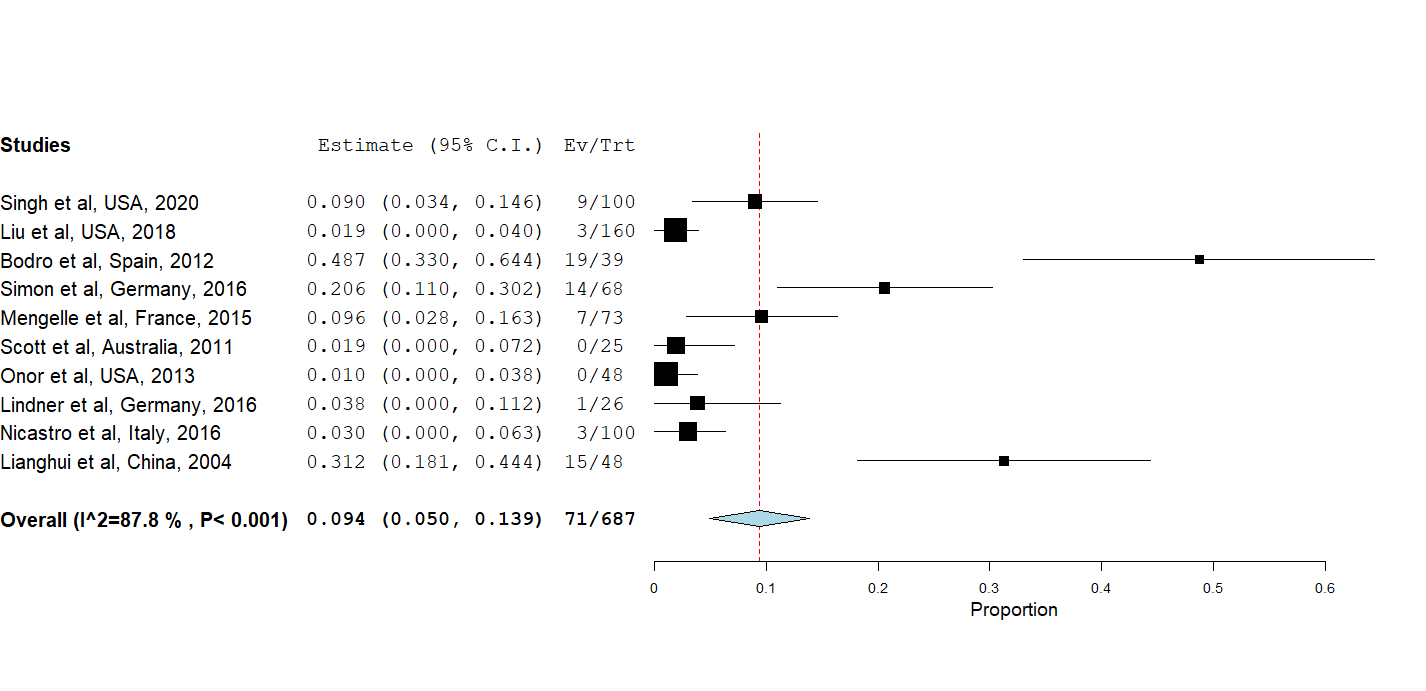 |
| --- |

**Supplemental figure 3.3** Incidence of CMV disease in the high-risk group LT recipients undergoing antiviral prophylaxis.

| 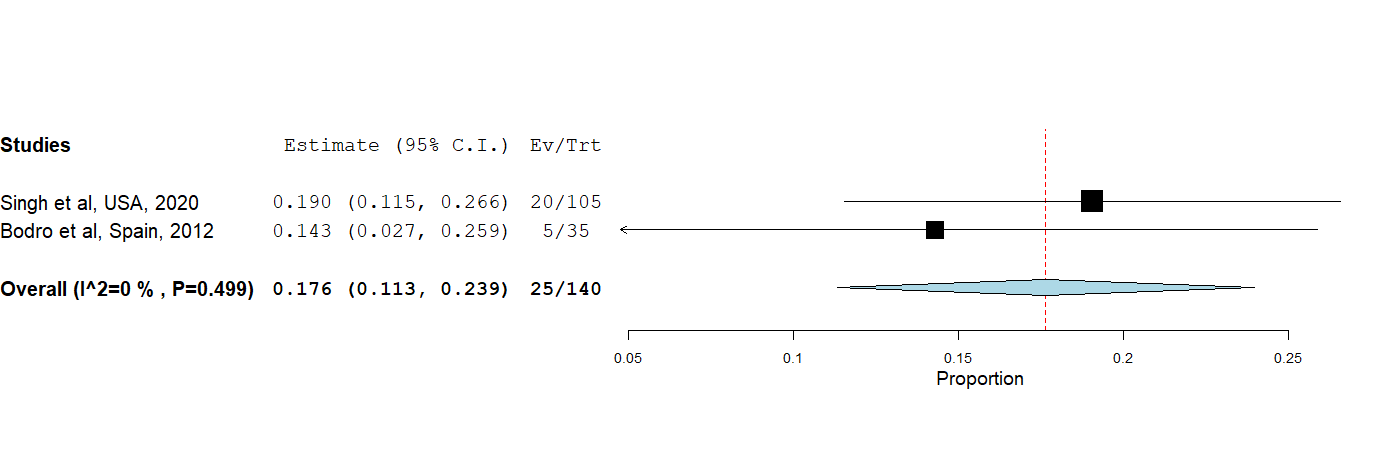 |
| --- |

**Supplemental figure 3.4** Incidence of CMV disease in the high-risk group LT recipients undergoing preemptive therapy.

| 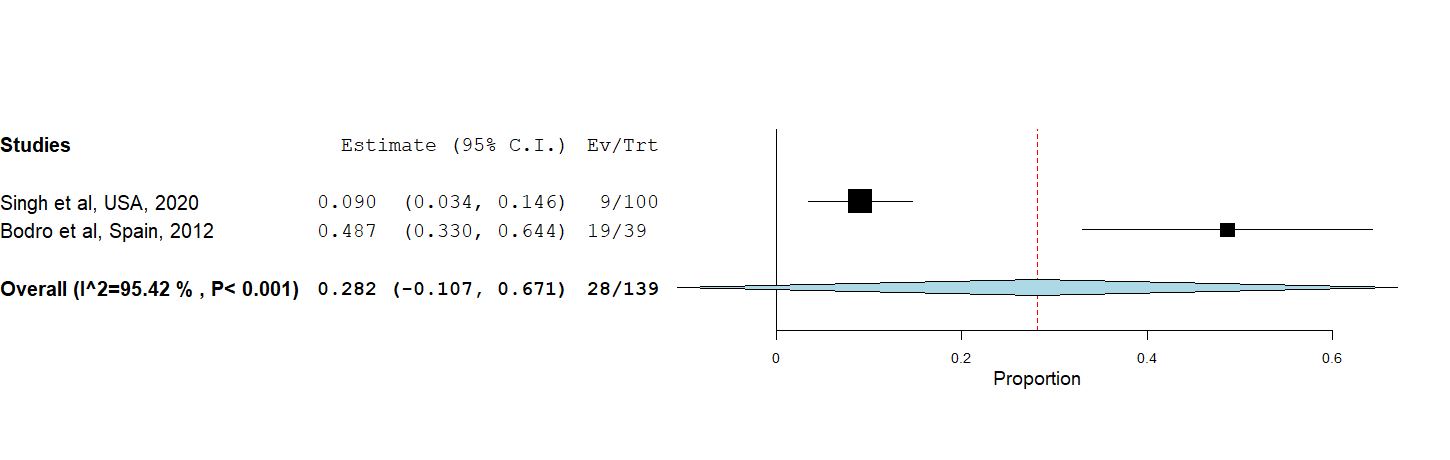 |
| --- |

**Supplemental figure 3.5** Incidence of CMV disease in the intermediate-risk group LT recipients undergoing antiviral prophylaxis.

| 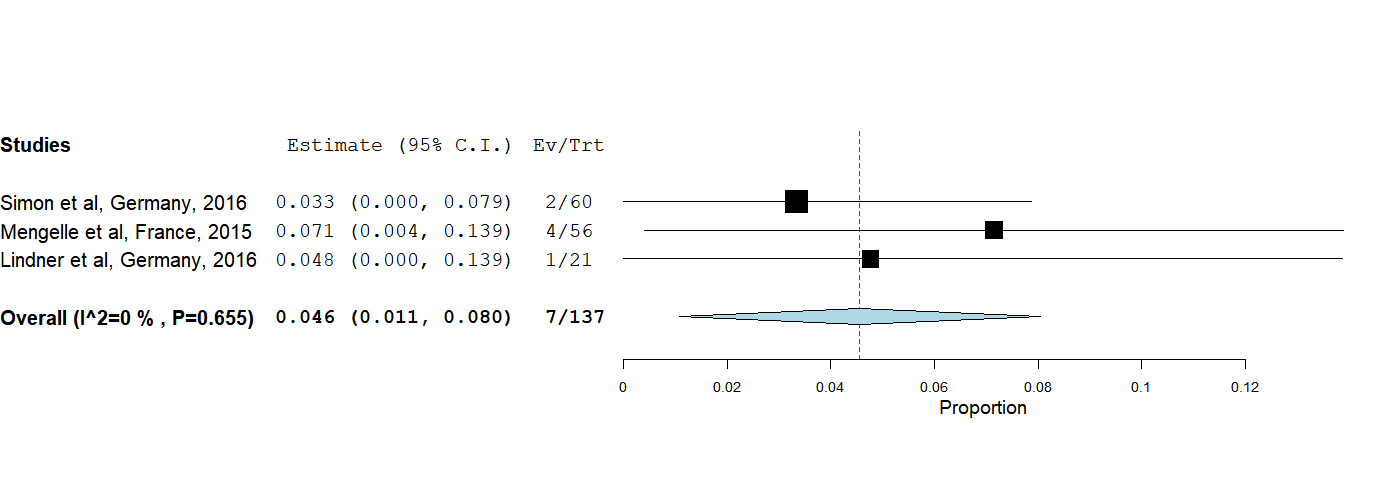 |
| --- |

**Supplemental figure 3.6** Incidence of CMV disease in the intermediate-risk group LT recipients undergoing preemptive therapy.

| 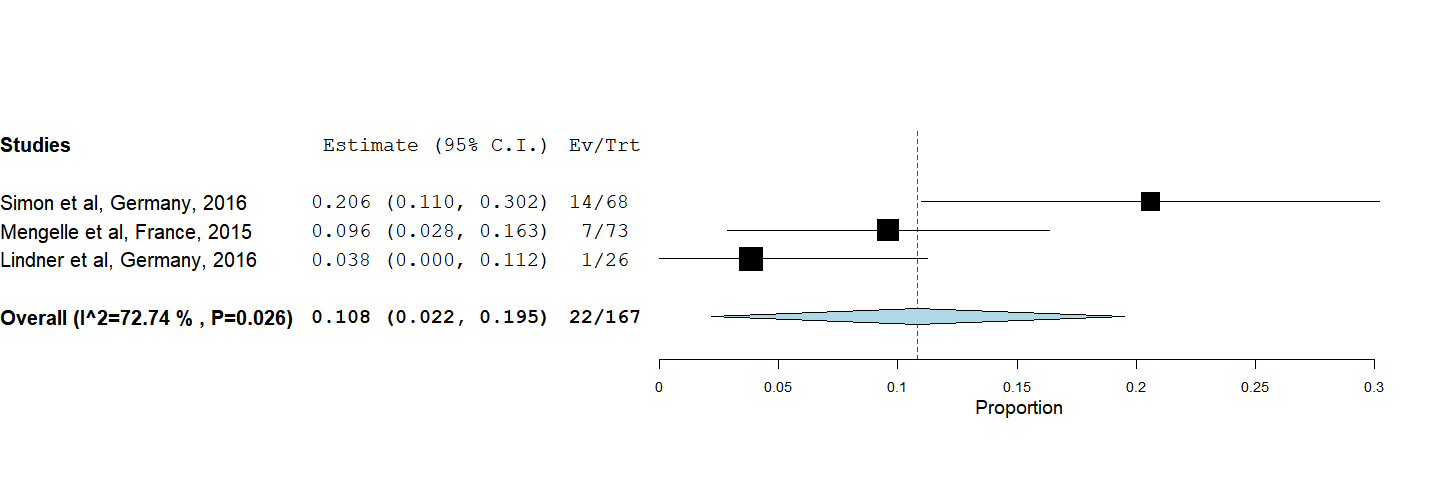 |
| --- |

**Supplemental figure 4:** **Incidence of opportunistic infection**

**Supplemental figure 4.1** Incidence of opportunistic infection in the overall LT recipients undergoing antiviral prophylaxis.

| 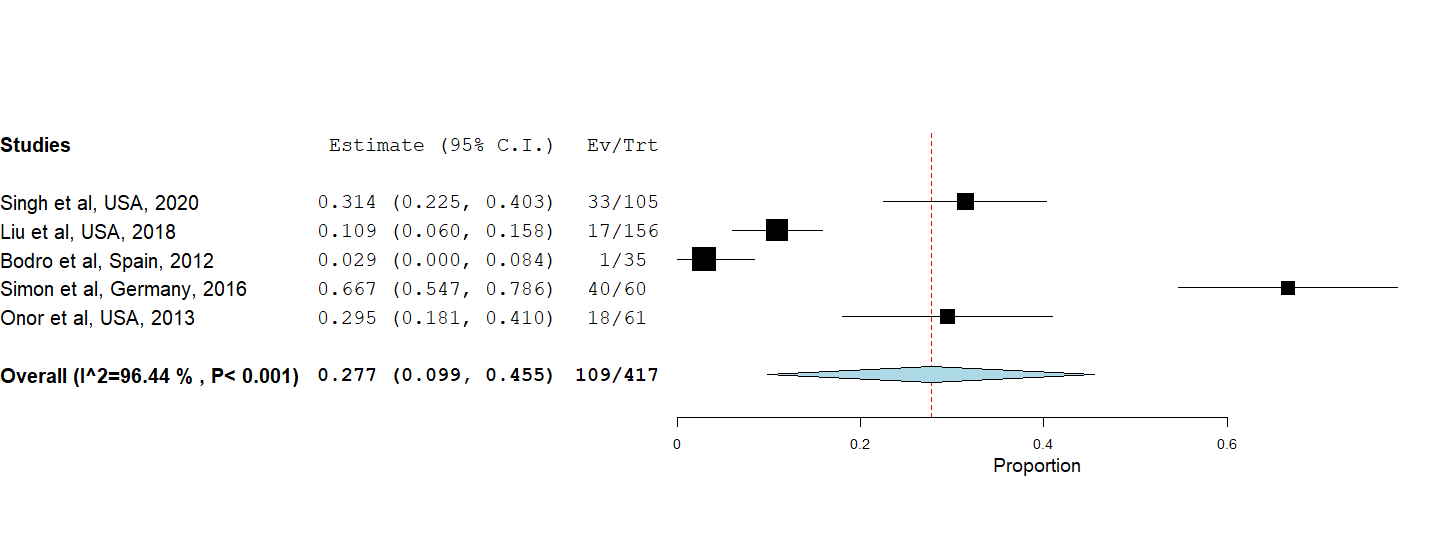 |
| --- |

**Supplemental figure 4.2** Incidence of opportunistic infection in the overall LT recipients undergoing preemptive therapy.

| 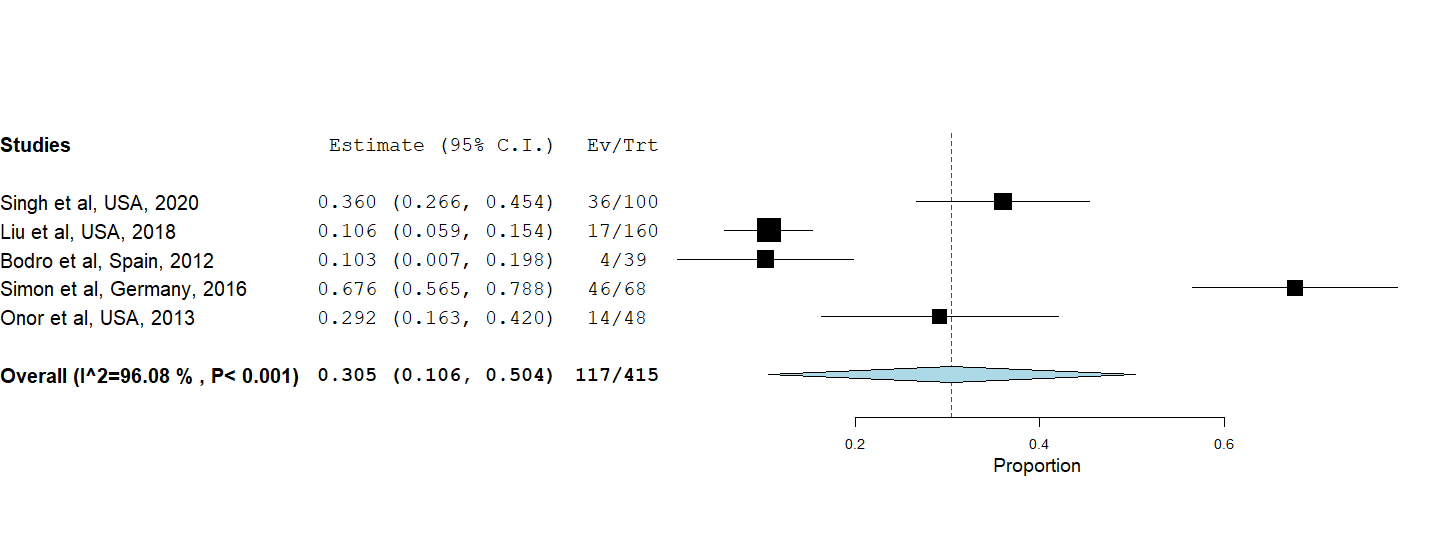 |
| --- |

**Supplemental figure 4.3** Incidence of opportunistic infection in the high-risk group LT recipients undergoing antiviral prophylaxis.

| 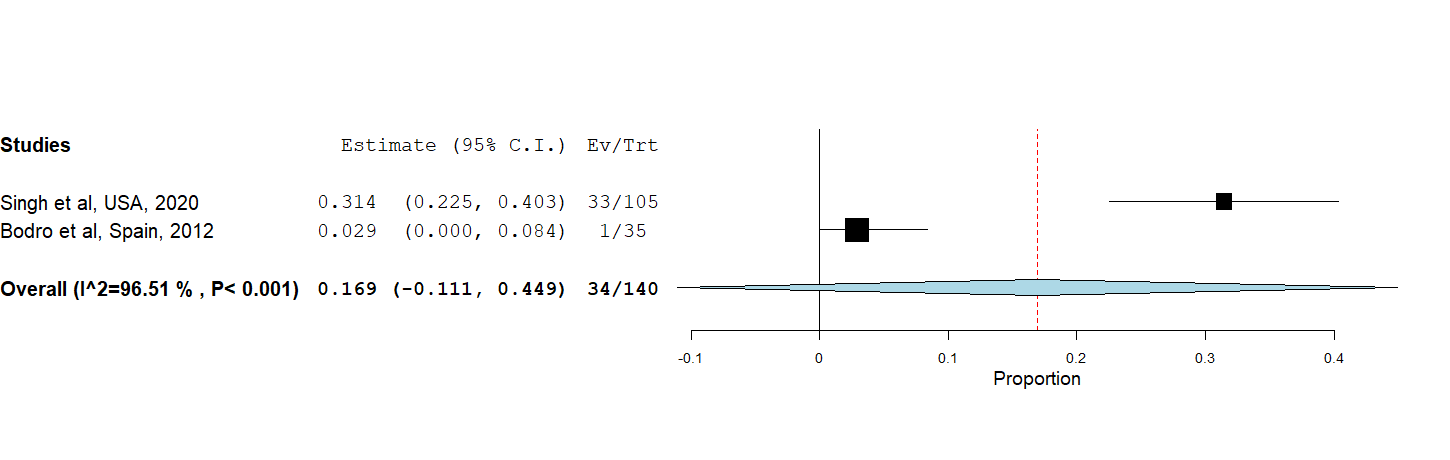 |
| --- |

**Supplemental figure 4.4** Incidence of opportunistic infection in the high-risk group LT recipients undergoing preemptive therapy.

| 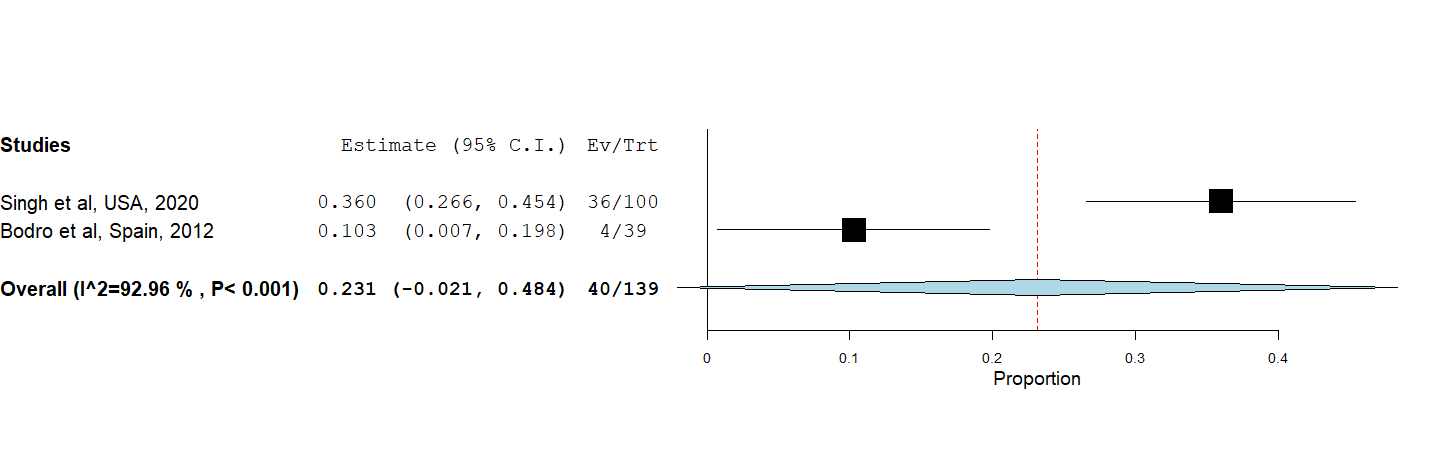 |
| --- |

**Supplemental figure 5:** **Incidence of acute antibody-mediated rejection (AMR)**

**Supplemental figure 5.1** Incidence of acute antibody-mediated rejection (AMR) in the overall LT recipients undergoing antiviral prophylaxis.

| 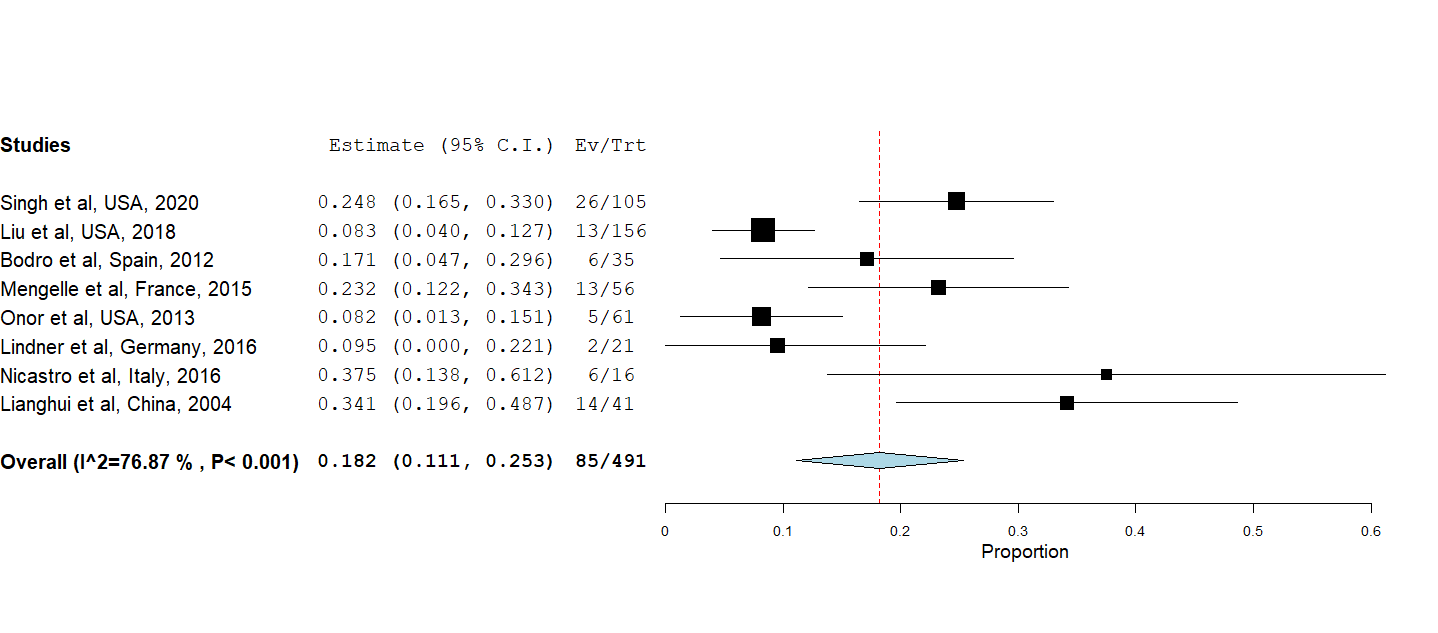 |
| --- |

**Supplemental figure 5.2** Incidence of acute antibody-mediated rejection (AMR) in the overall LT recipients undergoing preemptive therapy.

| 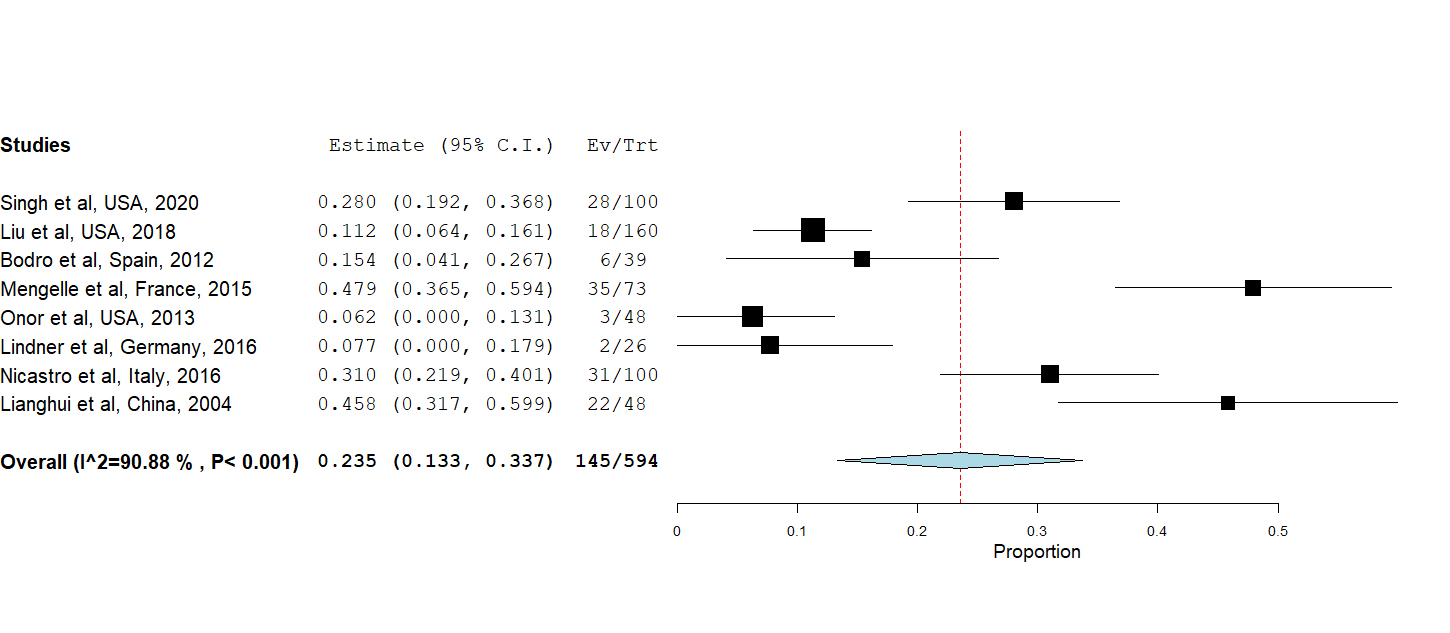 |
| --- |

**Supplemental figure 5.3** Incidence of acute antibody-mediated rejection (AMR) in the high-risk group LT recipients undergoing antiviral prophylaxis.

| 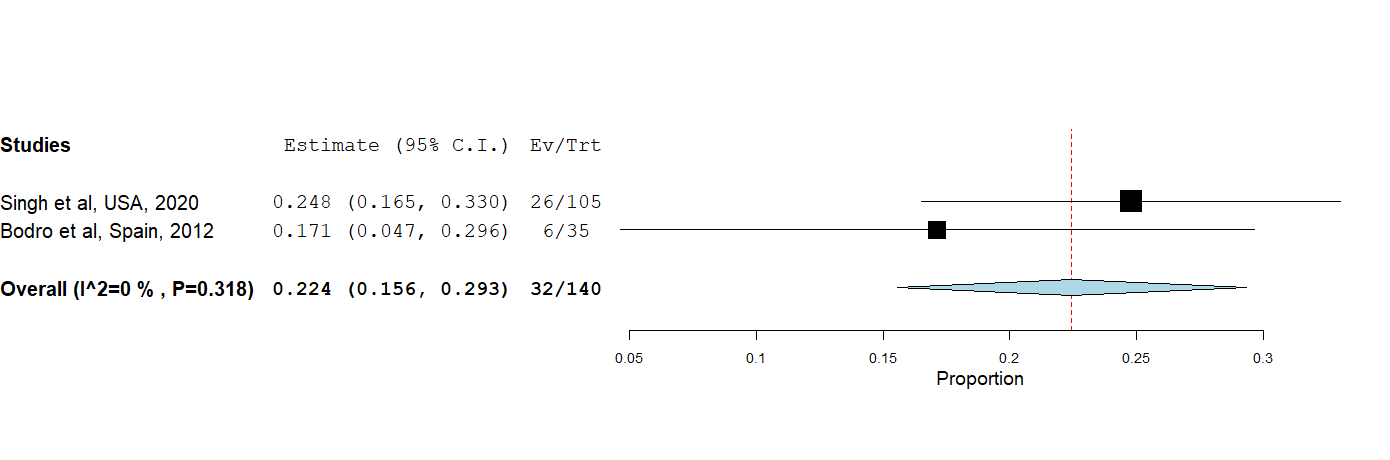 |
| --- |

**Supplemental figure 5.4** Incidence of acute antibody-mediated rejection (AMR) in the high-risk group LT recipients undergoing preemptive therapy.

| 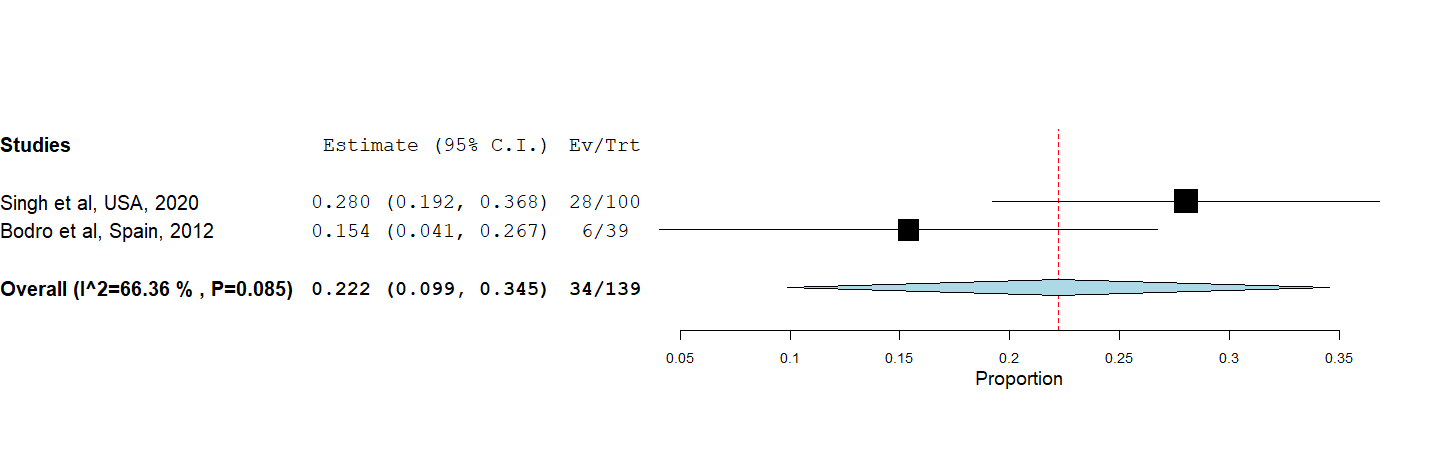 |
| --- |

**Supplemental figure 5.5** Incidence of acute antibody-mediated rejection (AMR) in the intermediate-risk group LT recipients undergoing antiviral prophylaxis.

| 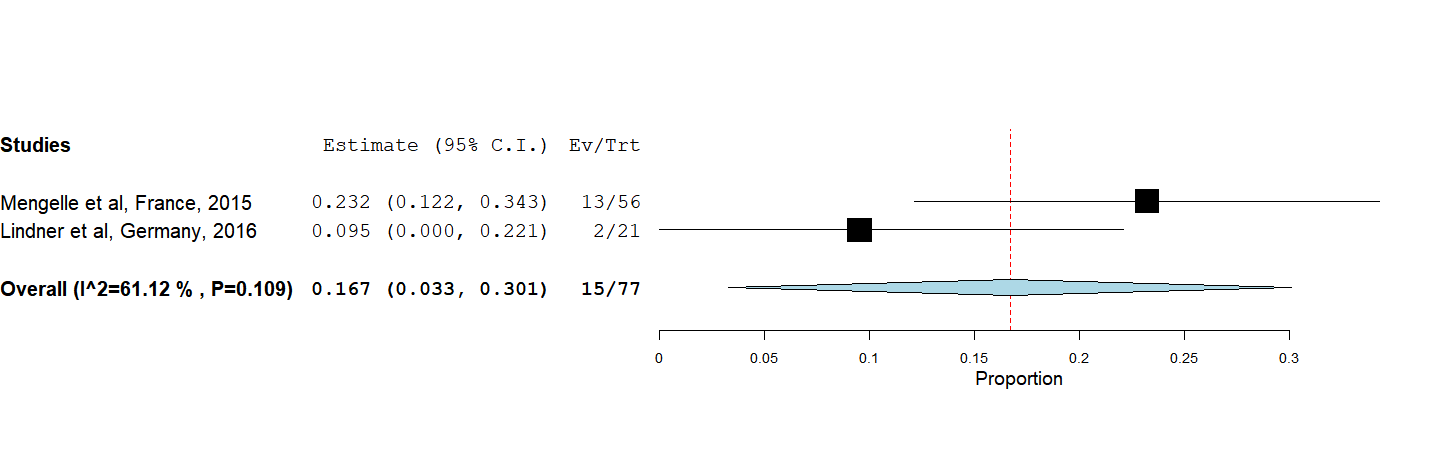 |
| --- |

**Supplemental figure 5.6** Incidence of acute antibody-mediated rejection (AMR) in the intermediate-risk group LT recipients undergoing preemptive therapy.

| 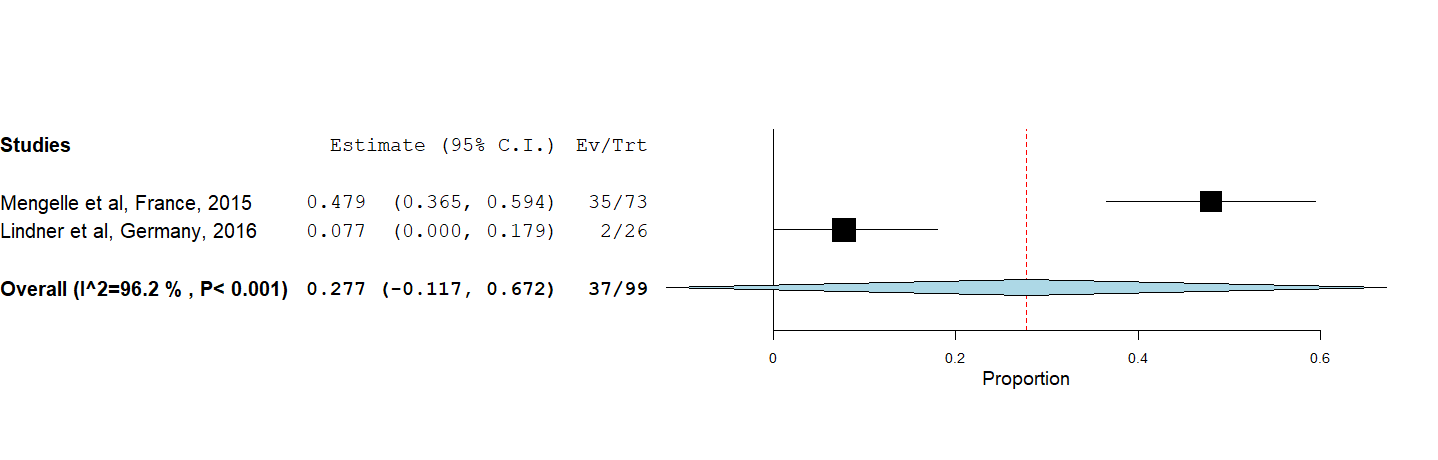 |
| --- |

**Supplemental figure 6:** **Incidence of graft loss**

**Supplemental figure 6.1** Incidence of graft loss in the overall LT recipients undergoing antiviral prophylaxis.

| 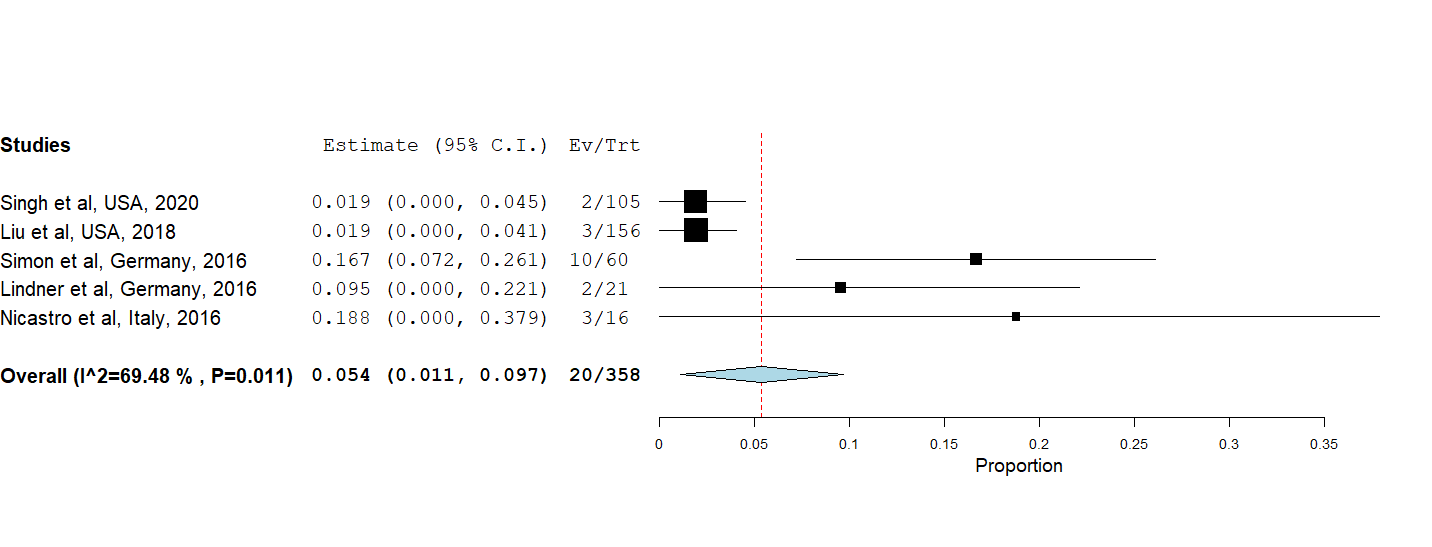 |
| --- |

**Supplemental figure 6.2** Incidence of graft loss in the overall LT recipients undergoing preemptive therapy.

| 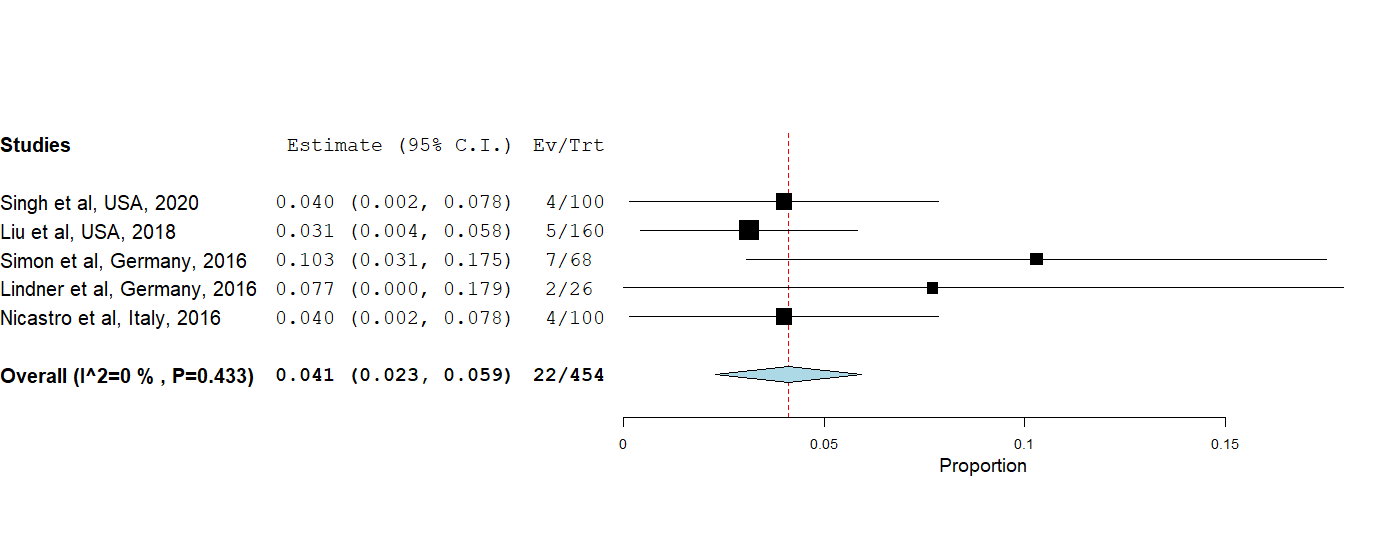 |
| --- |

**Supplemental figure 6.3** Incidence of graft loss in the intermediate-risk group LT recipients undergoing antiviral prophylaxis.

| 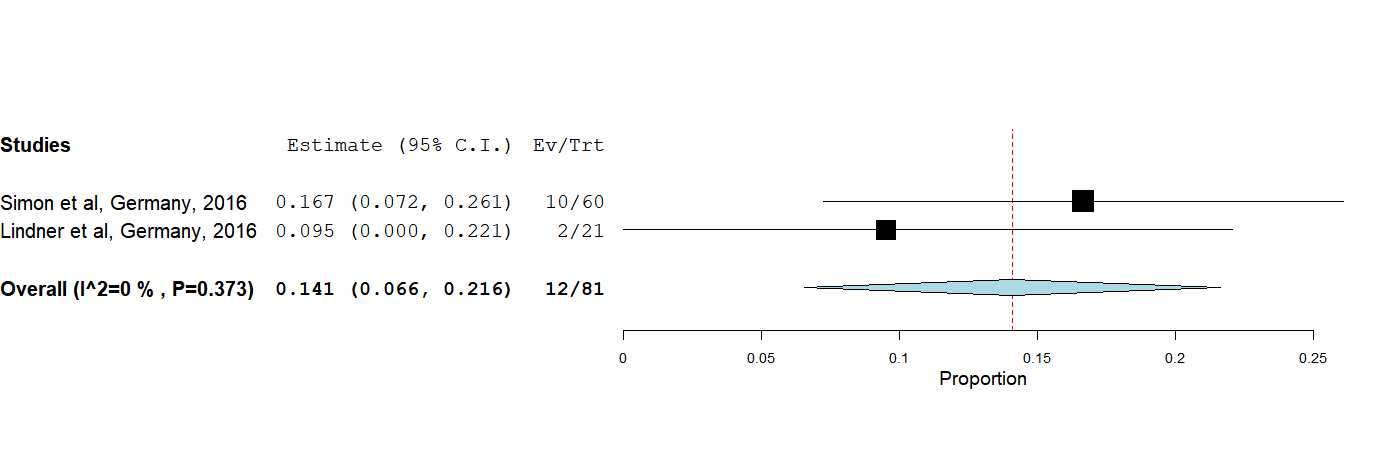 |
| --- |

**Supplemental figure 6.4** Incidence of graft loss in the intermediate-risk group LT recipients undergoing preemptive therapy.

| 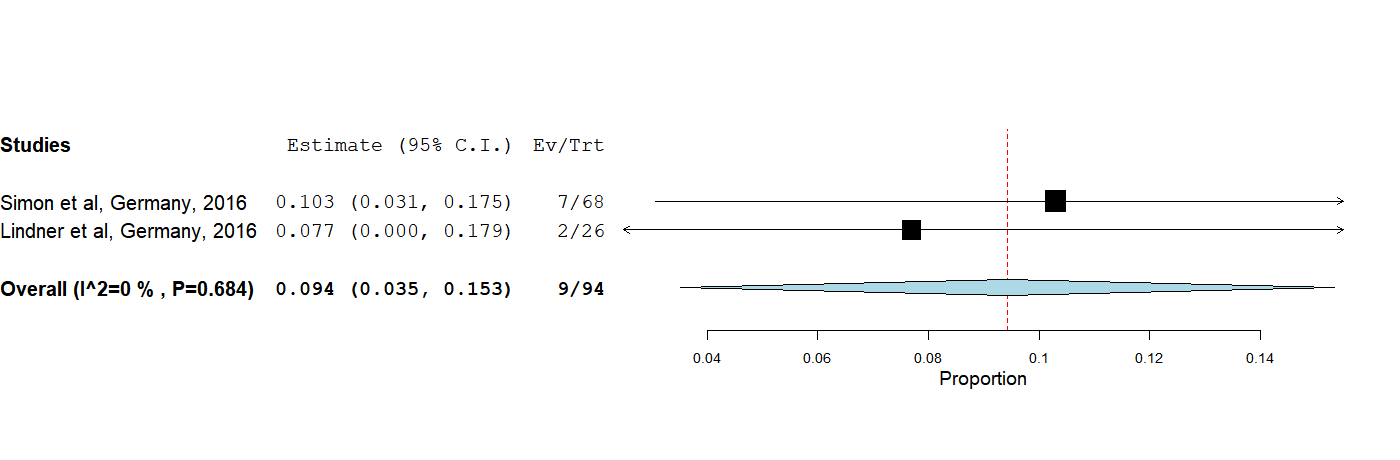 |
| --- |

**Supplemental figure 7: Incidence of leukopenia**

**Supplemental figure 7.1** Incidence of leukopenia in the overall LT recipients undergoing antiviral prophylaxis.

| 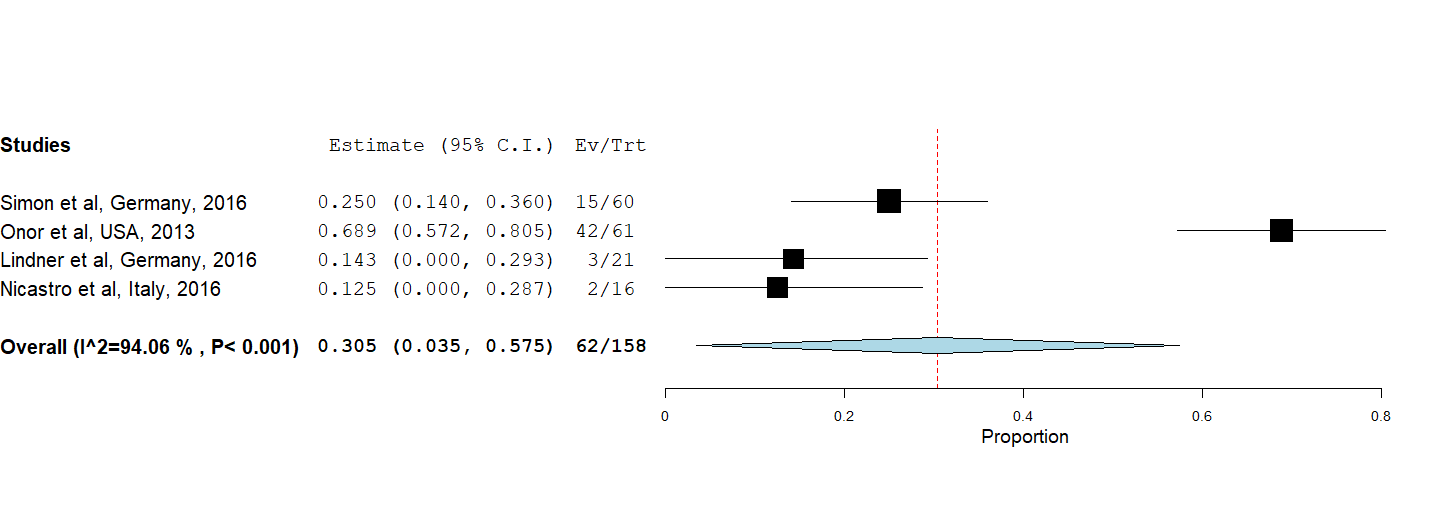 |
| --- |

**Supplemental figure 7.2** Incidence of leukopenia in the overall LT recipients undergoing preemptive therapy.

| 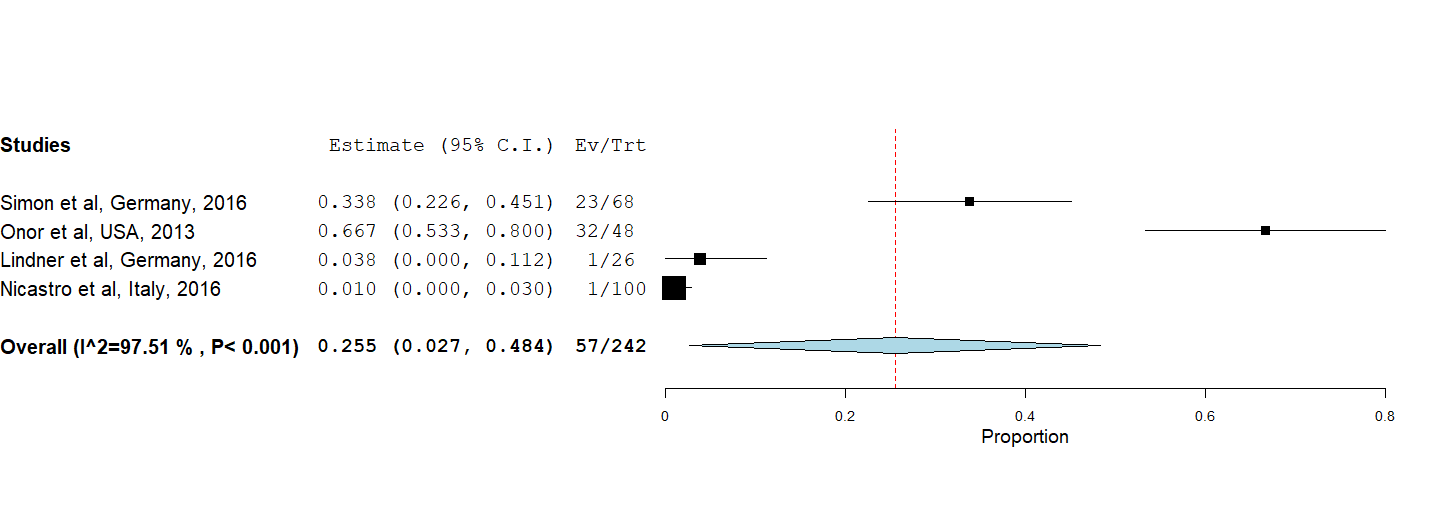 |
| --- |

**Supplemental figure 7.3** Incidence of leukopenia in the intermediate-risk group LT recipients undergoing antiviral prophylaxis.

| 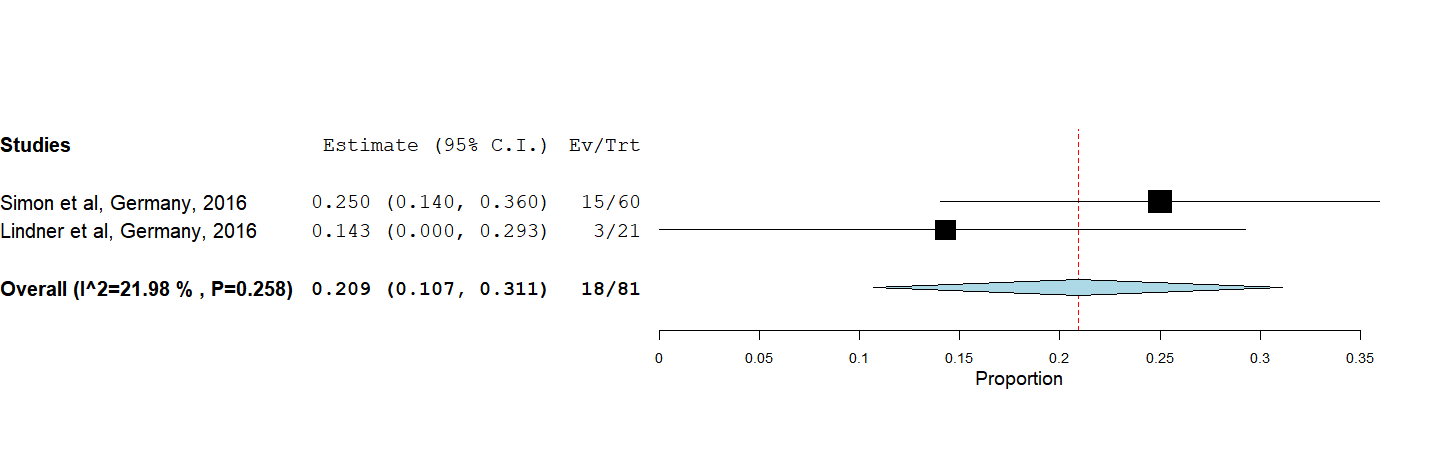 |
| --- |

**Supplemental figure 7.4** Incidence of leukopenia in the intermediate-risk group LT recipients undergoing preemptive therapy.

| 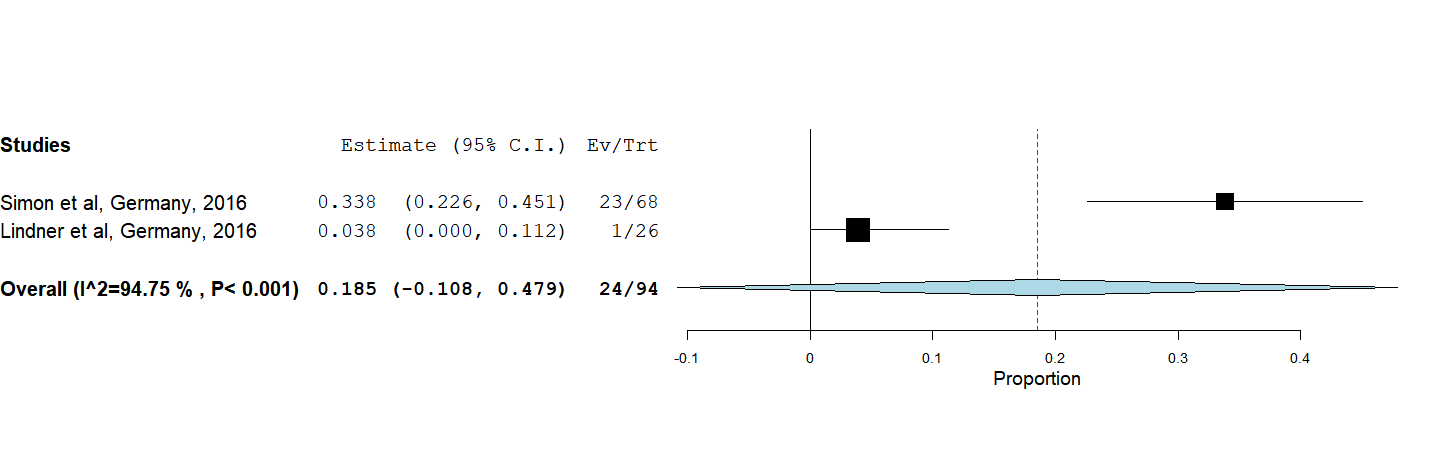 |
| --- |

**Supplemental figure 8:** **Incidence of neutropenia**

**Supplemental figure 8.1** Incidence of neutropenia in the overall LT recipients undergoing antiviral prophylaxis.

| 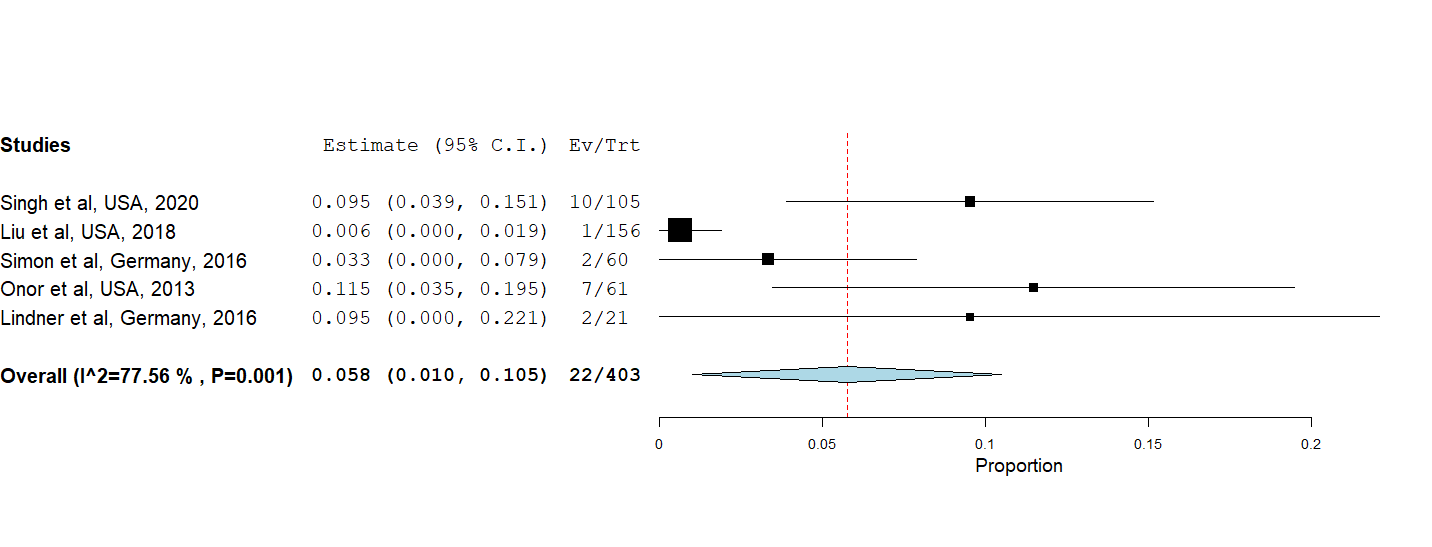 |
| --- |

**Supplemental figure 8.2** Incidence of neutropenia in the overall LT recipients undergoing preemptive therapy.

| 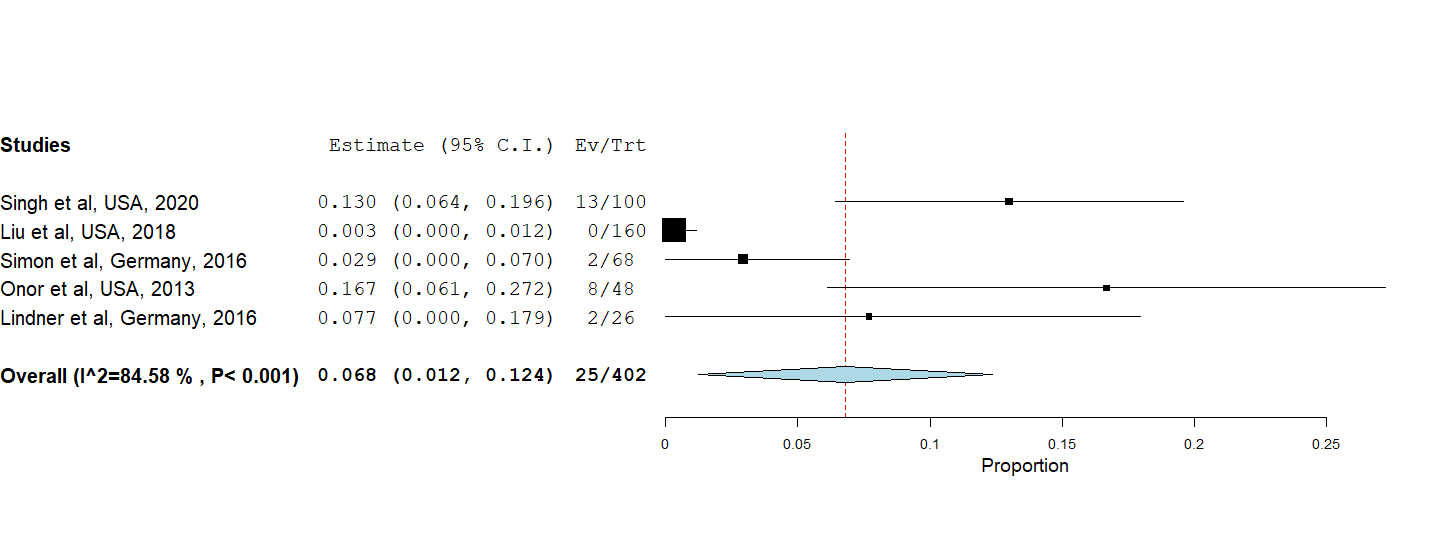 |
| --- |

**Supplemental figure 8.3** Incidence of neutropenia in the intermediate-risk group LT recipients undergoing antiviral prophylaxis.

| 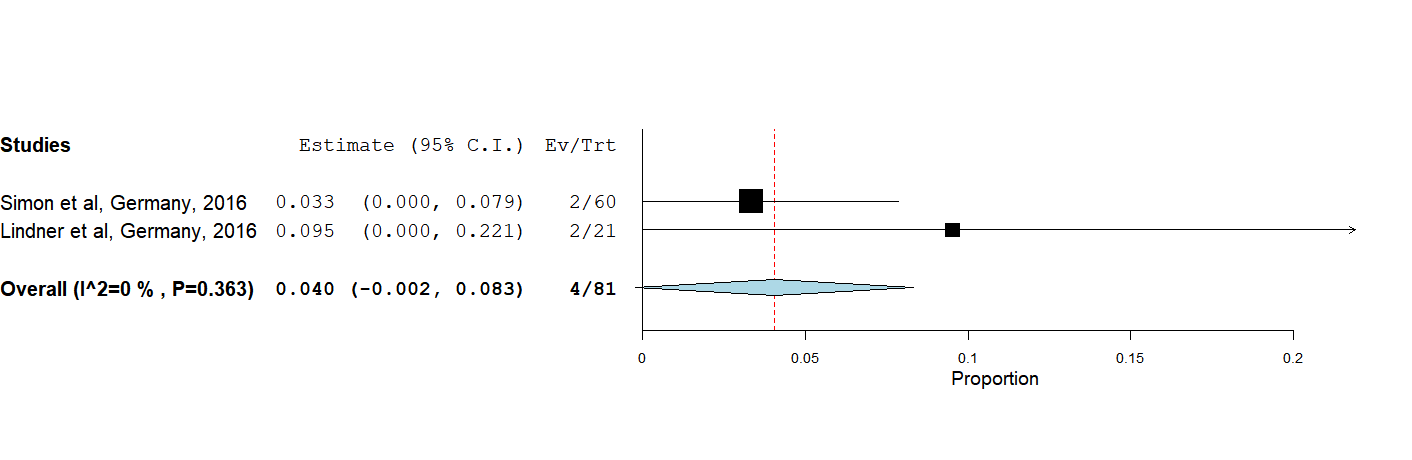 |
| --- |

**Supplemental figure 8.4** Incidence of neutropenia in the intermediate-risk group LT recipients undergoing preemptive therapy.

| 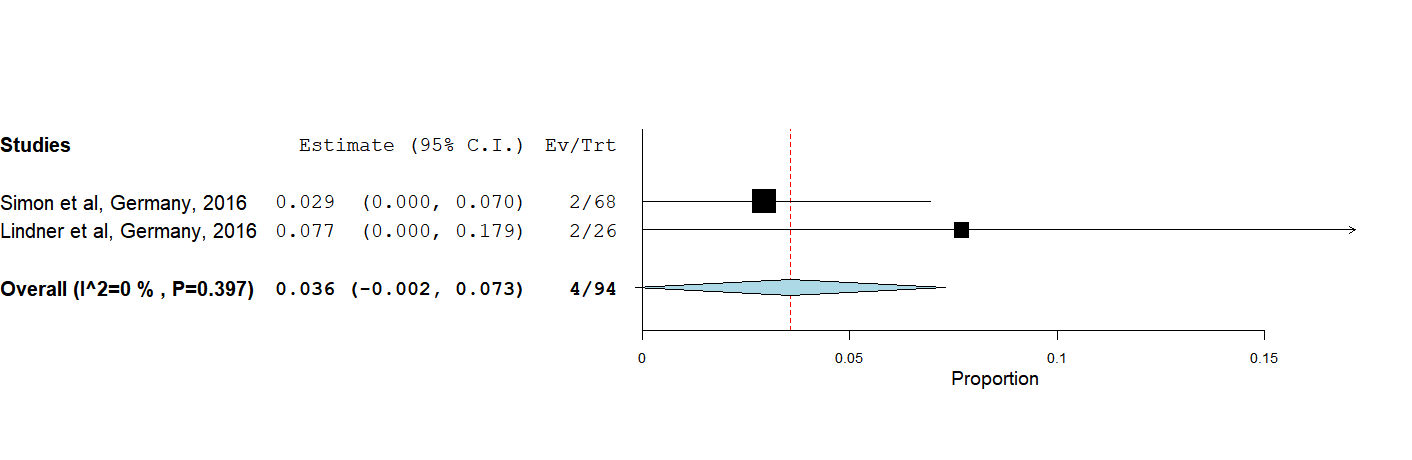 |
| --- |

**Supplemental figure 9: Incidence of late-onset CMV diseas**e

**Supplemental figure 9.1** Incidence of late-onset CMV disease in the overall LT recipients undergoing antiviral prophylaxis.

| 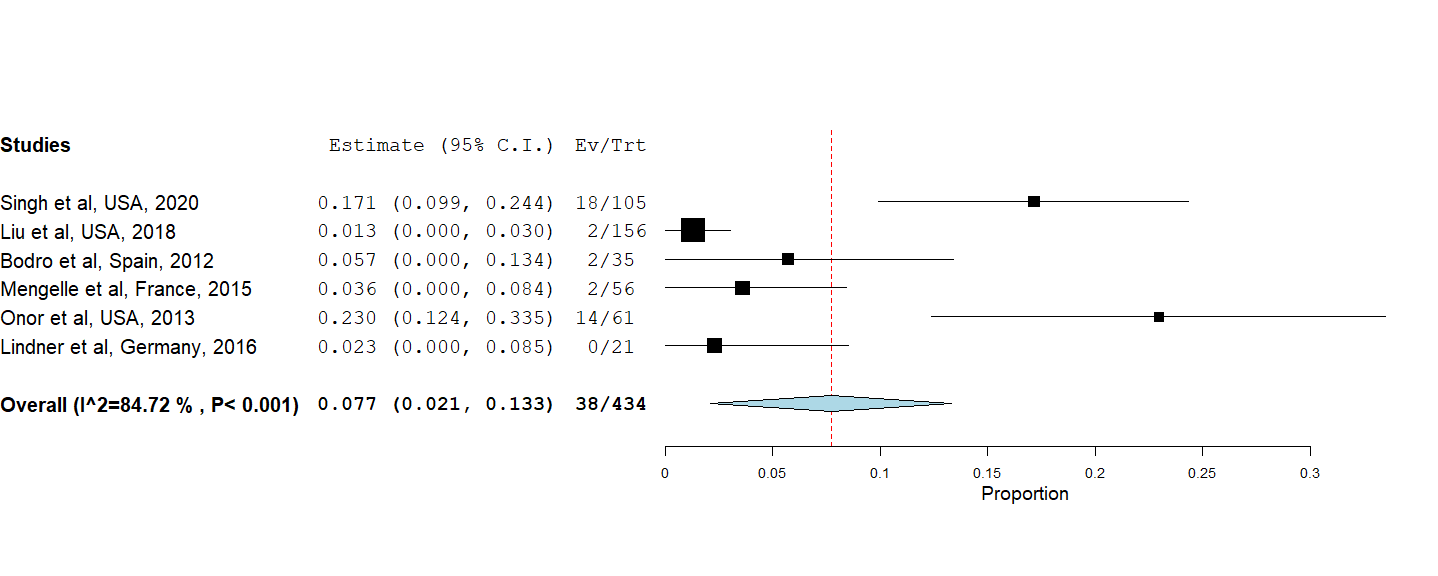 |
| --- |

**Supplemental figure 9.2** Incidence of late-onset CMV disease in the overall LT recipients undergoing preemptive therapy.

| 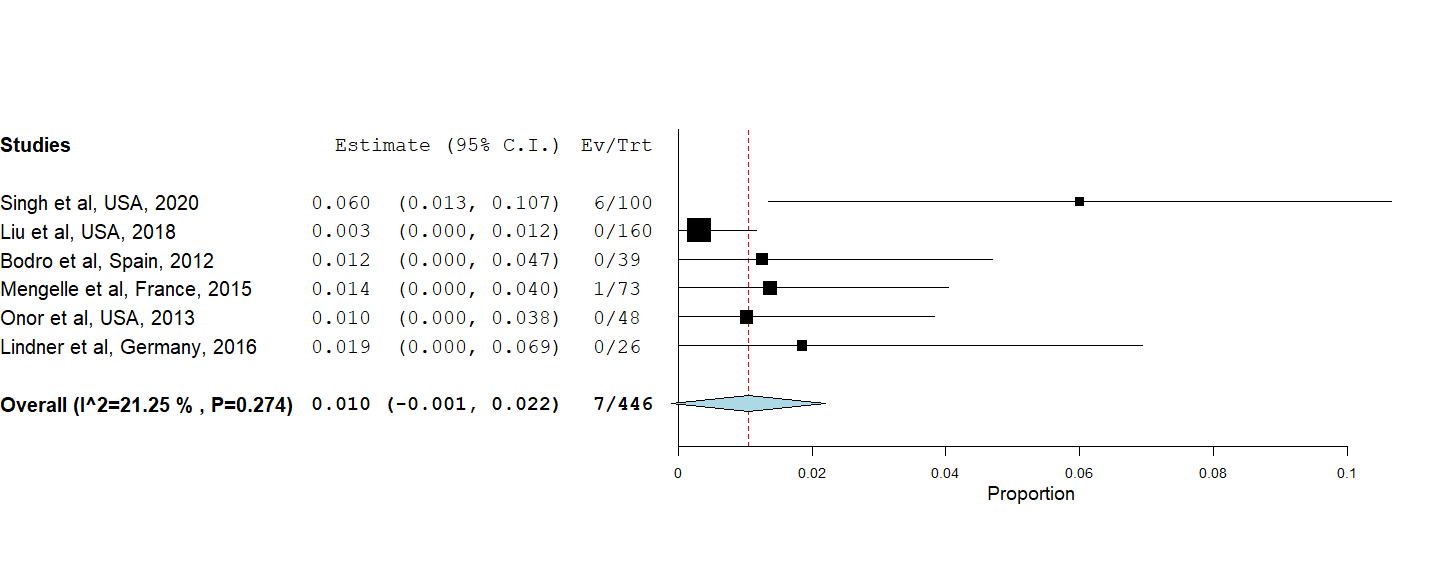 |
| --- |

**Supplemental figure 9.3** Incidence of late-onset CMV disease in the high-risk group LT recipients undergoing antiviral prophylaxis.

| 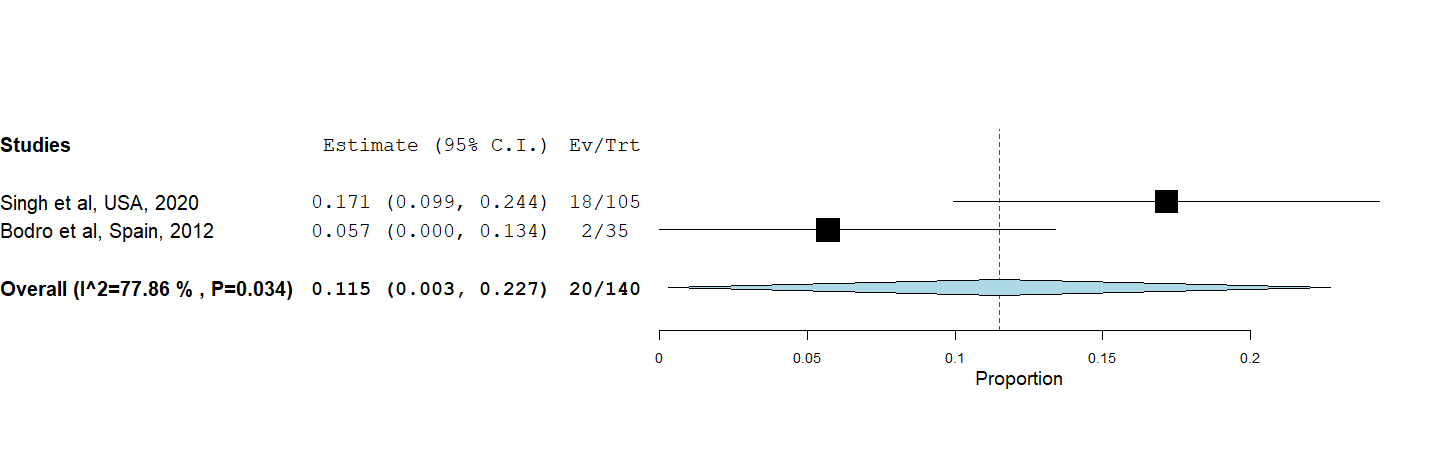 |
| --- |

**Supplemental figure 9.4** Incidence of late-onset CMV disease in the high-risk group LT recipients undergoing preemptive therapy.

| 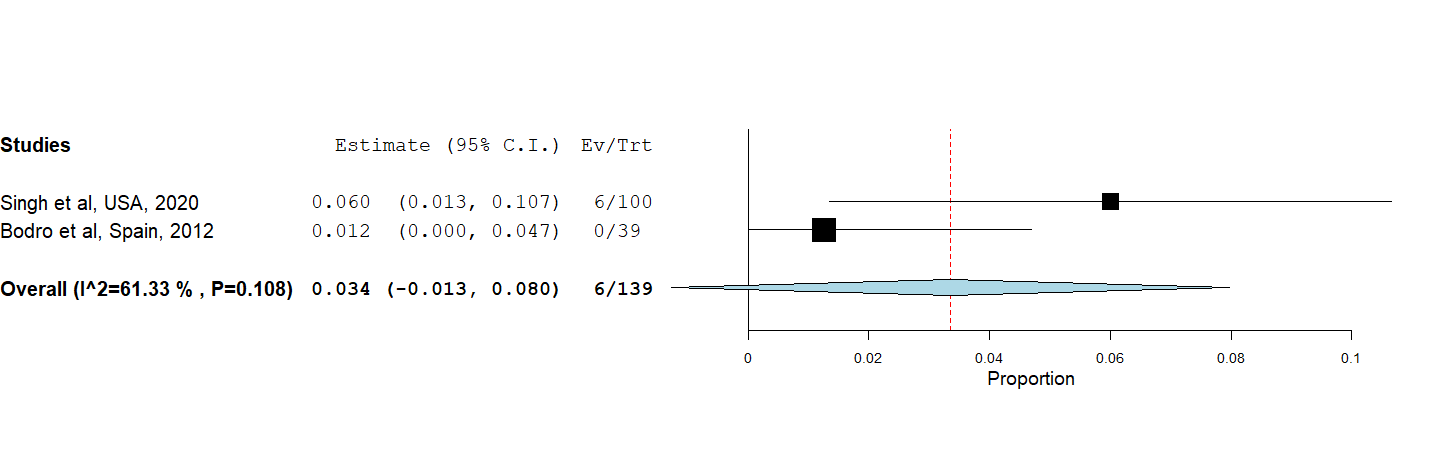 |
| --- |

**Supplemental figure 9.5** Incidence of late-onset CMV disease in the intermediate-risk group LT recipients undergoing antiviral prophylaxis.

| 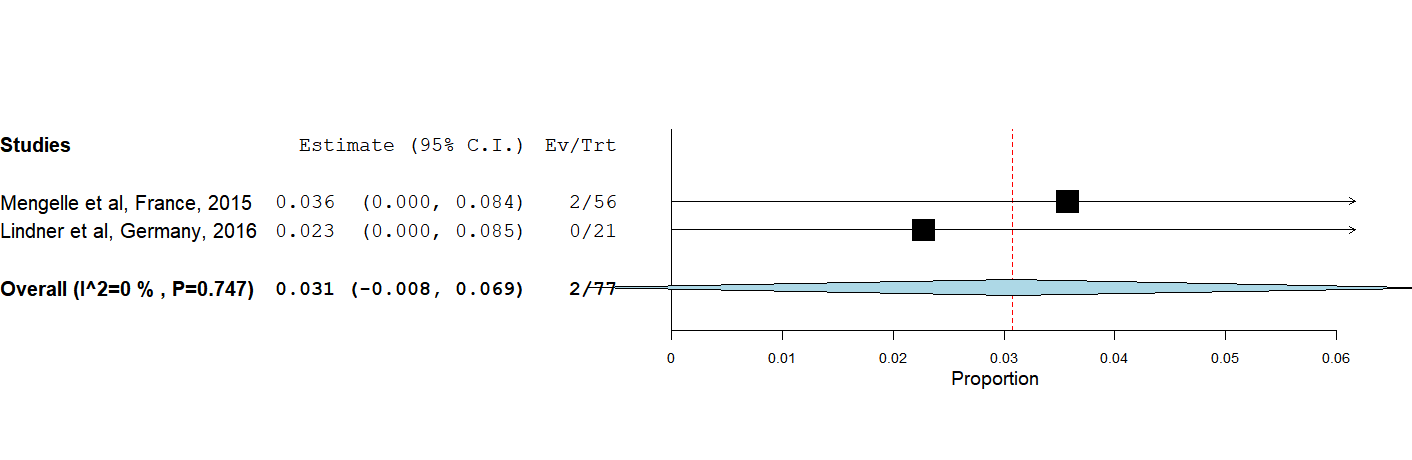 |
| --- |

**Supplemental figure 9.6** Incidence of late-onset CMV disease in the intermediate-risk group LT recipients undergoing preemptive therapy.

| 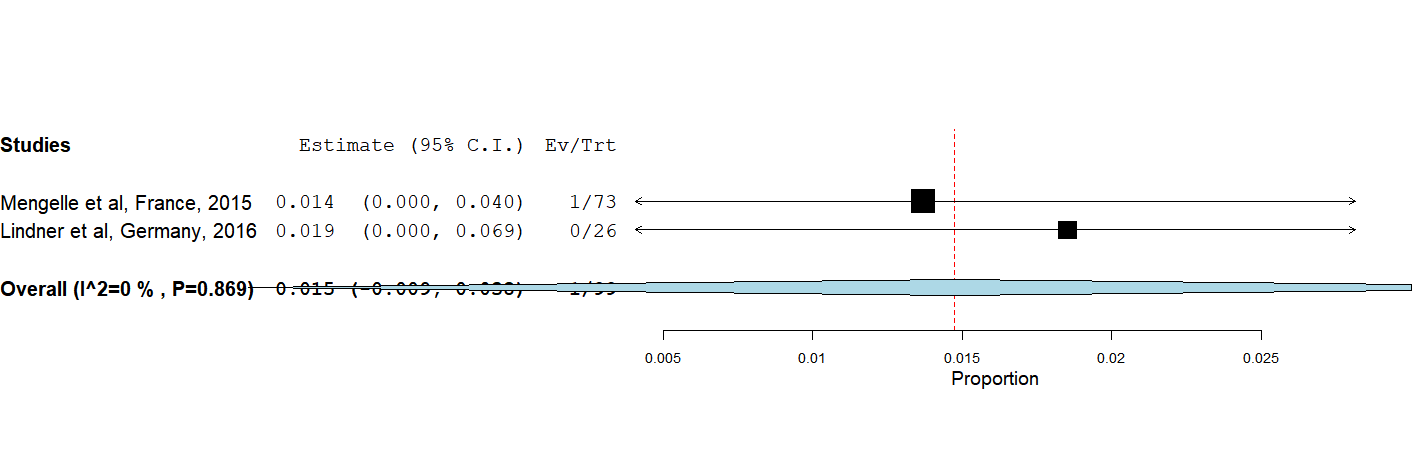 |
| --- |

**Supplemental figure 10: Incidence of mortality**

**Supplemental figure 10.1** Incidence of mortality in the overall LT recipients undergoing antiviral prophylaxis.

| 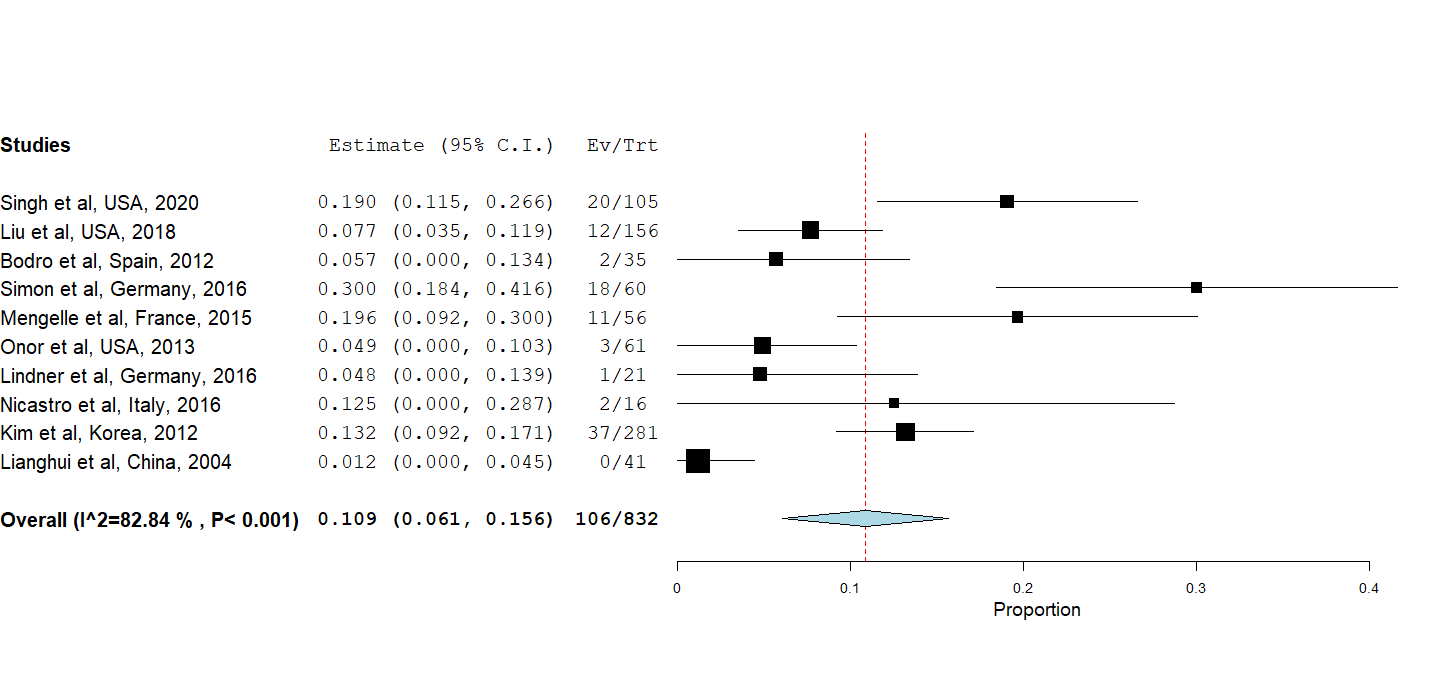 |
| --- |

**Supplemental figure 10.2** Incidence of mortality in the overall LT recipients undergoing preemptive therapy.

| 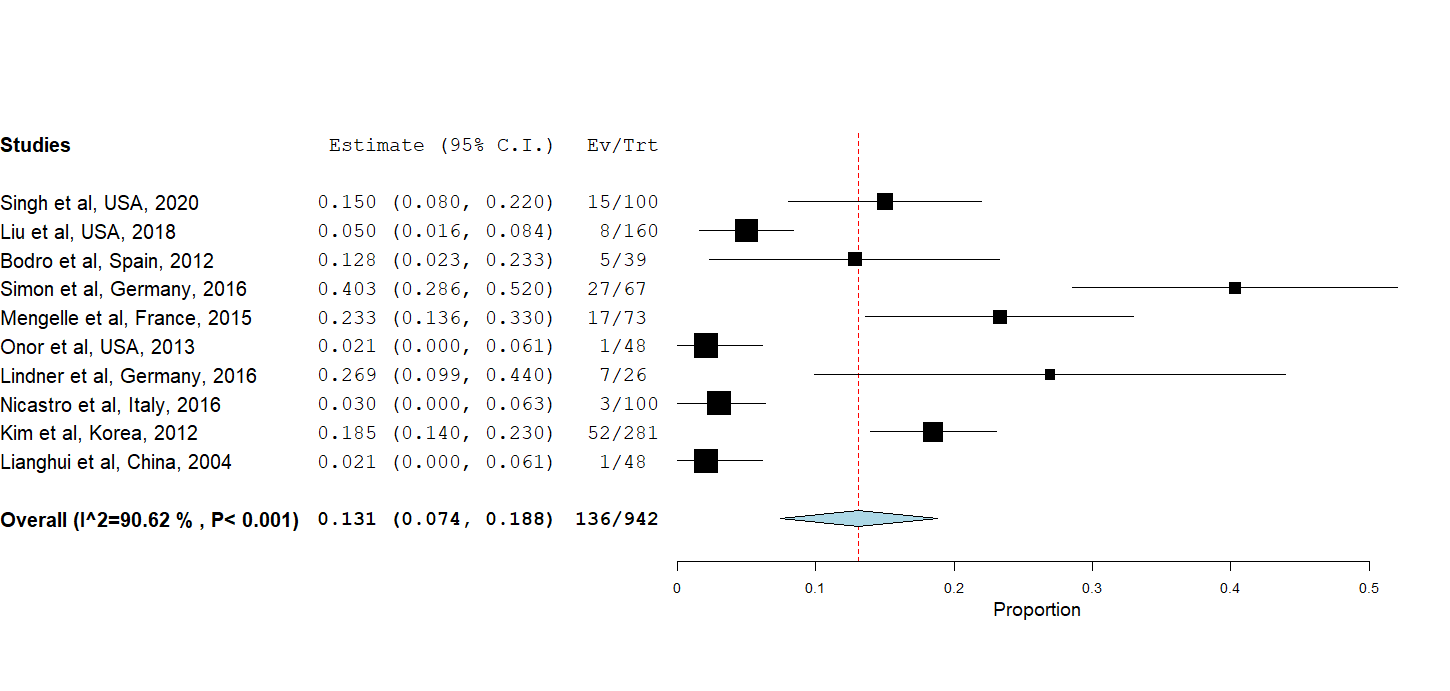 |
| --- |

**Supplemental figure 10.3** Incidence of mortality in the high-risk group LT recipients undergoing antiviral prophylaxis.

| 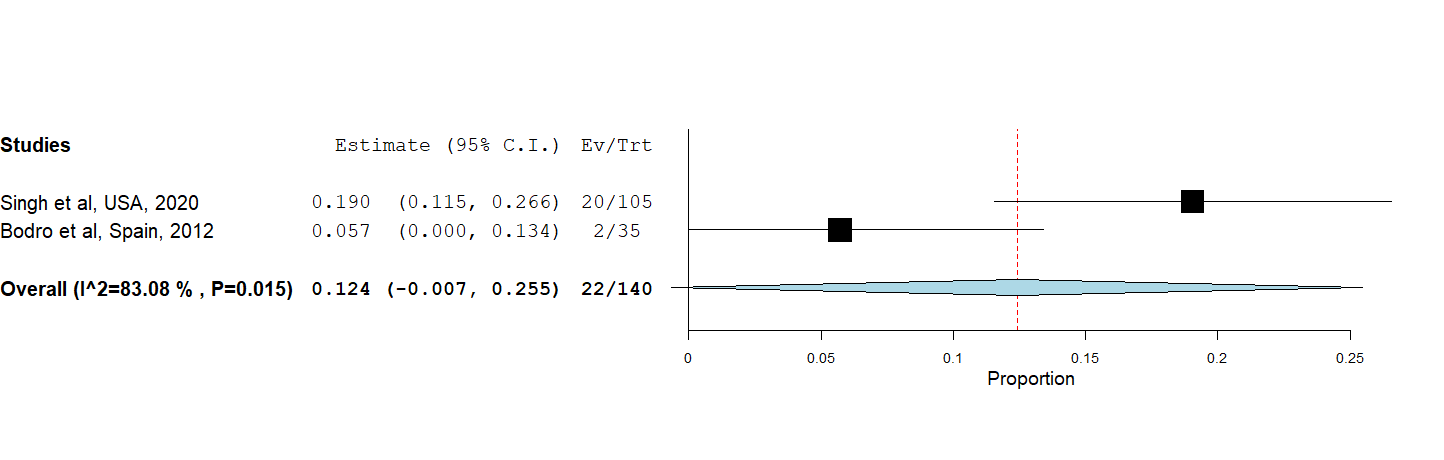 |
| --- |

**Supplemental figure 10.4** Incidence of mortality in the high-risk group LT recipients undergoing preemptive therapy.

| 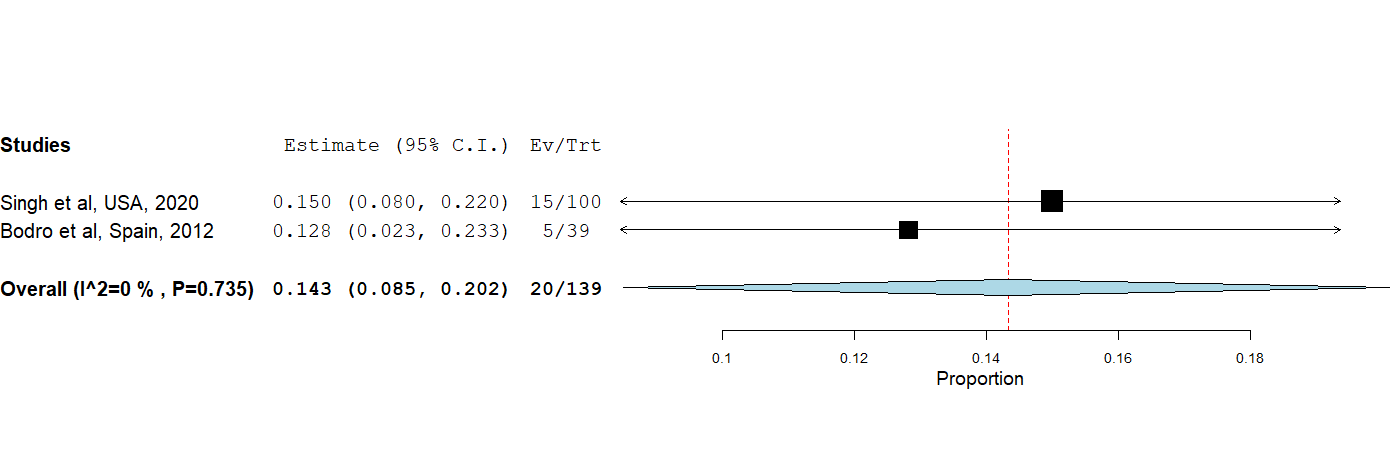 |
| --- |

**Supplemental figure 10.5** Incidence of mortality in the intermediate-risk group LT recipients undergoing antiviral prophylaxis.

| 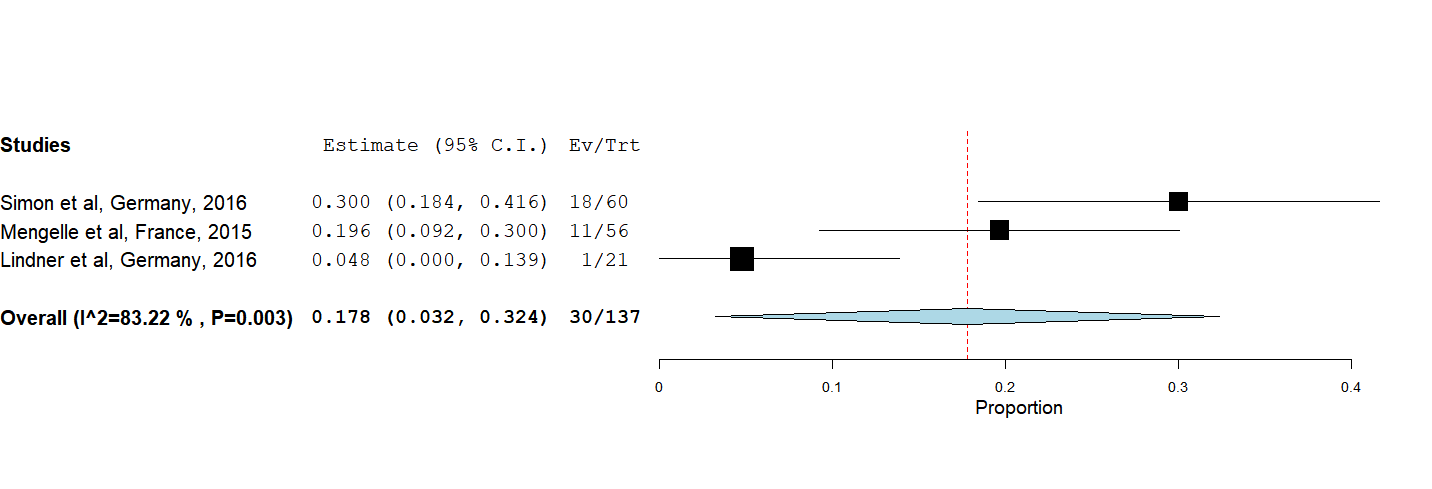 |
| --- |

**Supplemental figure 10.6** Incidence of mortality in the intermediate-risk group LT recipients undergoing preemptive therapy.

| 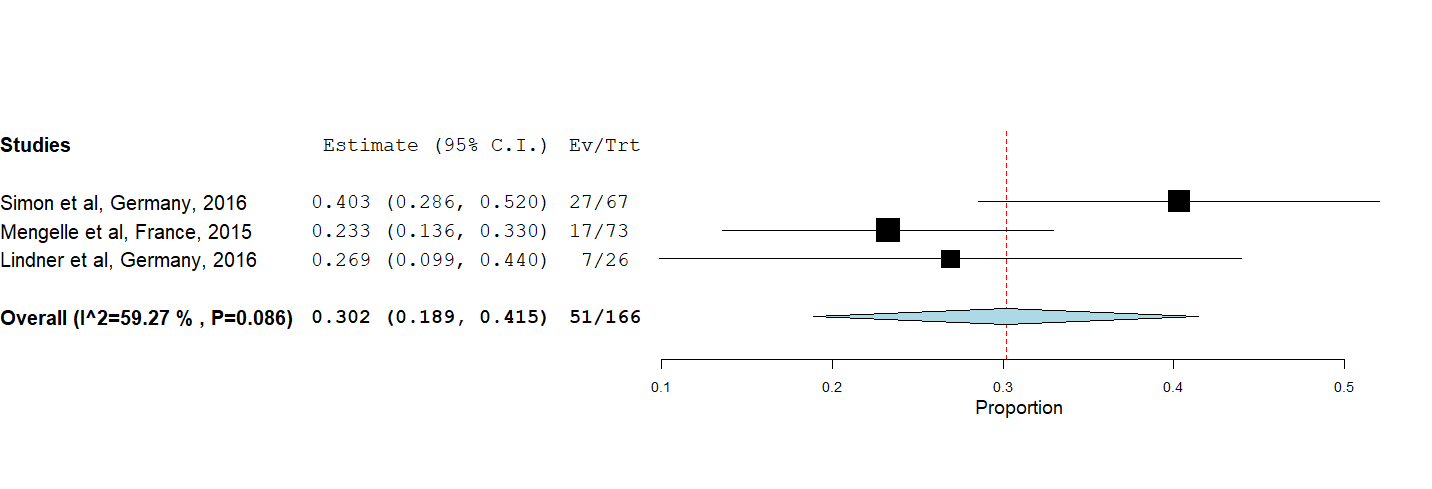 |
| --- |

**Supplemental figure 11:** **Incidence of CMV related mortality**

**Supplemental figure 11.1** Incidence of CMV related mortality in the overall LT recipients undergoing antiviral prophylaxis.

| 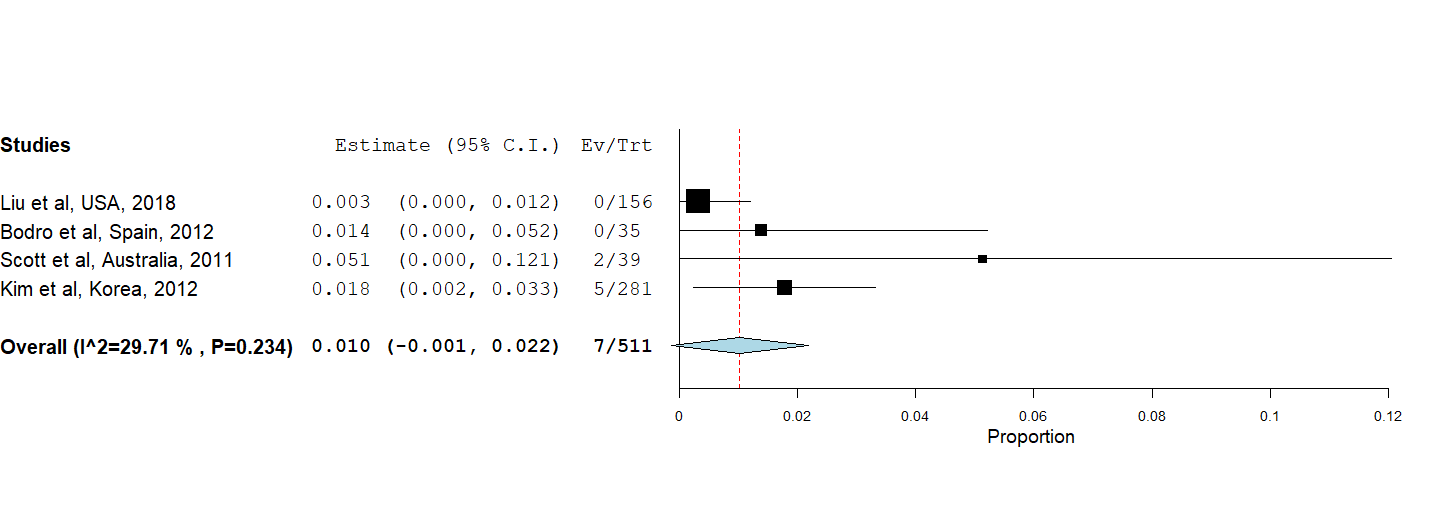 |
| --- |

**Supplemental figure 11.2** Incidence of CMV related mortality in the overall LT recipients undergoing preemptive therapy.

| 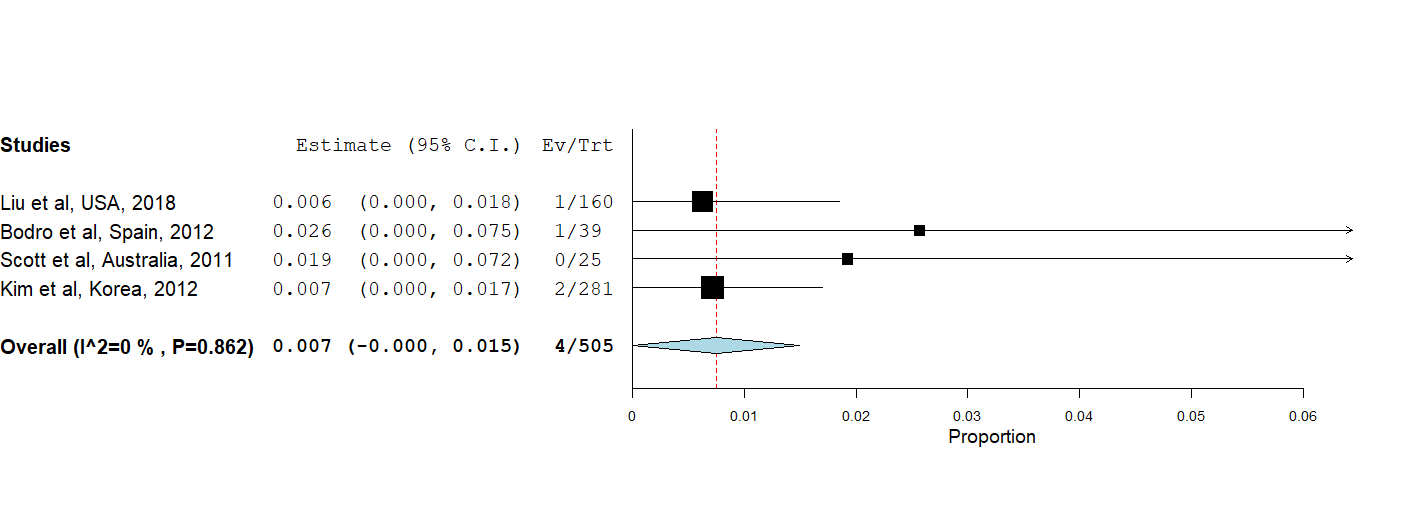 |
| --- |

**Supplemental figure 12:** **Incidence of the development of drug resistance**

**Supplemental figure 12.1** Incidence of drug resistance development in the overall LT recipients undergoing antiviral prophylaxis.

| 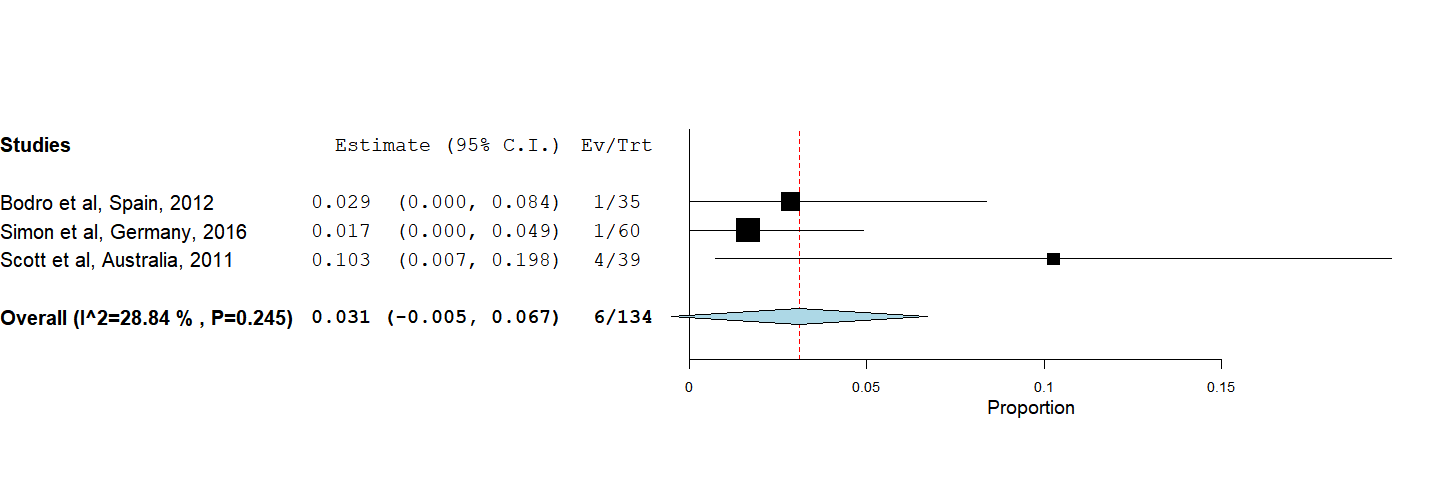 |
| --- |

**Supplemental figure 12.2** Incidence of the development of drug resistance in the overall LT recipients undergoing preemptive therapy.

| 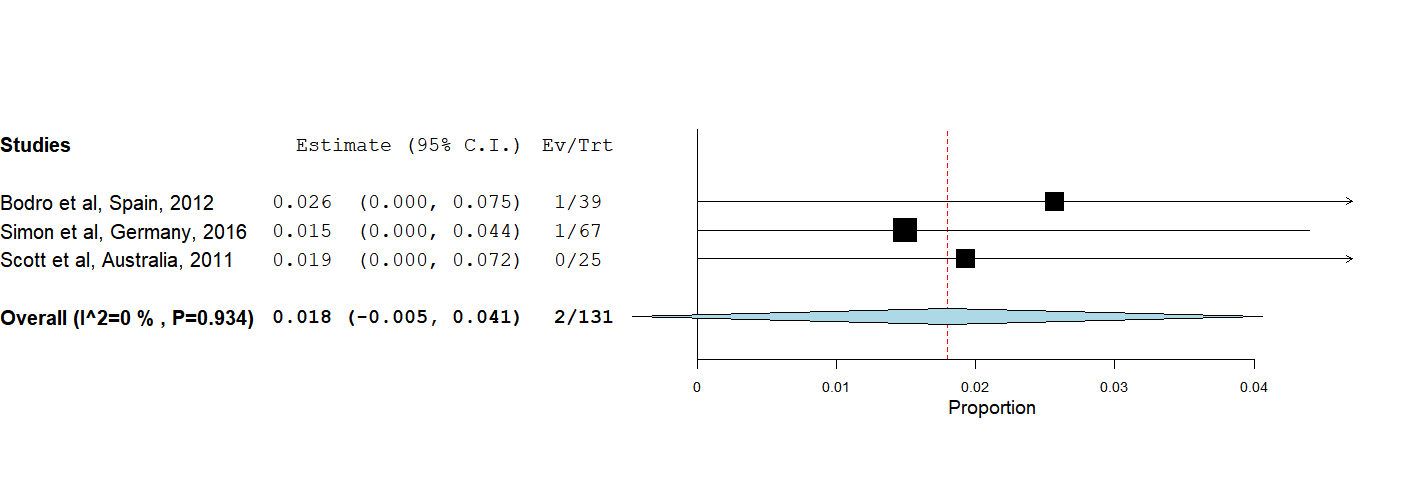 |
| --- |

**Supplemental figure 13**

**Supplemental figure 13.1** Forest plot depicts a comparison of the incidence of CMV specific neutralizing antibodies among LT recipients undergoing antiviral prophylaxis and preemptive therapy.

| 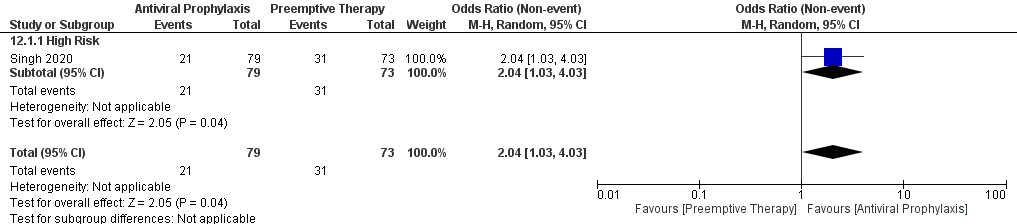 |
| --- |

**Supplemental figure 13.2** Forest plot depicts a comparison of the incidence of the development of drug resistance among LT recipients undergoing antiviral prophylaxis and preemptive therapy.

| 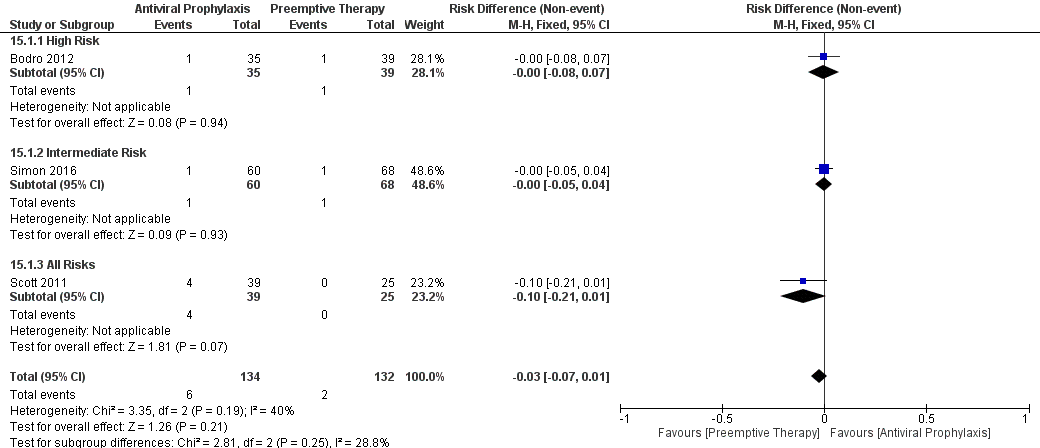 |
| --- |

**Supplemental figure 13.3** Forest plot depicts a comparison of the incidence of chronic rejection among LT recipients undergoing antiviral prophylaxis and preemptive therapy.

| 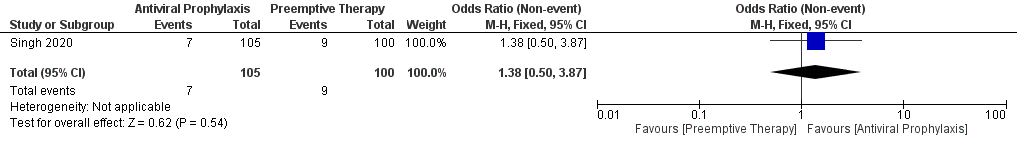 |
| --- |
